# Vernonieae (Asteraceae) of southern Africa: A generic disposition of the species and a study of their pollen

**DOI:** 10.3897/phytokeys.60.6734

**Published:** 2016-02-11

**Authors:** Harold Robinson, John J. Skvarla, Vicki A. Funk

**Affiliations:** 1 Dept. of Botany, MRC 166, NMNH, P.O. Box 37012, Smithsonian Institution, Washington, DC. 20013-7012, USA; 2 Dept. of Botany and Microbiology, and Oklahoma Biological Survey, University of Oklahoma, Norman, Oklahoma, 73019-6131, USA; 3 Dept. of Botany, MRC 166, NMNH, P.O. Box 37012, Smithsonian Institution, Washington, DC. 20013-7012, USA

**Keywords:** Asteraceae, *Baccharoides*, *Bothriocline*, Botswana, Compositae, *Cyanthillium*, *Distephanus*, *Erlangea*, *Ethulia*, *Gymnanthemum*, *Hilliardiella*, Lesotho, Namibia, *Namibithamnus*, new combinations, new genera, *Oocephala*, *Orbivestus*, *Parapolydora*, pollen, *Polydora*, *Pseudopegolettia*, South Africa, Swaziland, *Vernonella*, *Vernonia*, *Vernoniastrum*, Vernonieae

## Abstract

Current and previously included members of the Tribe Vernonieae (Asteraceae) of southern Africa are listed in their presently recognized genera with complete synonymies and keys to genera and species. The genus *Vernonia*, as presently delimited, does not occur in Africa. Genera of the Vernonieae presently recognized from southern Africa are *Baccharoides*, *Bothriocline*, *Cyanthillium*, *Distephanus*, *Erlangea*, *Ethulia*, *Gymnanthemum*, *Hilliardiella*, *Oocephala*, *Orbivestus*, *Parapolydora*, *Polydora*, *Vernonella*, *Vernoniastrum*, plus two genera that are named as new: *Namibithamnus* and *Pseudopegolettia*. Twelve new combinations are provided and two species, *Vernonia potamiphila* and *Vernonia collinii* Klatt., hom. illeg., remain unplaced because of a lack of material.

Pollen types are illustrated including previously recognized types: non-lophate, sublophate, tricolporate lophate, and non-colpate triporate lophate. A type previously unknown in the Asteraceae is described here and in a separate paper for *Oocephala* and *Polydora*; a non-colpate pantoporate lophate type with pores not strictly equatorial.

## Introduction

Attempts to revise the generic concepts of the tribe Vernonieae (Asteraceae: subfamily Cichorioideae) in Africa have proven difficult, but it is now possible to resolve nearly all of the generic limits within the tribe in the more limited area of southern Africa here defined as including the following: Botswana, Lesotho, Namibia, Republic of South Africa, and Swaziland. This treatment is the latest in a series of papers revising the generic limits in the Vernonieae, a series that includes an initial summary of eastern hemisphere taxa (Robinson 1999a), a summary of western hemisphere taxa, (Robinson 1999b), the Vernonieae of China (Robinson and Skvarla 2010) and the Vernonieae of Thailand (Bunwong et al. 2014). As elsewhere in the series, changes are necessitated by the discovery of the natural limits of the genus *Vernonia* Schreb., typified by *Vernonia noveboracensis* (L.) Michx., which is now known to be almost entirely restricted to North America (Robinson 1999a, b) and is native only in the western hemisphere. Thus, many older as well as recent generic segregates in the tribe are now recognized. In addition, in this treatment, two genera are described as new, older names are recognized for two species, and new combinations are provided for 13 species that have previously been placed in *Vernonia*. The pollen of the revised southern African genera is described and illustrated. During the course of the study a new species of *Gymnanthemum* has been found and described elsewhere (Robinson and Funk 2014) and a previously unknown form of pollen for the Asteraceae has been recognized (Robinson and Skvarla 2014).

The initial reference used for members of the Vernonieae in southern Africa was *Flora Capensis* by Harvey and Sonder (1894). Additions have been made using Jeffrey and Leistner (2000), Pooley (1991) and Retief and Hermann (1997), and especially Arnold and De Wet (1993). Also helpful were the treatments of *Flora Zambesiaca* (Wild 1978a, 1978b, Wild and Pope 1977) and Madagascar (Humbert 1960). Subtractions from the Vernonieae, as listed by Harvey and Sonder, include the discovery that *Corymbium* L., *Hoplophyllum* DC., *Litogyne* Harv., and *Platycarpha* Less. are not members of the Vernonieae. According to the latest molecular phylogenies (Keeley and Robinson 2009; Funk et al. 2009), *Corymbium* is in the tribe Corymbieae, at the base of the subfamily Asteroideae, *Hoplophyllum* is in the tribe Eremothamneae in the subfamily Cichorioideae, *Litogyne* is in the tribe Inuleae in the subfamily Asteroideae, and *Platycarpha* is in the tribe Platycarphaeae in the subfamily Cichorioideae. The first three of these genera have totally non-lophate pollen (see below). In southern Africa, the Vernonieae now contain 13 genera. These include *Ethulia*, recognized by Harvey and Sonder, plus the various old and new segregates of *Vernonia* *sensu lato*, i.e. *Distephanus* Cass., *Gymnanthemum* Cass., *Hilliardiella* H. Rob., *Oocephala* H. Rob., *Vernonella* Sond., *Orbivestus* H. Rob. and *Parapolydora* H. Rob. Also present in southern Africa, but with species not listed by Harvey and Sonder (1894) are *Polydora* Fenzl, *Bothriocline* Oliv. ex Benth. in Hook., *Cyanthillium* Blume, and *Erlangea* Sch. Bip. Most of the taxa involved in the study are found in southern Africa, but a few species are mentioned that are not known from southern Africa but occur in Angola, Mozambique or Zimbabwe, and many subspecific taxa mentioned in synonymies are based on type specimens that were not collected in or near southern Africa.

Some of the proper generic dispositions were established in various papers such as Robinson and Kahn (1986) dealing with *Distephanus* Cass. plus one species of *Gymnanthemum*, and by Robinson (1999a) dealing with many genera of the paleotropical region. Some of the genera have been discussed in individual papers, *Parapolydora* in Robinson (2005) and Robinson and Funk (2011), *Orbivestus* in Robinson (2009) and *Vernonella* in Robinson and Skvarla (2010a). The present paper disposes of all but two of the southern African species now known that were previously placed in the genus *Vernonia*.

## Material and methods

In the following treatment, each genus is described or redescribed with general habit, types of vegetative trichomes, head structure, achene setulae and other trichomes, idioblasts and raphids, pappus form, and pollen form. Secondary metabolite chemistry is indicated based on data from two rather extensive summaries of constituents in the tribe by Bohlmann and Jakupovic (1990) and Herz (1996).

Figures are numbered in the order of the taxonomic treatment. Among the characteristics used in the classification, some special comments are in order.

### Trichomes

The trichomes of the African Vernonieae may be simple or with transversely affixed cap-cells as indicated below in the key and descriptions (Robinson 1999a). There are no stellate or goblet-formed trichomes such as those found in the American Vernonieae of the subtribe Piptocarphinae (Robinson 1999b).

### Pollen variation

The pollen is complicated, showing variation from nearly non-lophate to sublophate or lophate with or without colpi (Figs 3, 4, 8, 10, 14, 16, 18, 19, 21, 22, 24, 25; see Appendix A for definitions). In addition, grains show various degrees of loss of the perforated tectum. The structure of the muri and distribution of columellae also varies, and there is a variation from the usual tricolporate or non-colpate triporate conditions to a previously unknown form with pores greater in number and non-equatorial in distribution known as pantoporate.

Regarding the lophate condition, in reality, none of the grains in the Vernonieae has completely evenly spaced spines or columellae, and thus none are completely non-lophate. *Lophate*, in the Vernonieae, is defined as: pollen having the perforated tectum non-continuous in the intercolpar areas (Fig. 3A-E, 4A, B, D-F). In what is called lophate in many Lactuceae or Arctoteae taxa, the perforated tectum is always continuous in all non-colpar areas. This same structure in the Vernonieae is called *sublophate*: having the perforated tectum continuous between the colpi, supported by massive columellae or baculae, with spines being almost always present over the baculae. These sublophate forms differ from truly *non-lophate* forms in having the arrangement of the spines somewhat to distinctly uneven, leaving incipient lacunae. Examples of this sublophate morphology are seen in southern African Vernonieae in *Distephanus angustifolius* (O. Hoffm.) H. Rob. & B. Kahn (Fig. 4G–I), *Gymnanthemum capense* (A. Spreng.) J.C. Manning & N. Swelankomo (Fig. 10A–C), *Hilliardiella capensis* (Houtt.) H. Rob., Skvarla & V.A. Funk (Fig. 14A–F), *Orbivestus cinerascens* (Sch. Bip. in Schweinf.) H. Rob. (Fig. 19A–C), and *Pseudopegolettia* (Fig. 24A–F). In *Gymnanthemum* (Fig. 10A–C), the incipient lacunae in the intercolpi are in a pattern of 1-2-2-1, a pattern like that seen in the fully developed lacunae of lophate colpate grains in *Linzia* and *Baccharoides*. The pollen of *Gymnanthemum* might be referred to as lophate in other tribes. Its grains are totally radially symmetrical. The baculae in all of these sublophate forms are freestanding and are firmly attached to the footlayer. All of these above mentioned grains also seem to grade into forms of lophate grains that are highly perforate and spinose. These grains are referred to here as sub-echinolophate (Fig. 4G-I, 10A-C, 14A-F, 19A-C, 24A-F).

In addition to the sublophate pollen types described above, there are many variations of lophate grains, grains with ‘perforated tectum lacking’ to various degrees in the lacunae or even on the muri. Of these lophate types, one variant, represented by *Baccharoides* (Fig. 3 A–I), *Bothriocline* (Fig. 4 A–C) and *Cyanthillium* (Fig. 4 D–F), has prominent highly perforate lophae (muri) with sharply projecting spines. The lophae and supporting thickened columellae or baculae are similar to those in the mentioned above: *Distephanus*, *Gymnanthemum*, *Hilliardiella*, *Orbivestus*, and *Pseudopegolettia*. These grains are termed *echinolophate* (Fig. 3A-E). A variant of the echinolophate types is seen in *Linzia* where the surface of the lophae is highly perforate and supported by massive columellae or baculae, but is without spines and is classified as *psilolophate* (Fig. 16A, B, D).

### Pollen and Subtribal Classification

The most systematically important subdivision among the lophate types of pollen are the strongly colporate types as seen in *Baccharoides* and *Linzia* of the subtribe Linziinae (Figs 3 A–H, 16 A–E) as distinguished from the non-colpate porate forms of the genera of the subtribes Erlangeinae or Centrapalinae (see below). The Linziinae genera have either a distinct polar lacuna or an orderly arrangement of lacunae at the polar junctures of the three colpi. The patterns of distribution of these characters suggest that the sublophate pollen of all members of the Vernonieae may be reversion types from various lophate types. It is thus notable that the lophate types and sublophate types of the Linziinae and Gymnantheminae all have radially symmetrical organization with regular arrangement of lacunae or incipient lacunae in both lophate and sublophate forms. The non-colpate lophate pollens of the Centrapalinae and Erlangeinae treated below also have sublophate types that are colpate but have smaller and more irregularly arranged incipient lacunae.

*Linzia* has baculae that are connected to each other at their bases and have fewer and weaker attachments to the footlayer. This latter condition approximates what is referred to as the rhizomate or two-layered lophae in some members of the Erlangeinae and Centrapalinae treated below, and what is common in the New World subtribe Lepidaploinae (Keeley and Robinson 2009) previously placed in the Vernoniinae (Robinson 1999b).

A different pattern is seen in the many members of the subtribes Erlangeinae and Centrapalinae, where in both the sublophate tricolporate and lophate triporate forms, the incipient lacunae of the sublophate forms and the lacunae of the lophate forms are as mentioned above, smaller and in no regular pattern. The rather irregular disposition of lacunae is especially noticeable at the poles of the grains. For these latter forms, two other terms must be added, tricolporate sublophate (Fig. 10A–C) and triporate lophate (Fig. 4A, B, D–F).

The triporate grains in the Erlangeinae and Centrapalinae have subtypes. *Cyanthillium* (Fig. 4 D–F) has baculae only at the intersections of the muri or lophae, and *Bothriocline* (Fig. 4 A–C), *Erlangea* (Fig. 8 A–C.), and *Namibithamnus* (Fig. 16 F–H) have baculae that intrude upon the submural space (the space under the lophae) but tend to leave an ogee-shaped gap in the middle (Fig. 16 F–G). *Bothriocline* is distinct in the triplet of slightly connected lacunae that represent a minimal incipient colpus (Fig. 4 A–C). *Namibithamnus*, *Oocephala* and *Polydora* have pollen with greatly reduced perforated tectum and may be completely non-microporate. *Oocephala* (Fig. 18 B–E) and *Polydora* (Fig. 22 A–I) have many evenly spaced baculae or columellae subtending each of the muri or lophae. The baculae of these latter two genera are subtended by a continuous “rhizomate” structure that is itself only weakly attached to the footlayer (Figs 18 F, 22 C). This structure of the lophae could be described as having two equally thick layers separated by numerous very short evenly spaced columellae. In all of these listed lophate non-colpate genera, the columella or baculae under the lophae or muri tend to be in a single row. *Vernoniastrum* differs by the irregularly aligned or double-rowed columellae under the muri (Fig. 25 I).

The genera *Oocephala* and *Polydora* have the most distinctive pollen of all genera presently known in the Asteraceae. They have a 5–8-porate condition with pores distributed non-equatorially in noncontiguous (Fig. 18) or sometimes contiguous lacunae (Figs 18A, B, 22B). These grains are not radially symmetrical but essentially spherically symmetrical or totally asymmetrical, termed pantoporate (Robinson and Skvarla 2014). This differs from the 6-pores in three equatorial pairs found in the southeast Asian genus *Camchaya* Gagnep. in Lecomte (Bunwong and Chantaranothai 2008, Robinson and Skvarla 2010b).

The genera discussed in the section below fall into a number of subtribes. Some genera, from the more basal subtribes (based on DNA studies by Keeley et al. 2007), i.e. Distephaninae (*Distephanus*), Linziinae (*Baccharoides*, *Linzia*), and Gymnantheminae (*Gymnanthemum*), all have tricolporate pollen grains that are either lophate or sublophate.

### Pollen and Chemistry

The Distephaninae, Linziinae, and Gymnantheminae, have tricolporate sublophate or lophate forms of pollen and contain elemanolide sesquiterpene lactones as secondary metabolites. In contrast, two of the genera are in the more highly nested subtribe Centrapalinae (*Hilliardiella*, *Parapolydora*) and have weakly sublophate, tricolporate pollen and glaucolide/hirsutanolide sesquiterpenes. According to results from DNA studies combined with some obvious relationships based on pollen, two other genera with lophate, pantoporate pollen also belong to the Centrapalinae (*Oocephala* and *Polydora*).

Most of the remaining genera in the study, on the basis of DNA, structural or other evidence are presently placed in the subtribe Erlangeinae (*Bothriocline*, *Cyanthillium*, *Erlangea*, *Ethulia*, *Namibithamnus*, *Orbivestus*, *Pseudopegolettia*, and *Vernoniastrum*) which includes all the genera that contain the non-sesquiterpenoid 5-alkylcoumarin secondary metabolites.

The genus *Vernonella* has been placed in the subtribe Linziinae with some question by Robinson and Skvarla (2010a).

## Results

### Disposition of the genera of southern African Vernonieae into subtribes

Subtribe Centrapalinae: *Hilliardiella*, *Oocephala*, *Polydora*, *Parapolydora*

Subtribe Distephaninae: *Distephanus*

Subtribe Erlangeinae: *Bothriocline*, *Cyanthillium*, *Erlangea*, *Ethulia*, *Namibithamnus*, *Orbivestus*, *Pseudopegolettia*, *Vernoniastrum*

Subtribe Gymnantheminae: *Gymnanthemum*

Subtribe Linziinae: *Baccharoides*, *Linzia*, *Vernonella*-placement uncertain

Subtribe Unknown: *Vernonia potamophila*

The presently recognized genera of the Vernonieae in southern Africa can be distinguished by the following key.

### Key to the genera of the Vernonieae in southern Africa

**Table:** 

1	Leaf venation triplinervate; flowers usually yellow or orange, sometimes purple or white (Subtribe Distephaninae)	***Distephanus***
–	Leaf venation pinnate or without evident secondary veins; flowers usually purple or blue, sometimes white, never yellow or orange	**2**
2	Plants woody, shrubs or small trees; outer surfaces of involucral bracts with broad smooth shields, without evident strong midveins or keels (Subtribe Gymnantheminae)	***Gymnanthemum***
–	Plants herbaceous or small shrublets; outer surfaces of involucral bracts narrow or with midveins or keels	**3**
3	Involucral bracts usually with rounded tips and with the scarious margin continuous across tip	***Vernonella***
–	Involucral bracts with acute or awned tips; without continuous scarious margins across tips	**4**
4	Plants with either involucral bracts with spicules on margins or with broad flattened pappus bristles; pollen lophate and tricolporate, sometimes not echinate (subtribe Linziinae)	**5**
–	Plants with neither involucral bracts with spicules on margins nor with broad flattened pappus bristles; pollen nearly nonlophate or sublophate and echinate or triporate, not lophate combined with tricolporate	**6**
5	Involucral bracts without spicules along margins; basal tubes of corollas slender with expanded throat longer than the lobes; pappus bristles broad and flattened outside; pollen with polar lacunae, without spurs projecting into colpi	***Baccharoides***
–	Involucral bracts with spicules along lateral margins; corollas funnel-form with lobes longer than throat; pappus bristles capillary, not flattened outside; pollen without polar lacunae, with spurs projecting into colpi above and below pores	***Linzia***
6	Setulae of achenes deeply divided, sometimes with single cell from near base; hairs of stems simple; pollen tricolporate, non-lophate (typical element of subtribe Centrapalinae)	***Parapolydora***
–	Setulae of achenes, when present, with pairs of cells not or scarcely divided at tips; hairs of stems simple, T-shaped or L-shaped; pollen triporate or polyporate without colpi or non-lophate and tricolporate (some Centrapalinae and members of subtribe Erlangeinae)	**7**
7	Pappus bristles elongate and subplumose	***Oocephala***
–	Pappus bristles absent, short, scabrid or barbellate	**8**
8	Involucral bracts ca. 80 in ca. 6 series; stems with asymmetrical L-shaped hairs, with cap-cell mounted near one end; pollen pantoporate	***Polydora***
–	Involucral bracts less than 50 in less than 5 series; stems with variously shaped hairs; pollen triporate	**9**
9	Pollen sublophate, without distinct polar lacunae	**10**
–	Pollen lophate and triporate, with irregular cluster of polar lacunae	**14**
10	Pappus totally lacking or present as cylindrical collar	***Ethulia***
–	Pappus with capillary bristles	**11**
11	Heads few or solitary at tips of long branches or peduncles; stems with short often asymmetrically capped hairs	***Pseudopegolettia***
–	Heads clustered at tips of branches; stems usually with T-shaped hairs	**12**
12	Stems with yellowish-brown-velutinous pubescence (unplaced)	***Vernonia potamophila***
–	Stems with sericeous to hirsute pale pubescence	**13**
13	Inflorescences with heads in corymbiform cymes; stems, involucres and corollas with symmetrically T-shaped hairs	***Hilliardiella***
–	Inflorescence with heads in seriate cymes; corollas without T-shaped hairs	***Orbivestus***
14	Pappus bristles much shorter than corollas or lacking, easily deciduous; achenes short and broad, narrowed greatly apically to the narrow insertion of the corolla	**15**
–	Pappus bristles about as long as corolla, rather persistent; achenes not greatly narrowed distally to insertion of corolla	**16**
15	Hairs of stems often T-shaped with long arms; leaves alternate, opposite or whorled; corolla lobes without long hairs at apex; achenes with few raphids or thick sclerified layer inside of wall; pollen with 2 or 3 lacunae with incomplete muri adjacent to pores	***Bothriocline***
–	Hairs of stems and branches simple with short basal cells and long flexuous terminal cell; leaves alternate; corolla lobes with long hairs at apex; achenes without thick sclerified layer inside, with well-developed layer of dense subquadrate cells containing subquadrate or short-oblong raphids; pollen strictly triporate	***Erlangea***
16	Hairs of stems simple or asymmetrical; achenes with numerous idioblasts densely clustered in transverse bands	***Vernoniastrum***
–	Hairs of stems symmetrically T-shaped; achenes with idioblasts not in distinct transverse bands	**17**
17	Short-lived herbs; hairs with long armed cap cells, forming hirsute or pilose indument	***Cyanthillium***
–	Small subshrubs; hairs of stems and bracts with small or elongate cap-cells, forming dense tomentellous or sericeous cover	***Namibithamnus***

### Taxonomy

#### Baccharoides

Taxon classificationPlantaeAsteralesAsteraceae

Moench, 1794

**Figure 1.**
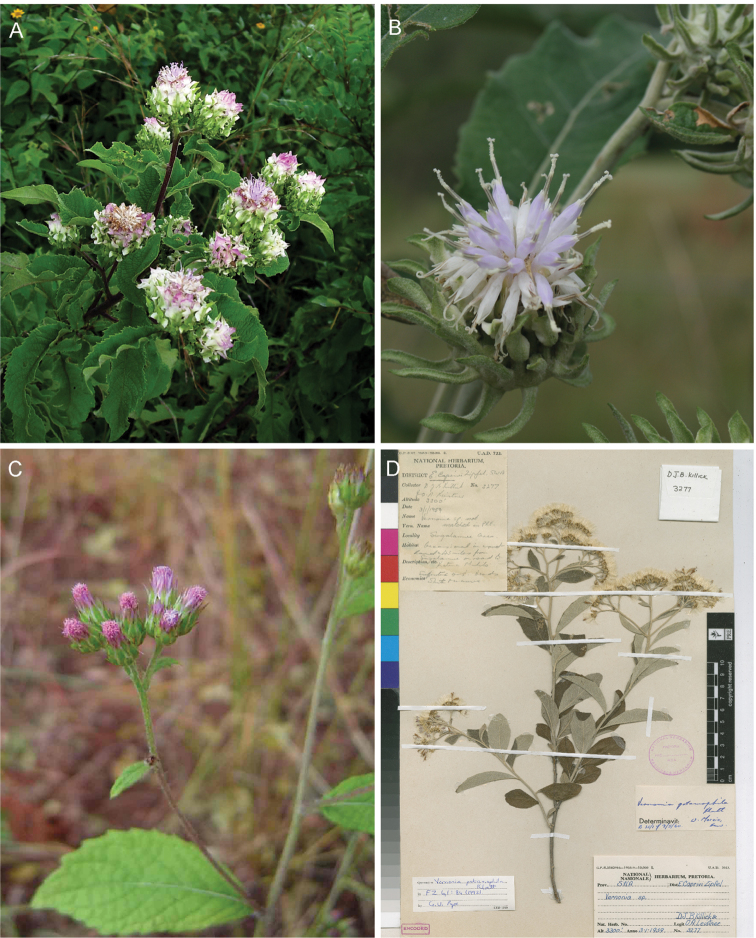
Photographs of *Baccharoides*, *Bothriocline*, and *Vernonia potamophila*. *Baccharoides adoensis* (Sch. Bip. ex Walp.) H. Rob. **A** Habit **B** Close up of flowering head: note that the corollas are narrowed near the apex and have an lengthened throat that is much longer than the short lobes and the involucral bracts have a differentiated margin that is often pale or reddish; *Bothriocline laxa* N.E. Br. **C** Immature heads; *Vernonia potamophila* Klatt. **D** Image of herbarium specimen (PRE). See Appendix C for citation details.

**Figure 2.**
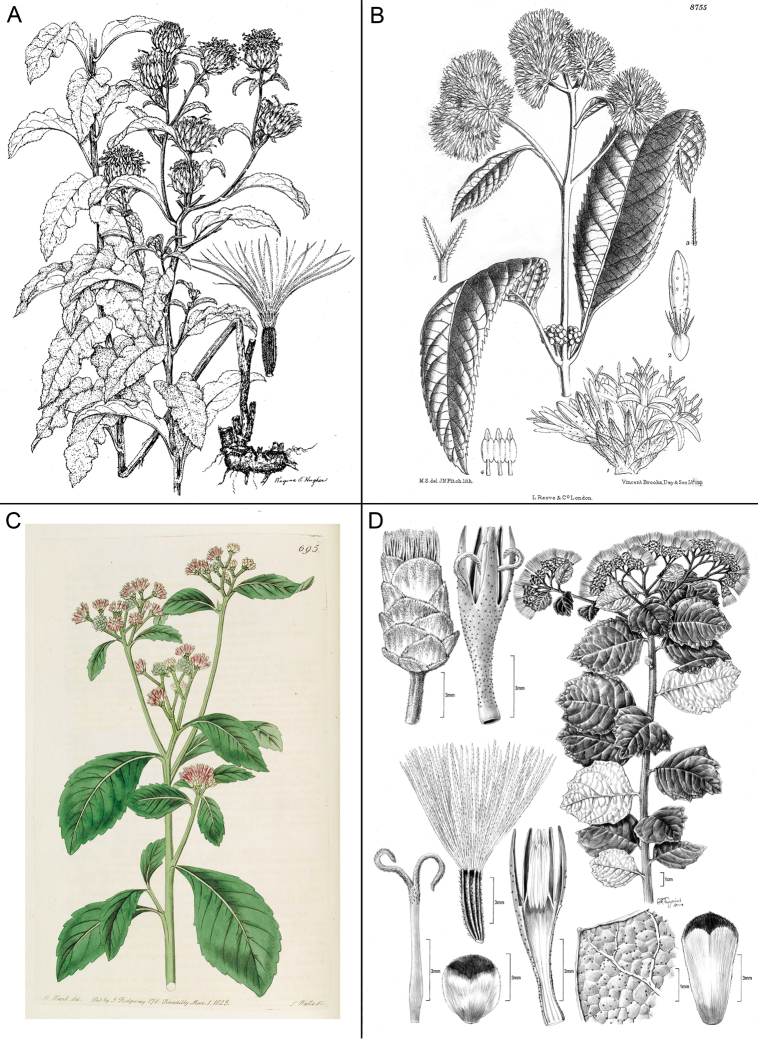
Illustrations: **A** *Baccharoides adoensis* (Sch. Bip. ex Walp.) H. Rob. **B** *Bothriocline aggregata* Hutch., note: this taxon is not found in southern Africa **C** *Ethulia conyzoides* L.f., note: lack of pappus; and **D** *Gymnanthemum koekemoerae* H. Rob. & V. Funk, note: broad involucral bracts without a high midrib. See Appendix C for citation details.

**Figure 3.**
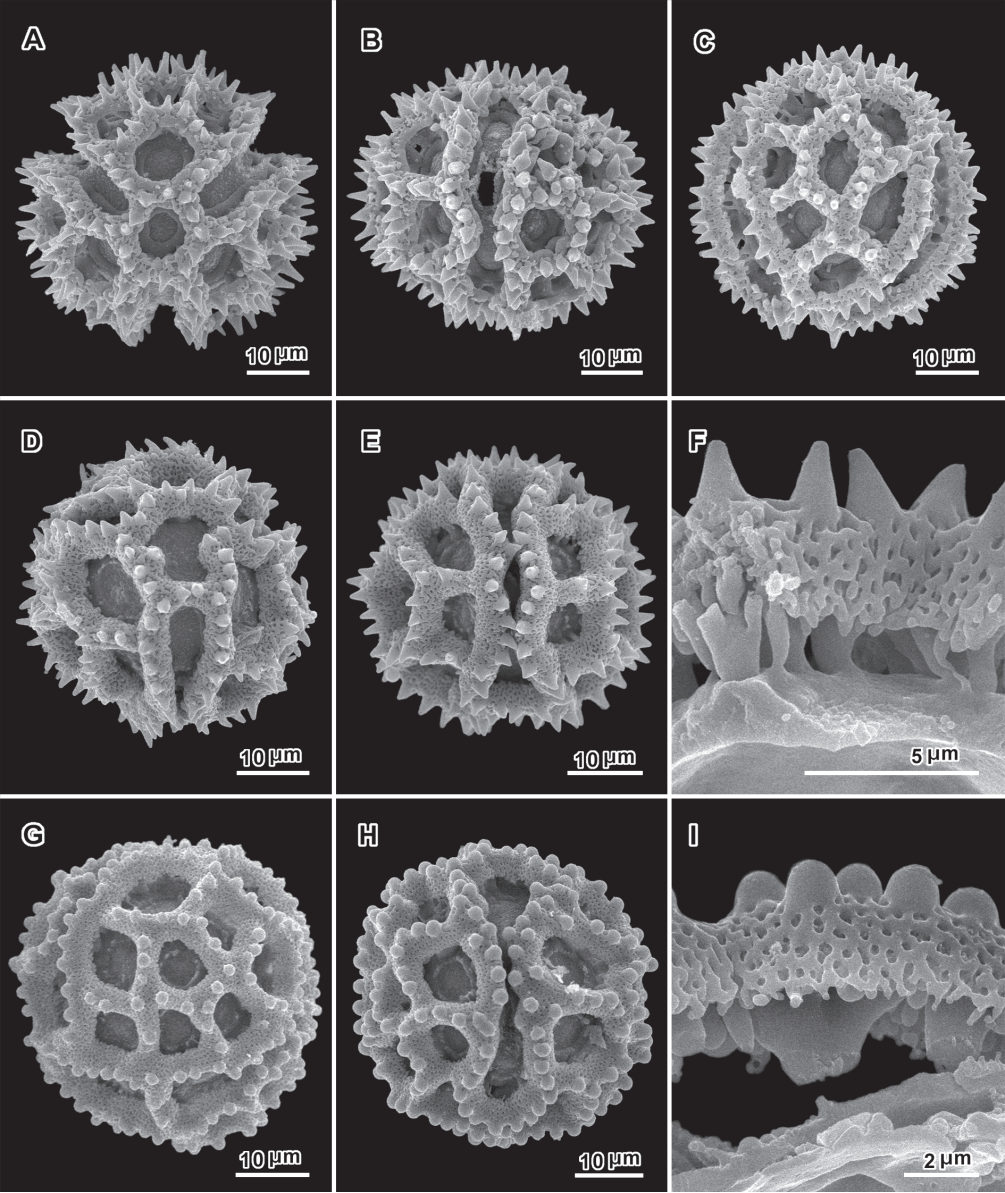
Scanning electron electron micrographs from three collections of acetolyzed echinolophate *Baccharoides* pollen showing variations in spine shape from acute to markedly blunt. **A–H.** *Baccharoides anthelmintica* (L.) Moench. **A** polar view **B** Equatorial view **C** Lateral view **D** Near polar view **E** Equatorial view **F** Fractured grain **G** Lateral view **H** Equatorial view **I** Fractured grain. (A–C, USDA P.I. *283729*; D–F, Cooray 70031701R; G–I, *Koelz 7469*). [Views from Robinson and Skvarla (2010); Figure 1 B = original Figure 1G; Figure 1C = original Figure 1H; and Figure 1H = original Figure 1J.]

Figures 1 A, B ; 2 A ; 3 A–I

Baccharoides Moench, Methodus 328 (1794). – Type: *Conyza anthelmintica* L.
Ascaricida Cass., Dict. Sci. Nat. 3, suppl. 38 (1817), nom. superfl. – Type: *Conyza anthelmintica* L.
Candidea Tenore, Atti Reale Accad. Sci. Sez. Soc. Reale Borbon 4 (CI. Botan.): 104, t. 1, 2 (1839). – Type: *Candidea senegalensis* Tenore.
Vernonia subsect. Stengelia Sch. Bip. ex Walp., Repert. Bot. Syst. 2: 946 (1843). – Type: *Vernonia adoensis* Sch. Bip. ex Walp.
Stengelia Steetz in Peters, Reise Mossamb., Bot. 360. 1864. – Type: *Vernonia schimperi* DC.
Vernonia sect. Stengelia (Sch. Bip. ex Walp.) Benth. in Benth. & Hook.f., Gen. Pl. 2: 127 (1873).

##### Resources.

Treatment by Isawumi et al. (1996).

##### Descriptions.

Annual or perennial herbs, suffruticose; stems erect or reclining; hairs short-stalked with an erect, elongate apical cell. Leaves alternate, narrowly petiolate; blades chartaceous, ovate to elliptic, serrate, secondary veins pinnate, ascending at 45° angles or more. Inflorescence with single lateral or terminal head or heads in corymbiform groups; peduncles usually solid, sometimes fistulose. Heads with involucres broadly campanulate or hemispherical; bracts 25–100 in 4–8 series, mostly gradate but with outer bracts sometimes elongate and foliiform, tips of bracts appendaged, white or colored; receptacles epaleate. Florets 25–100 in a head; corollas reddish or lavender to white, with long slender basal tube, limb abruptly expanded at base, cylindrical, with lobes about as long as throat, erect, with various hairs and glands outside, inside with cells elongate, transversely striate; anther thecae spurred with small tails; endothecial cells with nodular thickenings on tranverse walls; apical appendages oblong-ovate, rounded or acute at tips, glabrous; nectary elongate, cylindrical; style base without node; sweeping hairs acicular. Achenes cylindrical or turbinate, 8–20-costate, glabrous or with setulae distinctly cleft or with glands or idioblasts, carpopodium annuliform, large to obsolete, with thickened porose walls, raphids in ovules elongate, with rhomboid tips; pappus pluriseriate, persistent or caducous, inner capillary, flattened, barbellate on margins, sometimes shortly connate at base, sometimes with outer row of small scales. Chromosome number x = 10 (Jones 1970, Mathew and Mathew 1983)

Pollen. 43.5–72.0 μm diam. (Isawumi et al. 1996); tricolporate, echinolophate; lacunae regularly disposed, one at each pole, 2 across intercolpus; tectum restricted to muri, with distinct microperforations; stout baculae under muri firmly attached to footlayer (Fig. 3A–I).

Most notable secondary metabolites, sesquiterpene elemanolides (Bohlmann and Jakupovic 1990, as *Vernonia anthelmintica* (L.) Willd., *Vernonia hymenolepis* A. Rich., *Vernonia lasiopus* O. Hoffm.), eudesmanolide (Bohlmann and Jakupovi 1990, as *Vernonia adoensis* Sch. Bip. ex Walp.).

#### Key to the species of *Baccharoides*

**Table:** 

1	Leaf blades sessile or subsessile	***Baccharoides benguelensis***
–	Leaves distinctly petiolate	**2**
2	Branching perennial herbs from large root crown; fusiform tubers often present; peduncule not enlarged or fistulose distally	***Baccharoides adoensis***
–	Annual herbs; without tubers; peduncles often somewhat enlarged and fistulose distally	***Baccharoides anthelmintica***

#### Baccharoides adoensis

Taxon classificationPlantaeAsteralesAsteraceae

(Sch. Bip. ex Walp.) H. Rob., 1990

Vernonia adoensis Sch. Bip. ex Walp., Repert. Bot. Syst. 2: 946. 1843.
Stengelia adoensis Sch. Bip. ex Hochst., Flora 24: Intelligenzbl. 1841: 1(2): 26. 1841, nom. nud.
Vernonia kotschyana Sch. Bip. ex Walp., Repert. Bot. Syst. 2: 947. 1843.
Vernonia macrocephala A. Rich., Tent. Fl. Abyss. 377. 1847, nom. illeg., non Less.
Ascaricida adoensis (Sch. Bip. ex Walp.) Steetz in Peters, Reise Mossamb. 358. 1864.
Ascaricida mossambiquensis Steetz in Peters, Reise Mossamb 358. 1864.
Ascaricida richardi Steetz in Peters, Reise Mossamb. 358. 1864.
Vernonia grantii Oliv., Trans. Linn, Soc. London 29: 92. 1873.
Vernonia polymorpha var. adoensis (Sch. Bip. ex Walp.) Vatke, Linnaea 39: 476. 1875.
Vernonia polymorpha var. accedens Vatke, Linnaea 39: 477. 1875.
Vernonia polymorpha var. ambigua Vatke, Linnaea 39: 477. 1875.
Vernonia tigrensis Oliv. & Hiern in Oliv., Fl. Trop. Africa 3: 290. 1877.
Vernonia shirensis Oliv. & Hiern in Oliv., Fl. Trop. Africa 3: 291. 1877.
Vernonia mossambiquensis (Steetz) Oliv. & Heirn in Oliv., Fl. Trop. Afr. 3: 292. 1877, non *Vernonia mossambicensis* Busc. & Muschler 1913.
Vernonia whyteana Britten, Trans. Linn. Soc., London, ser. 2, 4: 17. 1894.
Vernonia leptolepis Bak., Bull. Misc. Inf. Kew 1898: 147. 1898, nom. illeg., non O. Hoffm. 1895.
Vernonia woodii O. Hoffm. in Engl., Bot. Jahrb. 38: 198. 1906.
Vernonia integra S. Moore, J. Bot. 46: 39. 1908.
Vernonia bequartii De Wild., Feddes Repert. 13: 206. 1914.
Vernonia integra S. Moore, J. Bot. 46: 39. 1918.
Candidea stenostegia Stapf, Bot. Mag. 149: t. 8981. 1923.
Vernonia latisquama Mattf., Bot. Jahrb. Syst. 59: Beibl. 133: 5. 1924.
Vernonia fulviseta S. Moore, J. Linn. Soc. Bot. 47: 266. 1925-27.
Vernonia stenostegia (Stapf) Hutch. & Dalz., Fl. W. Trop. Africa 2: 164. 164, in key 166. 1931.
Vernonia adoensis var. mossambiquensis (Steetz) G.V. Pope, Kew Bull. 43(2): 284. 1988.
Vernonia adoensis var. kotschyana (Sch. Bip. ex Walp.) G.V. Pope, Kew Bull. 43(2): 285. 1988.
Baccharoides adoensis (Sch. Bip. ex Walp.) H. Rob., Proc. Biol. Soc. Washington 103(1): 250 1990.
Baccharoides adoensis (Sch. Bip. ex Walp.) H. Rob. var. kotschyana (Sch. Bip. ex Walp.) Isawumi, El-Ghazaly & B. Nord., Grana 35. 219. 1996.
Baccharoides adoensis (Sch. Bip. ex Walp.) H. Rob. var. mossambiquensis (Steetz) Isawumi, El-Ghazaly & B. Nord., Grana 35. 219. 1996.

##### Distribution.

Ivory Coast, Ethiopia, Malawi, Mozambique, Zimbabwe, South Africa.

#### Baccharoides anthelmintica

Taxon classificationPlantaeAsteralesAsteraceae

(L.) Moench, 1794.

Conyza anthelmintica L., Sp. Pl. ed 2, 1207. 1763.
Baccharoides anthelmintica (L.) Moench, Method. 578., 1794.
Vernonia anthelmintica (L.) Willd., Sp. Pl. 3: 1634. 1803.
Vernonia stenolepis Oliv., Trans Linn. Soc. ser 2, 2: 337. 1887.
Dolosanthus sylvaticus Klatt, Bull. Herb. Boiss. 4: 473, t. 5. 1896.
Centratherum anthelminticum (L.) Gamble, Fl. Pres. Madras 2: 667. 1921.

##### Distribution.

Congo, Kenya, Tanzania, Uganda, Malawi, Zambia, Zimbabwe, Botswana, Namibia, Sri Lanka, Nepal, Pakistan, India, China.

#### Baccharoides benguellensis

Taxon classificationPlantaeAsteralesAsteraceae

(Hiern) H. Rob., Skvarla & V.A. Funk comb. nov.

urn:lsid:ipni.org:names:77152894-1

Vernonia benguellensis Hiern, Cat. Afr. Pl. 1: 536. 1898.
Vernonia limosa O. Hoffm. in Warburg, Kunene-Sambesi Exped. 400. 1903.

##### Distribution.

Angola, also cited from SW Africa, but that locality probably not intended in the restricted sense.

##### Note.

The species is known from photographs of types and from descriptions deposited at US by C.E. Smith. The type photographs – as well as Fig. 1 B (for corollas) – clearly show the corolla form and flattened pappus bristles of *Baccharoides*, and the species is not accounted for elsewhere. The type specimen of *Vernonia benguelensis* is collected in Angola, ad lacum de Ivantola, Feb. 1860, *Welwitsch 3276b* (BM, photo seen). The lectotype of *Vernonia limosa* is cited as Südwest Afrika, am Longa unterh. Chijija, Jan. 1900, *Baum 624* (BM, photo seen; Smith 1917). This locality is situated in Angola (Figueiredo et al. 2009).

#### Bothriocline

Taxon classificationPlantaeAsteralesAsteraceae

Oliv. ex Benth.

**Figure 4.**
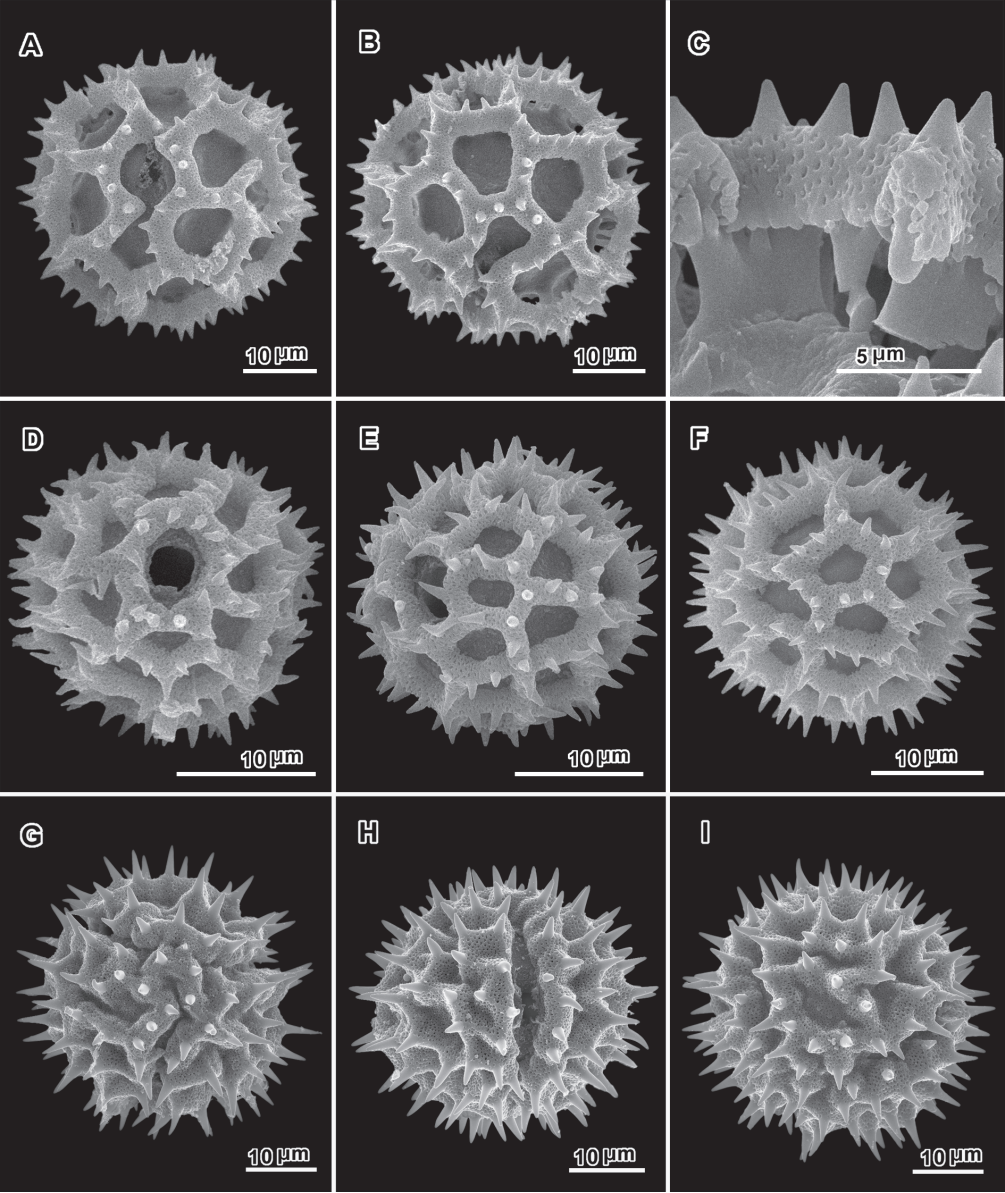
Scanning electron micrographs of acetolyzed pollen of echinolophate *Bothriocline* and *Cyanthillium* and sublophate-lophate *Distephanus*. **A–C** *Bothriocline schimperi* Oliv. & Hiern ex Benth. **A** Equatorial view, note incipient colpus of 3 connected lacunae centered on pore **B** Near polar view **C** Fractured grain **D–F** *Cyanthillium cinereum* (L.) H. Rob. **D** Equatorial view **E** Lateral view with apertures on sides **F** Lateral view **G–I** *Distephanus angustifolia* (DC.) **H** Rob. & B. Kahn. **G** Polar view **H** Equatorial view **I** Lateral view. (A-C, *F. Meyer 8159*; D-F, *Evans 344*; G-I, *Sidley 2211*).

Figures 1C ; 2B ; 4A–C

Bothriocline Hooker’s Icon. Pl. 12: 30, t. 1133. 1873. – Type: *Bothriocline schimperi* Oliv. & Hiern ex Benth.
Volkensia O. Hoffm., Bot. Jahrb. Syst. 20: 219. 1894; Engl. & Prantl, Natürl. Pflanzenfam. iv. 5: 387. 1893. – Type: *Volkensia argentea* O. Hoffm.

##### Resources.

Many species are keyed in Jeffrey’s (1988) treatment of Vernonieae in East Africa and in Wild and Pope (1977), Wild (1978a, 1978b).

##### Descriptions.

Perennial herbs (up to 1 m) to subshrubs, branching sparse, stems erect with a solid pith and long-armed T-shaped hairs with short 2-celled stalks. Leaves alternate, opposite or whorled, sessile to short petiolate, blade narrow to ovate or elliptical, pinnately veined, often paler or tomentose to sericeous below. Inflorescence laxly to densely corymbiform or thyrsiform cymes; heads pedunculate. Involucres campanulate, bracts ca. 50–60, gradate in 3–4 series, cuspidate at apex, with distinct pale or reddish lateral margins, nearly glabrous to pilosulous outside; receptacle convex, epaleaceous, with glabrous reticulum. Florets 3–100 or more in a head; corollas purplish, funnelform, basal tube slender with small stipitate glands, throat shorter than 1 mm, lobes, linear-lanceolate, with glandular dots and often with stiff subapical hairs; anther thecae blunt at base with few sterile cells; apical appendages ovate-oblong, with thin cell walls; style base with minimal annuliform node; sweeping hairs acicular, mostly restricted to branches. Achenes prismatic, short and broad with 3–6(–9) ribs, setuliferous with sparse short setulae scarcely split at tips, often densely covered with idioblasts and with scattered subquadrate raphids. Pappus of few or no short easily deciduous bristles narrowed at base, without obvious shorter ourter series or outer pappus a rim or collar. Chromosome number n = 9, 10, 18–20 (Jones 1979, 1982). Pollen grains ca. 47 μm in diam, lophate to rarely sublophate, finely echinate, pores in triplet of connected colpar lacunae, perforated tectum usually restricted to muri (Fig. 4 A–C).

Notable secondary metabolites include 5-alkylcoumarins and sesquiterpene glaucolides/hirsutanolides [Bohlmann and Jakupovic 1990, as *Bothriocline laxa* N.E. Br., *Bothriocline longipes* (Oliv. & Hiern) N.E. Br.], and *Volkesia ripensis* Hutch. and 5-alkylcoumarins (Bohlmann and Jakupovic 1990, as *Erlangea fusca* S. Moore, *Erlangea rogersii* S. Moore).

#### Bothriocline laxa

Taxon classificationPlantaeAsteralesAsteraceae

N.E. Br., 1894.

Bothriocline laxa N.E. Br., Bull. Misc. Inf. Kew 1894: 388. 1894.

##### Distribution.

Tanzania, Zambia, Malawi, Zimbabwe, Congo, Angola, South Africa (Transvaal).

#### Cyanthillium

Taxon classificationPlantaeAsteralesAsteraceae

Blume, 1826

**Figure 5.**
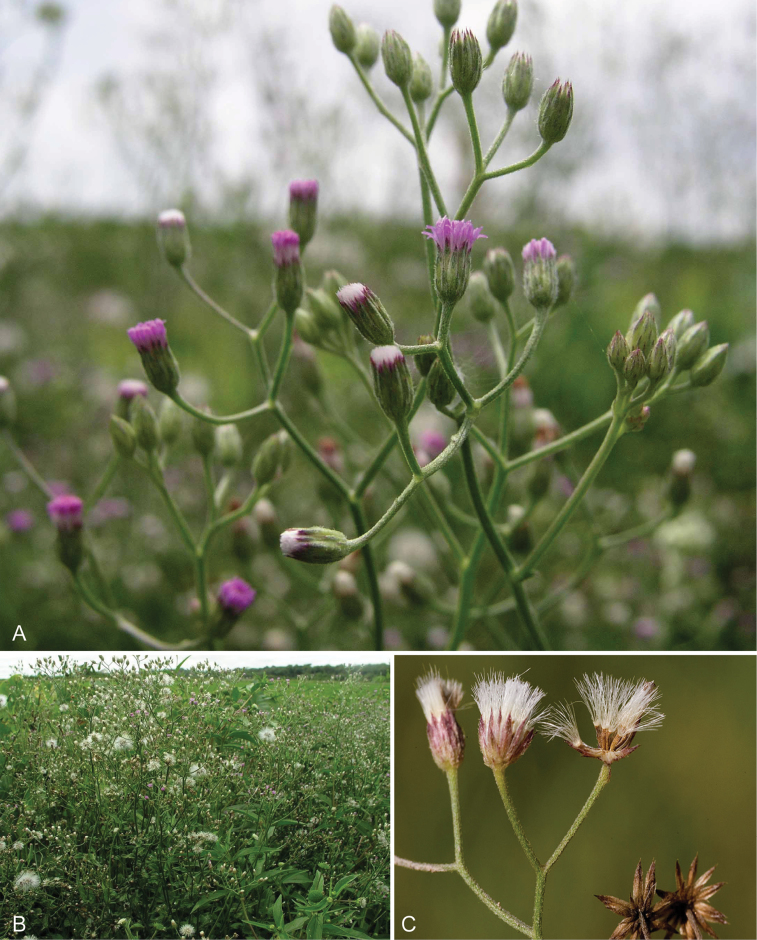
Photographs of *Cyanthillium cinereum* (L.) H. Rob: **A** Inflorescence, **B** habit, **C** Close up of heads in fruit. See Appendix C for citation details.

Figures 4 D–F ; 5 A–C

Cyanthillium Blume, Bidjr. 889. 1826. – Type: *Cyanthillium villosum* Blume
Isonema Cass., Bull. Soc. Philom. Paris 1817: 152. 1817, nom. illeg., non *Isonema* R. Br., 1810. – Type: *Isonema ovata* Cass.
Cyanopsis Blume ex DC., 5: 69. 1836, nom. illeg. superfl., non Cass. 1817.
Vernonia sect Tephrodes DC., Prodr. 5: 24. 1836. – Lectotype: *Conyza cinerea* Blume (Jones 1981a).
Claotrachelus Zoll. & Moritz ex Zoll., Natuur-Geneesk. Arch. Ned. Indie 2: 263, 565. 1845. – Type: *Claotrachelus rupestris* Zoll. & Moritz ex Zoll.
Seneciodes L. ex Post & O. Kuntze, Lex. Gen. Phan. 2: 515. 1903. – Type: *Conyza cinerea* L.
Triplotaxis Hutch., Bull. Misc. Inform. 1914: 355. 1914. – Lectotype: *Herderia stellulifera* Benth. in Hook. (Robinson 1990a).
Vernonia subsect. Tephrodes (DC.) S.B. Jones, Rhodora 83: 70. 1981.

##### Resources.

Traditionally treated as part of Vernonia.

##### Descriptions.

Annual or short-lived perennial herbs to 1 m tall; stems erect or spreading; hairs symetrically or asymetrically T-shaped with short stalk. Leaves alternate; petioles narrow; blades membranaceous, ovate to narrowly lanceolate. Inflorescences terminal, moderately densely to laxly branching, distinctly cymiform or with rather corymbiform branches, with minute bracteoles; peduncles rather short to elongate. Heads narrowly campanulate, involucral bracts ca. 30 in 3(–5) series, gradate, thinly chartaceous, green with pale or purplish margins, persistent, often with pilose to sericeous pubescence; receptacles epaleaceous. Florets 15–94 in a head; corollas bluish to lavender, funnelform with slender lower tubes, throat a third as long to nearly as long as lobes, lobes with simple hairs especially near tips; anthers without tails; apical appendages oblong-ovate, glabrous, with thin cell walls; style base with broad node; style branches with acicular sweeping hairs. Achenes 5-ribbed, or terete, setulae shortly cleft at tips, with idioblasts, sometimes with glands, raphids elongate; inner pappus of many long, sometimes rather fragile, slender-tipped capillary bristles, outer series of persistent squamellae, one species with callose ring. Chromosome number n = 9, 18, 20 (Turner and Lewis 1965, Mathew and Mathew 1976, Jones 1979).

Pollen ca. 30 μm in diameter (dry); triporate, echinolophate, ca. 21 lacunae rather irregularly disposed at poles and in intercolpi; perforated tectum restricted to ridges of muri, with distinct microperforations; spinules of muri short, shorter than width of mural ridge, pointed, without columellae under each murus; baculae single at junctures of muri and no baculae between junctures, each intersection of muri with stout columella that is firmly attached to footlayer (Fig. 4 D–F).

Notable secondary metabolites, 5-alkylcoumarins, sesquiterpene glaucolides, guanolides (Bohlamnn and Jakupovic 1990, as *Vernonia chinensis* Less., *Vernonia cinerea* Less.).

#### Key to the species of *Cyanthillium*

**Table:** 

1	Plants perennial, weakly frutescent, often scrambling	***Cyanthillium wollastonii***
–	Plants annual	**2**
2	Inner pappus absent or of few dissected scales; outer pappus forming a collar	***Cyanthillium stelluliferum***
–	Inner pappus of many bristles; outer pappus not forming a collar	**3**
3	Outer pappus of short oblong often rounded scales less than 0.2 mm long	***Cyanthillium vernonioides***
–	Outer pappus of narrow lanceolate scales 0.2 or more long	***Cyanthillium cinereum***

#### Cyanthillium cinereum

Taxon classificationPlantaeAsteralesAsteraceae

(L.) H. Rob., 1990

Conyza cinerea L. Sp. Pl. 862. 1753.
Vernonia cinerea (L.) Less., Linnaea 4: 291. 1829.
Vernonia lentii O. Hoffm. in Engl., Pflanzenw. Ost-Afr. C: 404. 1895.
Seneciodes cinerea (L.) Post & Kuntze, Lex. Gen. Plan. 2: 515. 1903.
Cyanthillium cinereum (L.) H. Rob., Proc. Biol. Soc. Wash. 103: 252. 1990

##### Distribution.

Widely introduced weed, pantropical.

#### Cyanthillium stelluliferum

Taxon classificationPlantaeAsteralesAsteraceae

(Benth.) H. Rob., 1990

Herderia stellulifera Benth. in Hook.f. & Benth., Niger Fl. 425. 1849.
Triplotaxis stellulifera (Benth.) Hutch., Bull. Misc. Inf. Kew 1914: 356. 1914.
Cyanthillium stelluliferum (Benth.) H. Rob., Proc. Biol Soc. Wash. 103(1): 252. 1990.

##### Distribution.

Tropical Africa south to Angola.

#### Cyanthillium vernonioides

Taxon classificationPlantaeAsteralesAsteraceae

(Muschl.) H. Rob., 1999

Erlangea vernonioides Muschl., Bot. Jahrb. Syst. 45: 62. 1911, non *Vernonia vernonioides* (A.Gray) Bacigalupo 1931.
Vernonia meiostephana C. Jeffrey, Kew Bull. 43: 225. 1988.
Cyanthillium vernonioides (Muschl.) H. Rob., Proc. Biol. Soc. Wash. 112(1): 229. 1999.

##### Distribution.

Tropical Africa from Congo, Uganda and Kenya south to Zambia, Zimbabwe and South Africa (Transvaal), Madagascar.

#### Cyanthillium wollastonii

Taxon classificationPlantaeAsteralesAsteraceae

(S. Moore) H. Rob., Skvarla & V.A. Funk comb. nov.

urn:lsid:ipni.org:names:77152895-1

Vernonia wollastonii S. Moore, Journ. Linn. Soc. 38: 257. 1908.
Vernonia gracilipes S. Moore, Journ. Linn. Soc. 40: 105. 1911.
Vernonia heterocarpa Chiov., Nuov. Giorn. Bot. Ital, n.s. 36: 365. 1929.
Vernonia transvaalensis Hutchinson, Botanist S. Afr. 347. 1946, in note.
Vernonia umbratica Oberm., J. S. Afr. Bot. 2: 164. 1936.

##### Distribution.

Abyssinia, Malawi, Sudan, Swaziland, Tanzania, South Africa (Transvaal), Uganda, Zimbabwe.

#### Distephanus

Taxon classificationPlantaeAsteralesAsteraceae

Cass., 1817

**Figure 6.**
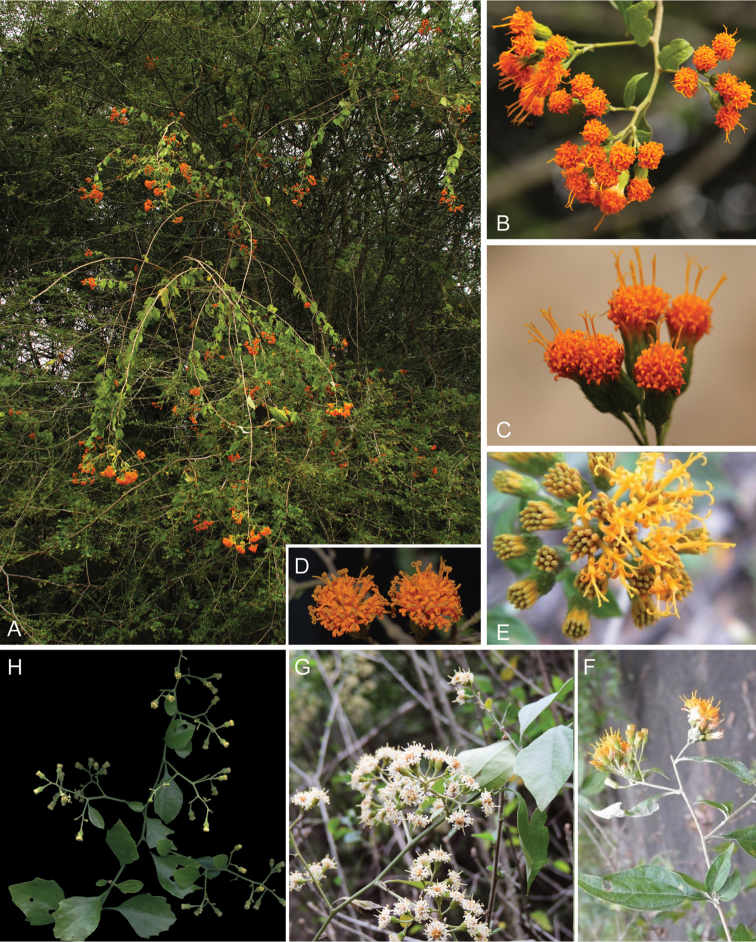
Photographs of *Distephanus*: **A–F** *Distephanus divaricatus* (Steetz) H. Rob. & B.Kahn; **G–H** *Distephanus anisochaetoides* (Sond.) H. Rob. & B.Kahn. Note the variable flower color in *Distephanus divaricatus*. Note: trinervate veination, lack of lavender corollas and presence of yellow and orange. See Appendix C for citation details.

Figures 4 G–I ; 6 A–H

Distephanus Cass. Bull. Soc. Philom. Paris 1817: 151. 1817.
Gongrothamnus Steetz in Peters, Reise Mossamb., Bot.: 336. 1864. – Type: *Gongrothamnus divaricatus* Steetz in Peters
Newtonia O. Hoffm. in Engler & Prantl, Natürl. Pflanzenfam. 4(5): 285. 1892, nom. illeg., non Baill. 1888. – Type: *Newtonia angolensis* O. Hoffm.
Antunesia O. Hoffm., Bolet. Soc. Brot. 10” 178. 1893 (“1892”), nom. nov. for *Newtonia*.

##### Resources.

For discussion and numerous transfers of species see treatment by Robinson and Kahn (1986). For a recent treatment of the genus in southern Africa see Swelankomo and Manning (2014).

##### Descriptions.

Shrubs or vines; hairs arachnoid, contorted or asymmetrically T-shaped. Leaves alternate; petioles short; blades ovate to rounded, often with truncate to subcordate bases, less often narrow with cuneate bases, margins usually entire or subentire, venation usually with stronger more ascending basal pair or strongly triplinervate, less often irregularly pinnate. Inflorescences terminal on stems or branches, with single heads or usually branching, corymbiform with minute bracts or thyrsoid with foliose bracts; peduncles usually short. Heads with campanulate involucres; bracts 21–24(–75) in 4–6(–7) gradate series, without appendaged tips; receptacles epaleaceous. Florets 10–16(–75) in a head; corollas usually yellow, purplish in a few continental African species; anther thecae with distinct broad often sclerified basal appendages; endothecial cells with simple, broad, non-contiguous, sclerified shields; apical appendages without glands; style base with large abruptly distinct node; style branches with obtuse sweeping hairs. Achenes cylindrical to prismatic, sometimes subtriquetrous or quadrangular, with 5–12 ribs, usually 10, setulae or glands present or absent, raphids elongate; carpopodium turbinate; pappus of many capillary bristles, outer series of squamellae. Chromosome numbers n = 9, 10, 15 (Jones 1982, Gill and Omoigui 1992).

Pollen: 30–36 μm in diameter (dry); tricolporate, sublophate to lophate; lophate forms with muri projecting as spurs into colpus, with echinate or with nearly psilate ridges; tectum continuous in intercolpi and at poles, or in pockets surrounded by ridges, with distinct perforations; with columellae under spines or with muri granular inside, without distinct baculae (Figs 4 G–I).

Notable secondary metabolites: sesquiterenes, elemanolides (Bohlmann and Jakupovic, as *Gongrothamnus aurantiaca* N.E. Br.), guaianolide (as *Gongrothamnus sublutea* Elliot., guaianolides (Bohlmann and Jakupovic 1990, as *Vernonia anisochaetoides* Sond., glaucolides/hirsutanolides (Bohlmann and Jakupovic 1990, as *Vernonia angulifolia* DC., *Vernonia tufrnellae* S. Moore).

#### Key to the species of *Distephanus*

**Table:** 

1	Involucral bracts oblong with obtuse or shortly acute tips	**2**
–	Involucral bracts lanceolate, narrowly acute	**3**
2	Leaf blades ovate, with marginal lobes; branches of inflorescence essentially straight; corollas purple or yellow	***Distephanus angulifolius***
–	Leaf blades rhomdoidal, cuneate proximally; inflorescence branches with strong zigzag pattern; corollas white	***Distephanus anisochaetoides***
3	Corollas purple or white	***Distephanus inhacensis***
–	Corollas yellow or orange	**4**
4	Stems and abaxial surfaces of leaves not tomentellous; leaf blades oblong or ovate-elliptical, often blunt at tip	***Distephanus angolensis***
–	Stems and abaxial surfaces of leaves with fine tomentellum; leaf blades ovate, broadest at or below proximal third	***Distephanus divaricatus***

#### Distephanus angolensis

Taxon classificationPlantaeAsteralesAsteraceae

(O. Hoffm.) H. Rob. & B. Kahn, 1986

Newtonia angolensis O. Hoffm., Natürl. Pflanzenfam. 4(5): 285. 1892.
Antunesia angolensis (O. Hoffm.) O. Hoffm., Bolet. Soc. Brot. 10: 178. 1893.
Gongrothamnus angolensis (O. Hoffm.) Hiern, Cat. Welw. Afr. Pl. 1: 592. 1898.
Vernonia angolensis (O. Hoffm.) N.E. Brown, Kew Bull. 1909: 116. 1909.
Vernonia lutea N.E. Brown, Kew Bull. 1909: 116. 1909.
Distephanus angolensis (O. Hoffm.) H. Rob. & B. Kahn, Proc. Biol. Soc. Wash. 99(3): 498. 1986. SW Africa.

##### Distribution.

Angola, Namibia.

#### Distephanus angulifolius

Taxon classificationPlantaeAsteralesAsteraceae

(DC.) H. Rob. & B. Kahn, 1986

Vernonia angulifolia DC., Prodr. 5: 29. 1836.
Distephanus angulifolius (DC.) H. Rob. & B. Kahn, Proc. Biol. Soc. Wash. 99(3): 499. 1986.

##### Note.

Jeffrey (1988) mentioned *Vernonia biafrae* Oliv. & Hiern in Oliv. was once placed in the synonymy of this species by Maquet in Troupin (1985), but cited a number of differences that did not include the strictly pinnate venation of the more northern *Vernonia biafrae* [= *Distephanus biafrae* (Oliv. & Hiern in Oliv.) H. Rob.].

##### Distribution.

Mozambique, South Africa (Natal, Transkei).

#### Distephanus anisochaetoides

Taxon classificationPlantaeAsteralesAsteraceae

(Sond.) H. Rob. & B. Kahn, 1986

Vernonia anisochaeoides Sond., Linn., 23: 61. 1850.
Distephanus anisochaetoides (Sond.) H. Rob. & B. Kahn, Proc. Biol. Soc. Wash. 99(3): 499. 1986.

##### Distribution.

South Africa (Cape colony, Natal).

#### Distephanus divaricatus

Taxon classificationPlantaeAsteralesAsteraceae

(Steetz) H. Rob. & B. Kahn, 1986

Gongrothamnus divaricatus Steetz in Peters, Reise Mossamb. Bot. 242. 1864.
Gongrothamnus aurantiacus O. Hoffm., Bot. Jahrb. Syst. 30: 433. 1902.
Vernonia aurantiaca (O. Hoffm.) N. E. Brown, Kew Bull. 1909: 116. 1909.
Vernonia vitellina N. E. Brown, Kew Bull. 1909: 117. 1909.
Gongrothamnus corradianus Cufod., Nouvo Giorn. Bot. Ital. n.s. 1: 111. 1943.
Distephanus divaricatus (Steetz) H. Rob. & B. Kahn, Proc. Biol. Soc. Wash. 99(3): 499. 1986.

##### Distribution.

Angola, Botswana, Congo, Ethiopia, Kenya, Malawi, Mozambique, Namibia, Tanzania, South Africa (Transvaal), Zambia, and Zimbabwe.

#### Distephanus inhacensis

Taxon classificationPlantaeAsteralesAsteraceae

(Pope) Boon & Glen, 2013

Vernonia inhacensis G.V. Pope, Kew Bull. 43(2): 280. 1988.
Distephanus inhacensis (Pope) Boon & Glen, Bothalia 43: 94. 2013.

##### Distribution.

Mozambique and South Africa (Natal).

#### Erlangea

Taxon classificationPlantaeAsteralesAsteraceae

Sch. Bip., 1853

**Figure 7.**
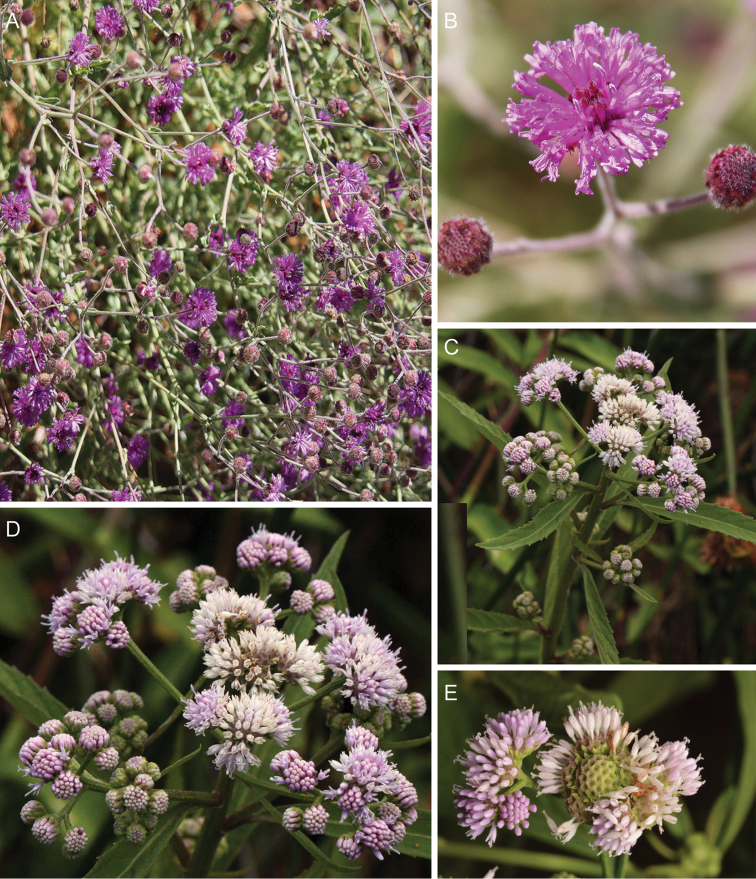
Photographs of *Erlangea* and *Ethulia*: **A–B** *Erlangea misera* S. Moore, and **C–E** Ethulia conyzoides L.f. subsp. conyzoides, note: *Ethulia* has no capillary pappus. See Appendix C for citation details.

**Figure 8.**
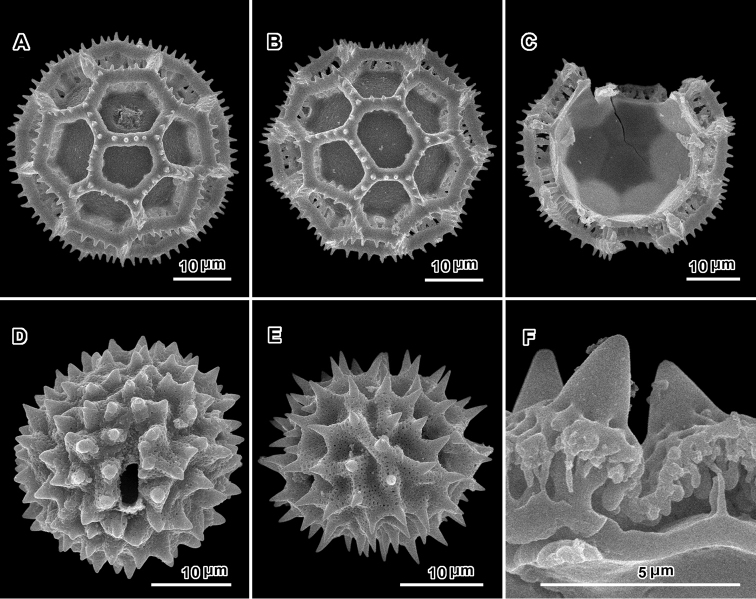
Scanning electron micrographs of acetolyzed echinolophate pollen of *Erlangea* and sublophate pollen of *Ethulia*. **A–C** *Erlangea misera* (Oliv. & Hiern) S. Moore. **A** Poral view **B** Near polar view **C** Grain fragment. **D–F** *Ethulia conzyoides* L.f. **D** Equatorial view, showing comparatively blunt spines **E** Lateral view **F** Grain fragment. (**D–F** Funk 12708 **G** *Petelot 4047* **H** *Lewis 6025*; *Ethulia* views from Robinson and Skvarla (2010)).

Figures 7 A, B ; 8 A–C

Erlangea Sch. Bip., 1853, Flora 36: 34. 1853. – Type: *Erlangea plumosa* Sch. Bip.

##### Resources.

Species treatment based on Wild and Pope 1977.

##### Descriptions.

Annual or short-lived perennial herbs; stems erect, branching near base; hairs on vegetative parts simple, uniseriate, multicellular, with a straight elongate apical cell. Leaves alternate, sessile or subsessile, pinnately veined with weak secondary veins, margins serrulate, apices obtuse. Inflorescence with single terminal head or laxly cymiform with narrowly pedunculate heads. Heads campanulate; involucral bracts 45–60 in 3–4 series, gradate, cuspidate apically, with distinct pale or reddish lateral margins, pilose to lanulose outside; receptacle convex, epaleaceous, with glabrous reticulum. Florets 50–75 or more in a head; corollas reddish, funnelform, with slender basal tube bearing small stipitate glands, throat shorter than lobes, lobes linear-lanceolate, with stiff hairs distally or apically; anther thecae short-acute with small sterile margin at base; apical appendage, oblong-ovate, glabrous, with thin cell walls; style base with narrow annuliform sclerified node; sweeping hairs acicular, at lowest level scarcely extending to top of shaft. Achenes shortly obconic, abruptly narrowed distally to insertion of corolla, 3-6-ribbed, setulae restricted mostly to broad ribs, setulae not split at tips, sides with scattered isolated idioblasts, raphids subquadrate or short oblong in dense inner layer of short to quadrate cells in achene wall; pappus of less than 20 easily deciduous barbellate bristles, bases narrow and weakly attached, distinct outer series not evident. Chromosome number n = 10 (Turner and Lewis 1965, Nordenstam 1967).

Pollen ca. 47 μm in diameter in fluid, lophate, triporate, with group of polar lacunae, perforated tectum restricted to muri, bacculae centered at junctures of muri, leaving ogee-shaped gaps under the centers of the muri (Figs 8 A–C).

Notable secondary metabolites, eudesmanolide sesquiterpene lactones, Bohlmann and Jakupovic 1990, as *Erlangea remifolia* Wild & Pope).

#### Key to the species of *Erlangea*

**Table:** 

1	Leaves sessile or subsessile; blades linear to oblong ot ovate-oblong	*Erlangea misera*
–	Leaves distinctly petiolate, with petioles to 1.5 cm long; blades ovate	*Erlangea remifolia*

#### Erlangea misera

Taxon classificationPlantaeAsteralesAsteraceae

(Oliv. & Hiern) S. Moore, 1902

Vernonia misera Oliv. & Hiern in Oliv., Fl. Trop. Afr. 3: 278. 1877.
Erlangea schinzii O. Hoffm., Bull. Herb. Boiss. 1: 71. 1893
Bothriocline misera (Oliv. & Hiern) O. Hoffm., Bot. Soc. Brot. 13: 11. 1896.
Erlangea misera (Oliv. & Hiern) S. Moore, J. Linn. Soc., Bot. 35: 310. 1902.
Bothriocline schinzii (O. Hoffm.) O. Hoffm. in Warburg, Kunene-Sambesi Exped. 398. 1903.
Erlangea sessilifolia R.E. Fr., Wiss. Ergebn. Schwed. Rhodesia-Kongo-Exped. 1911–1912, 1: 319. 1916.
Vernonia merenskiana Dinter ex Merxm., Mitt. Bot. Münchem 2: 38. 1954, nom. nud. in syn.

##### Distribution.

Botswana, Mozambique, Namibia (Caprivi strip), Zambia, Zimbabwe.

#### Erlangea remifolia

Taxon classificationPlantaeAsteralesAsteraceae

Wild & G.V. Pope, 1977

Erlangea remifolia Wild & G.V. Pope, Kirkia 10(2): 317. 1977.

##### Distribution.

Botswana.

#### Ethulia

Taxon classificationPlantaeAsteralesAsteraceae

L.f.

Figures 7 C–E ; 8 D–F

Ethulia L.f. Dec. Prima Pl. Rar. Horti Upsal. 1 (1762); L.f. ex L., Sp. Pl. ed. II: 1171 (1763). – Type: *Ethulia conyzoides* L.f.
Hoehnelia Schweinf. in Höhnel, Zum Rudolf-See und Stephanie-See 86 (1892). – Type: *Hoehnelia vernonioides* Schweinf. in Höhnel.

##### Resources.

Treatment of the genus by Gilbert and Jeffrey (1988).

##### Descriptions.

Annual or short-lived perennial herbs, rarely rhizomatous; stems terete and usually striate, with broad solid pith; hairs uniseriate with erect apical cells, with glandular dots. Leaves alternate, sessile or short petiolate; blades thinly herbaceous, ovate to linear lanceolate, base cuneate or continuous onto stem, margins subentire to serrate or dentate, apex acute to obtuse, surfaces glabrous to densely pubescent; venation pinnate with ascending secondary veins. Inflorescence terminal, corymbiform to rather cymiform, lower bracteoles a reduced foliiform, peduncular bracteoles filiform. Heads rather small, with broadly campanulate involucres; involucral bracts 15–40 in 2–3 usually subequal series; receptacle flat or slightly convex, epaleaceous. Florets 3–100 in a head, strongly exserted; corollas white or pink to purple, with glandular dots on surface, with a narrow cylindrical base, limb narrowly funnelform to narrowly campanulate; lobes lanceolate, without apical hairs; bases of anther thecae rounded, not tailed; apical appendages glabrous; style base without node; branches with sweeping hairs shortly acute. Achenes cylindrical with 2–6 usually paler ribs, sides with glandular dots, rarely with short white setulae; raphids short-oblong; pappus lacking or a coroniform rim. Chromosome number n = 10, 20 (Pilz 1980; Gilbert and Jeffrey 1988).

Pollen: ca. 35 μm in diam. in fluid; tricolporate, sublophate, echinate, spines long; tectum continuous in intercolpi and at poles, distinctly microperforate; columellae below spines firmly attached to footlayer (Fig. 8 D–F).

Notable secondary metabolites: 5-alkylcoumarins (Bohlmann and Jakupovic 1990, as *Ethulia conyzoides*).

#### Ethulia conyzoides

Taxon classificationPlantaeAsteralesAsteraceae

L.f., 1762

Ethulia conyzoides L.f., Decas Prima Pl. Rar. Horti Upsal. 1, pl. 1. 1762.
Ethulia ramosa Roxb., Hort. Beng. 61. 1814.
Ethulia gracilis Delile in Cailliaud., Voy. Meroe 4: 398. 1827.

##### Distribution.

Tropical and southern Africa, Asia to China, introduced in Brazil.

#### Gymnanthemum

Taxon classificationPlantaeAsteralesAsteraceae

Cass., 1817

**Figure 9.**
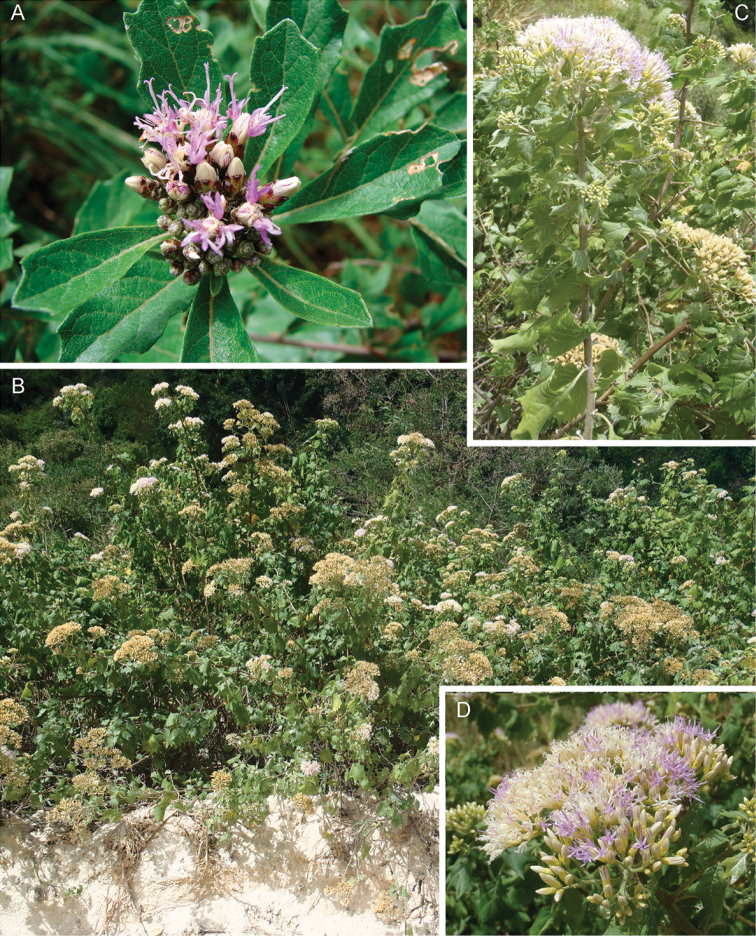
Photographs of *Gymnanthemum*: **A** *Gymnanthemum corymbosum* (Thunb.) H. Rob. **B–D** *Gymnanthemum capense* (A. Spreng.) J. C. Manning & N. Swelankomo. See Appendix C for citation details.

**Figure 10.**
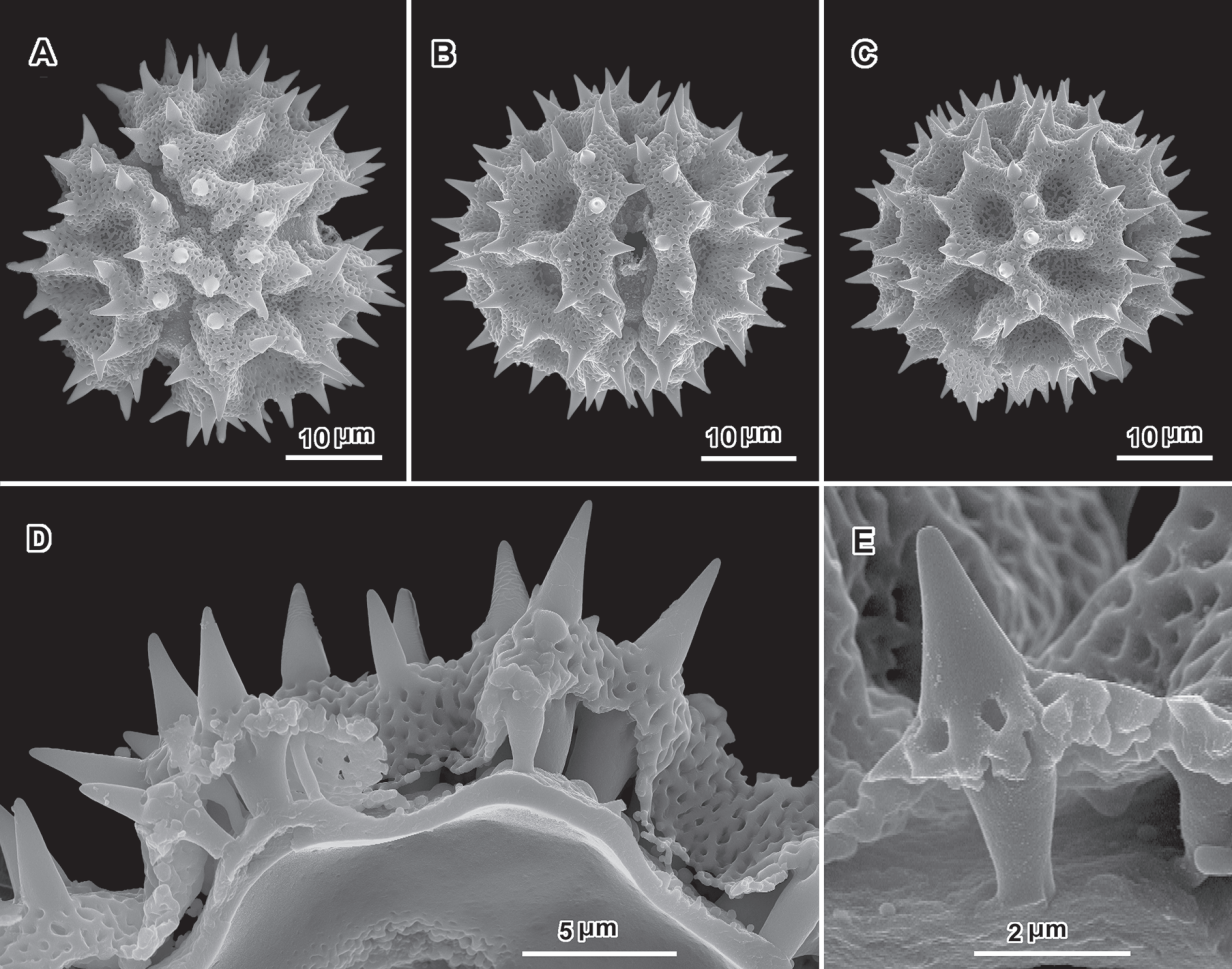
Scanning electron micrographs of acetolyzed pollen of two collections of sublophate echinolophate *Gymnanthemum capense* (A. Spreng.) J. C. Manning & N. Swelankomo. **A** Polar view **B** Equatorial view **C** Lateral view **D** Fractured grain structure of exine surfaces **E** Fractured grain showing spine construction. (**A–C** *Schlechter 6644*).

Figures 9 A–D ; 10 A–E

Gymnanthemum Cass. Bull. Soc. Philom. Paris 1817: 10. 1817. – Type: *Gymnanthemum cupulare* Cass. = *Baccharis senegalensis* = *Gymnanthemum coloratum* (Willd.) H. Rob. & B. Kahn
Bracheilema R. Br. ex Salt., Abyss. Append. 65. 1814, nom. nud.
Decaneurum DC., Arch. Bot. (Paris) 2: 516. 1833, nom. superfl., type same as *Gymnanthemum*.
Plectreca Rafin., Fl. Tellur. 4: 119. 1838. – Type: *Staehelina corymbosa* Thunb.
Keringa Rafin., Sylva Tellur. 144. 1838. – Type: *Vernonia amygdalina* Del.
Cheliusia Sch. Bip. in Hochst., Flora 24 [Intell. 1(2)] 26. 1841, nom. nud. – *Cheliusia abyssinica* Sch. Bip. = *Gymnanthemum amygdalinum* (Del.) Sch. Bip. ex Walp.
Vernonia subsect. Urceolata S.B. Jones, Rhodora 83: 67. 1981. – Type: *Vernonia sphaerocalyx* O. Hoffm.

##### Descriptions.

Shrubs or small trees, moderately to densely branching; stems mostly terete, with solid pith; hairs of stem often forming a felt, with large often contorted cap cells basally or nearly basally attached. Leaves alternate; petioles short, winged or elongate; blades membranaceous to rather coriaceous, margins entire to serrate or repand dentate, upper surfaces essentially glabrous and somewhat glossy to arachnoid tomentose; secondary veins pinnate, spreading at 30–80° angles, arching nearer margins. Inflorescences terminal, densely corymbiform, with small bracteoles; peduncles short. Heads with campanulate to cylindrical or ovoid involucres; involucral bracts coriaceous to subcoriaceous, appressed, 25–35 in 4–5 gradate series, inner bracts persistent to easily deciduous, outer surface with smooth median shield, without narrow median costa or keel; receptacles epaleaceous. Florets 5–50 in a head; corollas white to violet, basal tube cylindrical, throat longer than the anther thecae or very deeply cut, lobes with glands or spicules on outer surface; anther thecae with base broadly tailed, tails often long; apical appendages glabrous, with rather thick-walled cells; style base without or with scarcely distinct node; style branches with stout, pointed sweeping hairs. Achenes 5–10-costate, with or without setulae, raphids short to elongate, sometimes not evident; pappus of many rather persistent capillary bristles, often with broadened tips, with outer series of short squamellae. Chromosome numbers n = 10, 15, 20 (Jones 1970, 1982; Adegbite and Ayodele 2004).

Pollen: 30–35 μm in diam. (dry); tricolporate, echinate, sublophate; tectum continuous in intercolpi and at poles, with distinct microperforations; spines long, each with single stout columella below firmly attached to footlayer, intervening perforated tectum scarcely mamillose on inner surface (Fig. 10 A–E).

Generic limits more restricted than given in Robinson (1999a), see Robinson and Skvarla (2006, 2007) and Robinson et al. (2008).

A special effort has been made to resolve the endemic southern African element of *Gymnanthemum* that includes *Gymnanthemum corymbosum* and *Gymnanthemum capense* (Swelankomo et al. 2015).

#### Key to the species of *Gymnanthemum*

**Table:** 

1	Capitula with 9–30 florets	**2**
–	Capitula with 2–5 florets	**4**
2	Leaves sessile, usually auriculate at base	***Gymnanthemum theophrastifolium***
–	Leaves with distinct petioles	**3**
3	Achenes with setulae on the surface	***Gymnanthemum coloratum***
–	Achenes without setulae	***Gymnanthemum amygdalinum***
4	Leaf blades elliptical, with sharply serrate margins	***Gymnanthemum myrianthum***
–	Leaf blades suborbicular to narrowly obovate, with repand-dentate distal margins	**5**
5	Leaves sparsely puberulous to essentially glabrous abaxially	**6**
–	Leaves hispid to tomentose abaxially	**7**
6	Leaf blades chartaceous, with broadly obtuse bases; stems puberulous with often dark hairs	***Gymnanthemum koekemoerae***
–	Leaf blades rather membranaceous with long-acuminate bases; stems essentially glabrous	***Gymnanthemum capense***
7	Leaf blades oblong to ovate with obtuse bases; stems hirsute; capitula with 3 florets	***Gymnanthemum triflorum***
–	Leaf blades obovate to oblanceolate with cuneate bases; stems tomentose; capitula usually with 4–5 florets	**8**
8	Stems and abaxial surfaces of leaves completely covered with appressed tomentum; inflorescence narrowly corymbose	***Gymnanthemum corymbosum***
–	Stems with tomentum of cottony hairs, abaxial surfaces of leaves with mixed erect and arachnoid hairs that do not totally obscure green surface; inflorescence broadly corymbose, much broader than high	***Gymnanthemum crataegifolium***

#### Gymnanthemum amygdalinum

Taxon classificationPlantaeAsteralesAsteraceae

(Del.) Sch. Bip. ex Walp., 1843

Vernonia amygdalina Del., Cent. Pl. Afr. Voy. Méroé 41. 1826.
Gymnanthemum amygdalinum (Del.) Sch. Bip. ex Walp., Rep. 2: 948. 1843.
Gymnanthemum abyssinicum Sch. Bip. ex Walp., Rep. 2: 948. 1843.
Vernonia vogeliana Benth. in Hook., Niger Fl. 427. 1849.
Vernonia condensata Baker, J. Bot. 8: 202. 1875.
Vernonia eritreana Klatt, Bull. Herb. Boiss. 4: 826. 1896.
Vernonia randii S. Moore, J. Bot. 37: 369. 1899.
Vernonia giorgii De Wild., Bull. Jard. Bot. Brux. 5: 92. 1915.
Vernonia bahiensis Toledo, Arq. Bot. Estado Sao Paulo, n.s. 1: 52. 1939.
Vernonanthura condensata (Baker) H. Rob., Phytologia 73: 69. 1992.

##### Note.

The species is used as medicinal plant by both people and animals.

##### Distribution.

Africa and introduced into Brazil.

#### Gymnanthemum capense

Taxon classificationPlantaeAsteralesAsteraceae

(A. Spreng.) J. C. Manning & N. Swelankomo, 2015

Eupatorium capense A. Spreng., Tent. Suppl. 22. 1828.
Vernonia mespilifolia Less., Linnaea 6: 641. 1831, nom. superfl.
Gymnanthemum mespilifolium (Less.) H. Rob., Proc. Biol. Soc. Wash. 112(1): 242. 1999.
Gymnanthemum capense (A. Spreng.) J. C. Manning & N. Swelankomo, S. African J. Bot. 101: 12. 2015.

##### Distribution.

Transvaal, Natal, Swaziland, Cape colony.

##### Note.

Swelankomo et al. (2015) point out that the older name *Eupatorium capense* should have been used for this species, and they make the necessary new combination. The combination *Vernonia capensis* has been used since 1917 for another species (now in *Hilliardiella*). At this time there is still no unpreoccupied name for the species that has been called *Vernonia mespilifolia* in the genus *Vernonia*. Two specimens examined: Rogers 28651 from Grahamstown, and C.E. Smith & Duthie 4678 from Natal, the latter originally distributed as *Vernonia crataegifolia*.

#### Gymnanthemum coloratum

Taxon classificationPlantaeAsteralesAsteraceae

(Willd.) H. Rob. & B.Kahn, 1986

Eupatorium coloratum Willd., Sp. Pl. 3: 1769. 1803.
Baccharis senegalensis Pers., Syn. Pl. 2: 424. 1807.
Gymnanthemum cupulare Cass., Dict. Sc. Nat. ed. 2, 20: 109. 1821.
Vernonia senegalensis (Pers.) Less., Linnaea 4: 265. 1829.
Decaneurum grande DC., Prodr. 5: 67. 1836.
Decaneurum senegalense (Pers.) DC., Prodr. 5: 68. 1836.
Gymnanthemum grande (DC.) Sch. Bip. ex Walp., Rep. 2: 948. 1843.
Gymnanthemum senegalense (Pers.) Sch. Bip. ex Walp., Rep. 2: 948. 1843.
Gymnanthemum quercifolium Steetz in Peters, Reise Mossamb. Bot. 334. 1864.
Vernonia oxyura O. Hoffm. in Engler, Pflanzenw. Ost.-Afr. C. 403. 1895.
Vernonia polyura O. Hoffm., Bot. Jahrb. Syst. 30: 422. 1901.
Vernonia cirrifera S. Moore, J. Linn. Soc. Bot. 35: 320. 1902.
Vernonia longipetiolata Muschl., Bot. Jahrb. Syst. 46: 74. 1911.
Vernonia aldabrensis Hemsl., J. Bot. 54: suppl., 2: 20. 1916.
Vernonia grandis (DC.) Humb., Fl. Madag. 189: 44. 1960.
Gymnanthemum coloratum (Willd.) H. Rob. & B.Kahn, Proc. Biol. Soc. Wash. 99: 501. 1986.

##### Distribution.

Tropical and subtropical Africa.

#### Gymnanthemum corymbosum

Taxon classificationPlantaeAsteralesAsteraceae

(L.f.) H. Rob., 1999

Staehelina corymbosa L. f., Suppl. 359. 1781.
Vernonia corymbosa (L. f.) Less., Linnaea 6: 647. 1831, nom. illeg., non *Vernonia corymbosa* Schwein. ex Keating, Narr. Exp. Long. 2: 394. 1824.
Plectreca corymbosa (L. f.) Raf., Fl. Tellur. 4: 119. 1838 (“1836”).
Vernonia neocorymbosa Hilliard, Notes Roy. Bot. Gard. Edinburgh 32(3): 385. 1973. New name for *Vernonia corymbosa*.
Gymnanthemum corymbosum (L.f.) H. Rob., Proc. Biol. Soc. Wash. 112(1): 241. 1999.

##### Distribution.

Eastern South Africa through Swaziland and Natal, Transkei, s. Mosambique.

##### Note.

Many specimens from South Africa been seen including Schlechter 6644 distributed under the name *Vernonia angulifera* DC., nom. nud.

#### Gymnanthemum crataegifolium

Taxon classificationPlantaeAsteralesAsteraceae

(Hutch.) H. Rob., 1999

Vernonia mespilifolia Less. var. subcanescens DC., Prodr. 5: 29. 1836.
Vernonia crataegifolia Hutch., Bull. Misc. Inf. Kew 7: 330. 1912.
Vernonia pseudocorymbosa Thell., Vierteljahrsschr. Nat. Ges. Zurich, 68: 440. 1923.
Gymnanthemum crataegifolium (Hutch.) H. Rob., Proc. Biol. Soc. Wash. 112(1): 241. 1999.

##### Distribution.

South Africa (Transvaal, Natal, Swaziland, Cape colony).

##### Note.

The species is known in this study from descriptions, from photographs of the syntype, Clydesdale, *Tyson 1188* (K) deposited at the US by Earl Smith, and one specimen, Sidey 3470 from Natal, distributed originally as *Vernonia corymbosa*.

#### Gymnanthemum koekemoerae

Taxon classificationPlantaeAsteralesAsteraceae

H. Rob. & V.A. Funk, 2014

Gymnanthemum koekemoerae H. Rob. & V.A. Funk, Phytokeys 36: 60. 2014.

##### Type matherial.

Holotype: South Africa. Limpopo Province: Thohoyandou District; Thathe-Vonde Nature Reserve. Grassland at rocky outcrop near entrance, 1233 m, 22°55'10"S, 30°19’36”E [2230CD], 23 March 2002, *Koekemoer 2273* (PRE, isotype US) (Fig. 2D).

The type specimen was distributed as *Vernonia triflora* Brem., which differs by having only 3 florets in its capitula, stiffly and densely hispid stems, and ovate to oblong leaf blades with hispidulous abaxial surfaces.

##### Distribution.

South Africa

#### Gymnanthemum myrianthum

Taxon classificationPlantaeAsteralesAsteraceae

(Hook.f.) H. Rob., 1999

Vernonia myriantha Hook.f., J. Linn. Soc. Bot.7: 198. 1864.
Vernonia podocoma Sch. Bip. ex Vatke, Linnaea 39: 476. 1875.
Vernonia subuligera O. Hoffm. in Engler, Pflanzenw. Ost-Afr. C. 403. 1895.
Vernonia stipulacea Klatt, Bull. Herb. Boiss. 4: 457. 1896.
Vernonia lujae De Wild., Pl. Nov. Herb. Hort. Then. 2: 119, t. 96. 1900.
Vernonia ampla O. Hoffm., Bot. Jahrb. Syst. 30: 423. 1901.
Vernonia myrianthoides Muschl., Bot. Jahrb. Syst. 46: 84. 1911.
Vernonia uhligii Muschl., Bot. Jahrb. Syst. 46: 84. 1911.
Vernonia oliveriana Pichi-Serm., Webbia 7: 345. 1950, nom. illeg. superfl. for *Vernonia podocoma* Sch. Bip. ex Vatke
Vernonia chlarugii Pichi-Serm., Miss. Stud. Lago Tana 7, Ricerche Bot. 1: 155, t. 30. 1951.
Gymnanthemum myrianthum (Hook.f.) H. Rob., Proc. Biol. Soc. Wash. 112(1): 242. 1999.

##### Distribution.

West Africa from Guinea and Sierra Leone to Cameroon, Sudan, Ethiopia, Kenya, Uganda, Congo, south to South Africa (Transvaal, Natal), and Swaziland.

#### Gymnanthemum theophrastifolium

Taxon classificationPlantaeAsteralesAsteraceae

(Schweinf. ex Oliv. & Hiern) H. Rob., 1999

Vernonia theophrastifolia Schweinf. ex Oliv. & Hiern, Fl. Trop. Afr. 3: 294. 1877.
Vernonia myriocephala A. Rich., Tent. Fl. Abyss. 1: 374. 1848, nom. illeg., not DC. 1836.
Cacalia richardiana O. Kuntze, Rev. Gen. Pl. 2: 969. 1891, nom. nov. for *Vernonia myriocephala* A. Rich.
Vernonia seretii De Wild., Ann. Mus. Congo Belge, Bot. ser., 5(2): 207. 1907.
Vernonia macrophylla Chiov., Ann. Bot. Roma 9: 70. 1911.
Vernonia richardiana (O. Kuntze) Pichi-Serm., Webbia 7: 340. 1950.
Gymnanthemum theophrastifolium (Schweinf. ex Oliv. & Hiern) H. Rob., Proc. Biol. Soc. Wash. 112(1): 243. 1999.

##### Distribution.

Congo and Nigeria east to Uganda, Kenya, Ethiopia, and south to South Africa.

#### Gymnanthemum triflorum

Taxon classificationPlantaeAsteralesAsteraceae

(Bremek.) H. Rob., 2005

Vernonia triflora Bremek., Ann. Transvaal Mus. 15: 262. 1933.
Gymnanthemum triflorum (Bremek.) H. Rob., Phytologia 87(2): 80. 2005.

##### Distribution.

South Africa (Transvaal).

##### Note.

One specimen has been seen, Stalmans 2430AA, from South Africa (Transvaal), that matched the original description in every respect except for the lack of noticeable pubescence on the involucral bracts.

#### Hilliardiella

Taxon classificationPlantaeAsteralesAsteraceae

H. Rob., 1999

**Figure 11.**
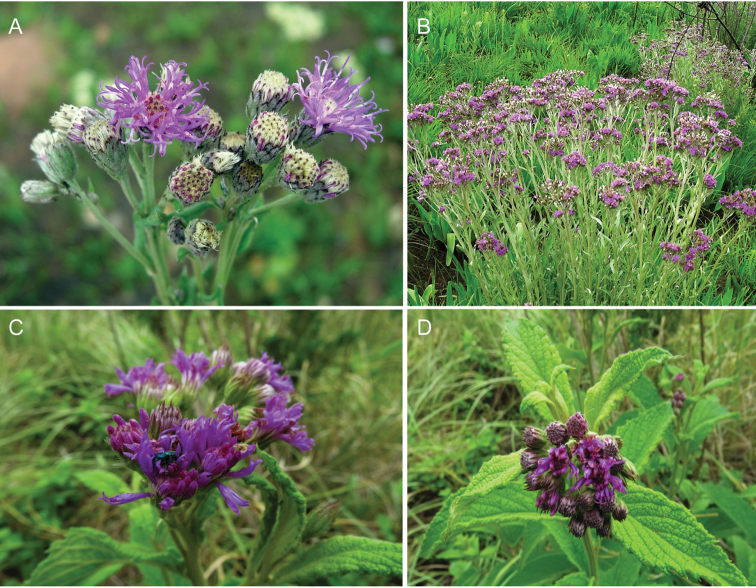
Photographs of *Hilliardiella*. **A–B** *Hilliardiella aristata* (DC.) H. Rob. **C–D** *Hilliardiella flanaganii* (E. Phillips) H. Rob. See Appendix C for citation details.

**Figure 12.**
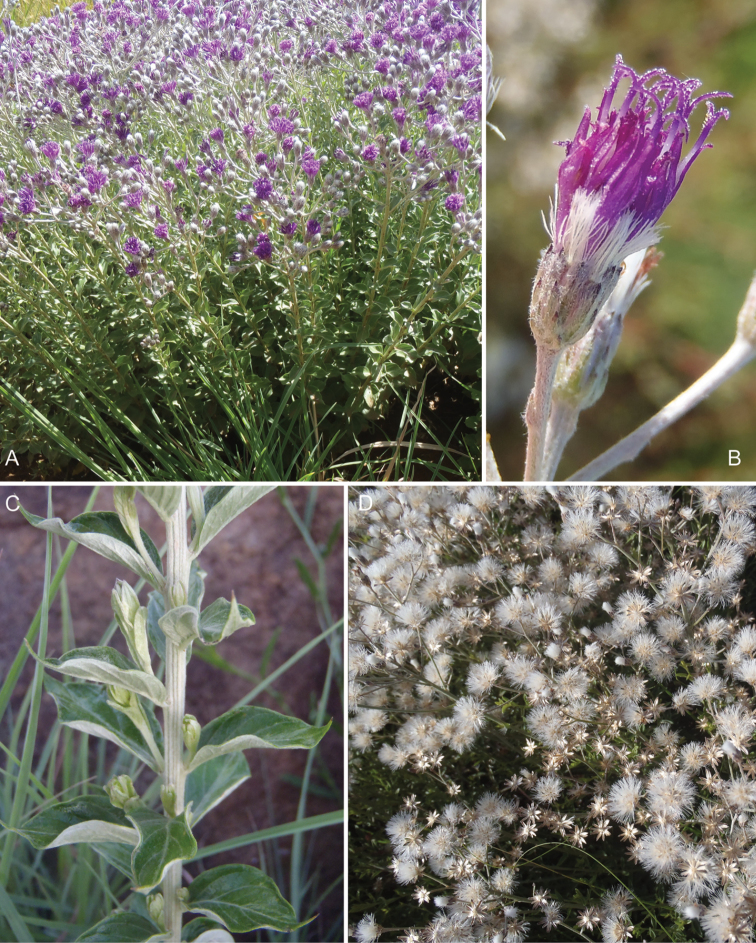
Photographs of *Hilliardiella oligocephala* (DC.) H. Rob.: **A** Habit **B** Close up of head **C** Characteristic leaf **D** heads in fruit. Note: discolorous leaves are characteristic of some but not all species of *Hilliardiella*. See Appendix C for citation details.

**Figure 13.**
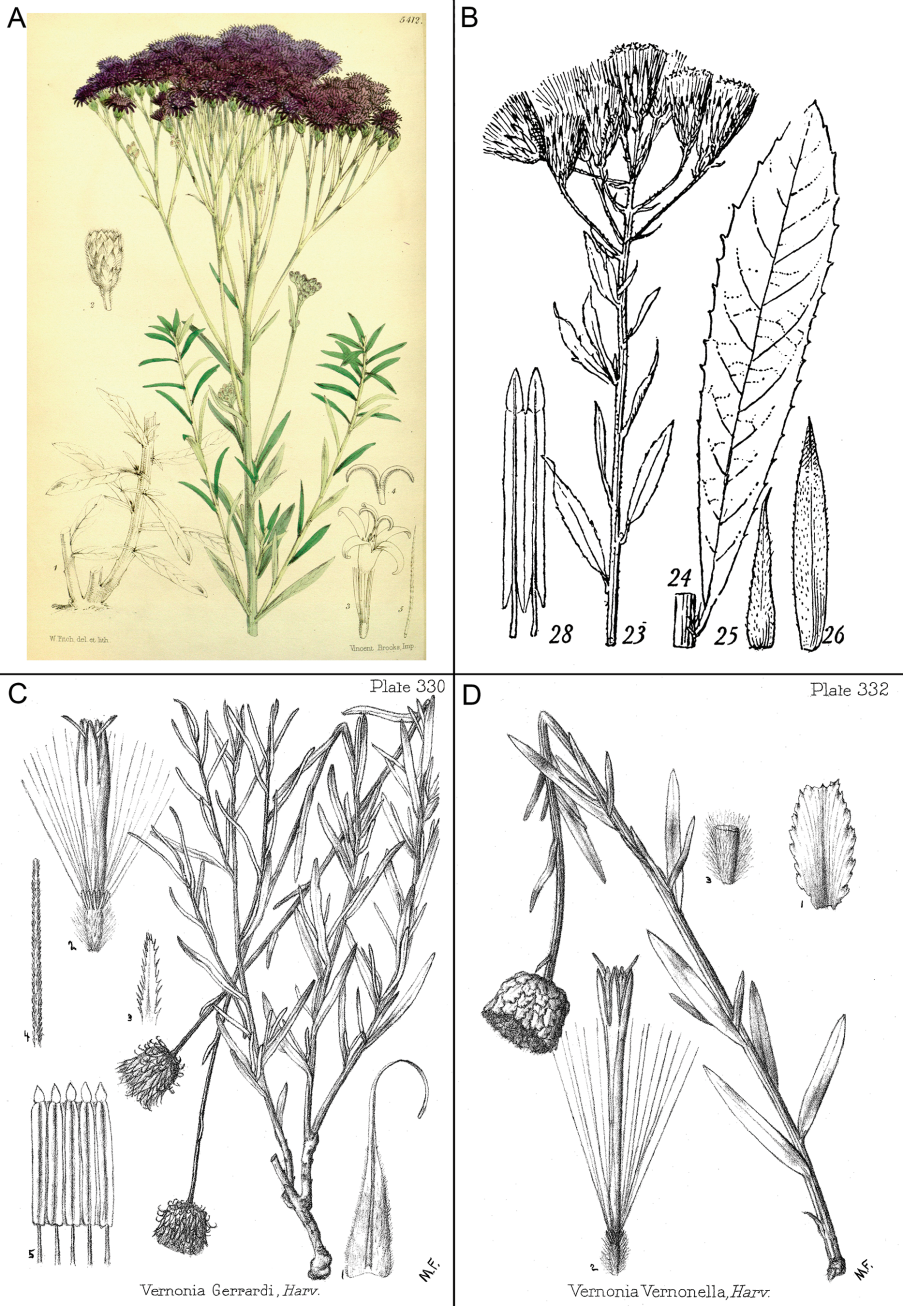
Illustrations *Hilliardiella*, *Linzia*, *Parapolydora*, and *Vernonella*: **A** *Hilliardiella capensis* (Houtt.) H. Rob., Skvarla & V.A. Funk **B** *Linzia glabra* Steetz in Peters, note: characteristic teeth on involucral bracts **C** *Parapolydora gerrardii* (Harv.) H. Rob **D** *Vernonella africana* Sond. See Appendix C for citation details.

**Figure 14.**
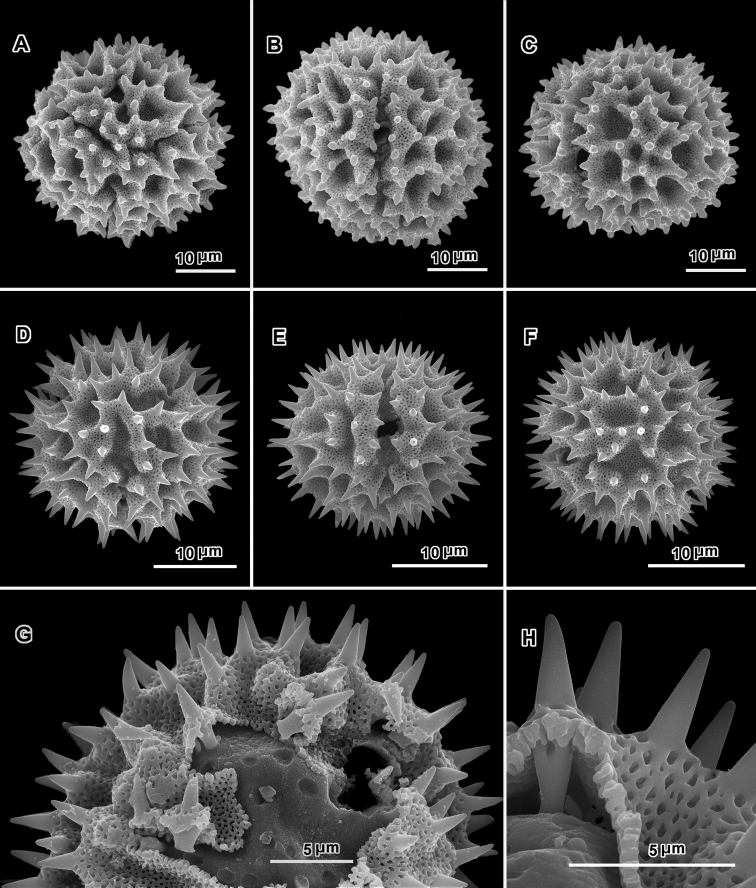
Scanning electron micrographs of acetolyzed pollen from two collections of *Hilliardiella capensis* emphasizing spine variations. The surface seems to vary between sublophate and slightly echinolophate. **A** Polar view **B** Equatorial view **C** Lateral view **D** Polar view **E** Equatorial view **F** Subpolar view **G** Fragmented pollen surface **H** Grain fragment showing structural support of spine. (**A–C** *Bayliss BS3686* **D–H** *Gentry & Barolas 18914*).

Figures 11 A–D ; 12 A–D ; 13 A ; 14 A–H

Hilliardiella H. Rob. Proc. Biol. Soc. Wash. 112(1): 247. 1999. – Type: *Vernonia pinifolia* (Lam.) Less.
Webbia DC., Prodr. 5: 72. Oct 1836, nom. illeg., not *Webbia* Spach, Jun 1836.
Vernonia subsect. Hilliardianae S.B. Jones, Rhodora 83: 66. 1981. – Type: *Vernonia oligocephala* (DC.) Sch. Bip.

##### Descriptions.

Herbaceous perennials to 1 m tall; stems pilose, hairs unequally T-shaped. Leaves alternate; blades abaxially often densely canescent pilose. Inflorescence laxly to subdensely corymbiform-cymose. Heads short-pedunculate; involucres campanulate, bracts 25–40, in ca. 3–4 series, persistent; receptacle epaleaceous. Florets 12–20 in a head; corollas purple, outside with few to many slightly contorted T-shaped hairs; basal tube funneliform above, throat short, lobes linear; anther thecae not or shortly appendaged at base; apical appendages glabrous, with thin walls; style with basal node; style branches with acicular sweeping hairs. Achenes 4–5-costate, densely setuliferous, setulae scarcely divided at tips, idioblasts numerous, raphids elongate, carpopodia narrowly cylindrical; pappus bristles white, barbate, tenuous, subpersistent, outer series shortly lanceolate. Chromosome number of n = 9, 10, most reports n = 10 (Turner and Lewis 1965; Jones 1982).

Pollen grains sublophate, with continuous perforated tectum between colpi, tricolporate to poles, echinate (Fig. 14 A–H).

Notable secondary metabolites; acetones & sesquiterpene glaucolides/ hirsutanolides (Bohlmann and Jakupovic (1990, as *Vernonia sutherlandii* Harv., guaianolides, bisabolene derivatives (Bohlmann and Jakupovic 1990, as *Vernonia hirsuta* Sch. Bip. ex Walp. and *Vernonia oligocephala* (DC.) Sch. Bip. ex Walp.).

#### Key to the species of *Hilliardiella*

**Table:** 

1	Leaves mostly basal, not cauline	***Hilliardiella nudicaulis***
–	Leaves disposed rather uniformly along stems	**2**
2	Leaf surfaces coarsely pubescent, not sericeous	**3**
–	One or both surfaces of leaves sericeous with silvery pubescence	**4**
3	Leaves ovate to ovate-elliptic, 1.5–3 times as long as wide	***Hilliardiella oligocephala***
–	Leaves linear, ca. 2 m wide, 12 or more times as long as wide	***Hilliardiella capensis***
4	Both leaf surfaces densely silvery sericeous; longest phyllaries 5–8.5 mm long	**5**
–	Upper leaf surface dark; longest phyllaries 2.3–5 mm long	**6**
5	Larger involucral bracts with caudate apices; capitula less than 1.5 cm wide	***Hilliardiella aristata***
–	Involucral bracts acuminate, not caudate; capitula ca. 1.5 cm wide	***Hilliardiella pseudonatalensis***
6	Leaves acute at base; pubescence on leaf surfaces not obscuring the surfaces, numerous large glandular dots visible on abaxial surface	***Hilliardiella sutherlandii***
–	At least upper leaves cordate at base, pubescence on abaxial leaf surface mostly obscuring presence of glandular dots	**7**
7	Bases of lower leaves narrow; tips of phyllaries long-acuminate, equaling or exceeding the pappus	***Hilliardiella flanaganii***
–	Bases of lower leaves cordate; phyllaries without long-acuminate tips equaling or exceeding the pappus	***Hilliardiella hirsuta***

#### Hilliardiella aristata

Taxon classificationPlantaeAsteralesAsteraceae

(DC.) H. Rob., 1999

Webbia aristata DC., Prodr. 5: 73. 1836.
Vernonia natalensis Sch. Bip. ex Walp., Rep. 2: 947. 1843.
Hilliardiella aristata (DC.) H. Rob., Proc. Biol. Soc. Wash. 112(1): 230. 1999.

##### Distribution.

Lesotho, South Africa (Transvaal, Orange Free State, Natal, Cape colony), and Swaziland.

#### Hilliardiella capensis

Taxon classificationPlantaeAsteralesAsteraceae

(Houtt.) H. Rob., Skvarla & V.A. Funk comb. nov.

urn:lsid:ipni.org:names:77152896-1

Erigeron capensis Houtt., Handl. Pl.-Kruidk. 10: 629. 1773–1783.
Conyza pinifolia Lam., Encycl. (Lamarck) 2(1): 86. 1786 [16 Oct 1786]
Conyza canescens Thunb., Fl. Cap. 665. 1823.
Vernonia pinifolia (Lam.) Less., Linnaea 4: 257. 1829.
Webbia pinifolia (Lam.) DC., Prodr. 5: 72. 1836.
Vernonia capensis (Houtt.) Druce, Rep. Bot. Exch. Cl. Brit. Isles 1916: 651. 1917.
Hilliardiella pinifolia (Lam.) H. Rob., Proc. Biol. Soc. Wash. 112(1): 230. 1999.

##### Distribution.

Lesotho, South Africa (Transvaal, Orange Free State, Natal, Cape colony) and Swaziland.

##### Note.

This complete synonymy shows that the oldest name for the species is *Erigeron capensis* Houtt.

#### Hilliardiella flanaganii

Taxon classificationPlantaeAsteralesAsteraceae

(E. Phillips) H. Rob., Skvarla & V.A. Funk comb. nov.

urn:lsid:ipni.org:names:77152902-1

Vernonia hirsuta (DC.) Sch. Bip. ex Walp. var. flanaganii E. Phillips, Ann. S. Afr. Mus. 16(2): 116. 1925.
Vernonia flanaganii (E. Phillips) Hilliard, Notes Roy. Bot. Gard. Edinburgh 42(2): 238. 1985.

##### Note.

Distinguished as a variety from typical *Vernonia hirsuta* DC. by Phillips (1925) by the narrow, not cordate, bases of the lower leaves and the long-acuminate tips of the involucral bracts that equal or exceed the pappus.

##### Distribution.

South Africa (Natal).

#### Hilliardiella hirsuta

Taxon classificationPlantaeAsteralesAsteraceae

(DC.) H. Rob., 1999

Vernonia hirsuta (DC.) Sch. Bip. ex Walp., Rep. 2:947. 1843.
Vernonia hirsuta (DC.) Sch. Bip. ex Walp. var. obtusifolia Harv. Flora Capensis 3: 52. 1864.
Hilliardiella hirsuta (DC.) H. Rob., Proc. Biol. Soc. Wash. 112(1): 230. 1999.

##### Distribution.

Lesotho, South Africa (Transvaal, Orange Free State, Natal, Cape colony), and Swaziland.

#### Hilliardiella nudicaulis

Taxon classificationPlantaeAsteralesAsteraceae

(DC.) H. Rob., 1999

Webbia nudicaulis DC., Prodr. 5:73. 1836
Vernonia dregeana Sch. Bip. ex Walp., Rep. 2: 947. 1843.
Hilliardiella nudicaulis (DC.) H. Rob., Proc. Biol. Soc. Wash. 112(1): 230. 1999.

##### Distribution.

South Africa (Cape colony, Natal, Transvaal).

#### Hilliardiella oligocephala

Taxon classificationPlantaeAsteralesAsteraceae

(DC.) H. Rob., 1999

Webbia oligocephala DC., Prodr. 5: 73. 1836.
Webbia elaegnoides DC., Prodr. 5: 73. 1836, non *Vernonia elaeagnoides* Kunth in H.B.K.
Vernonia elaeagnoides (DC.) Sch. Bip. ex Walp., Rep. 2: 947. 1843.
Vernonia krausii Sch. Bip. ex Walp., Rep. 2: 947. 1843.
Hilliardiella oligocephala (DC.) H. Rob., Proc. Biol. Soc. Wash. 112(1): 230. 1999.

##### Distribution.

Tanzania south to South Africa (Transvaal, Orange Free State, Natal, Cape colony), Botswana, Lesotho and Swaziland.

#### Hilliardiella pseudonatalensis

Taxon classificationPlantaeAsteralesAsteraceae

(Wild) H. Rob., Skvarla & V.A. Funk comb. nov.

urn:lsid:ipni.org:names:77152897-1

Vernonia pseudonatalensis Wild, Kirkia 11: 11. 1978.

##### Distribution.

Mozambique, South Africa (Transvaal), and Swaziland.

#### Hilliardiella sutherlandii

Taxon classificationPlantaeAsteralesAsteraceae

(Harv. in Harv. & Sond.) H. Rob., 2005

Vernonia sutherlandii Harv. in Harv. & Sond., Fl. Cap. 3: 52. 1865.
Hilliardiella sutherlandii (Harv. in Harv. & Sond.) H. Rob., Phytologia 87: 82. 2005.

##### Distribution.

South Africa (Natal, Transvaal) and Swaziland.

#### Linzia

Taxon classificationPlantaeAsteralesAsteraceae

Sch. Bip. ex Walp., 1843

**Figure 15.**
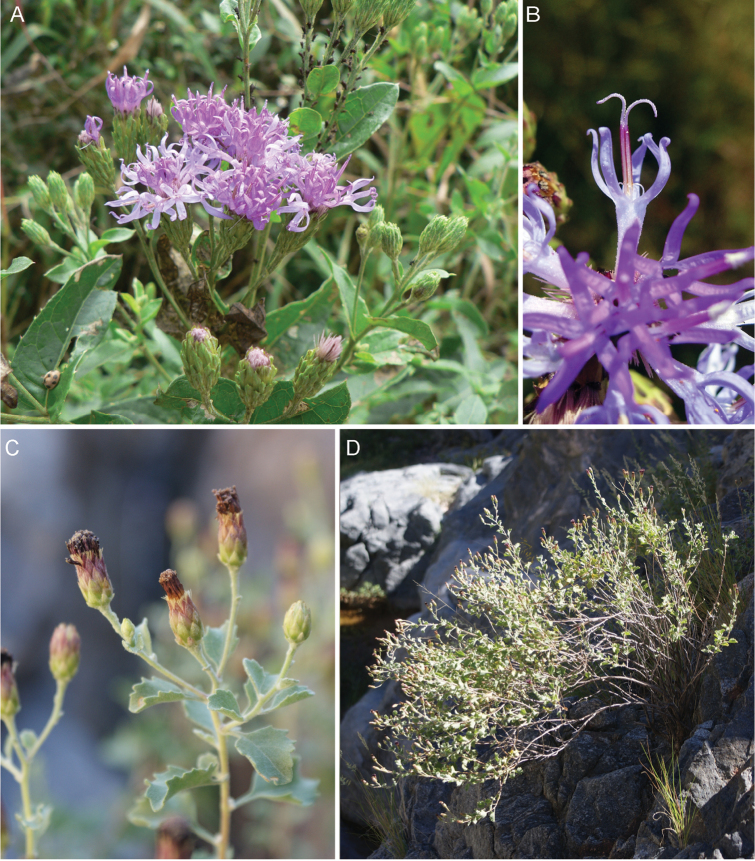
Photographs of *Linzia* and *Namibithamnus*: **A–B** Linzia glabra Steetz in Peters, note that the flowers bloom in two groups with the outer ones blooming first (light colored in 15B) followed by the innermost ones (dark purple in 15B) **C–D** *Namibithamnus obionifolius* (O. Hoffm.) H. Rob. See Appendix C for citation details.

**Figure 16.**
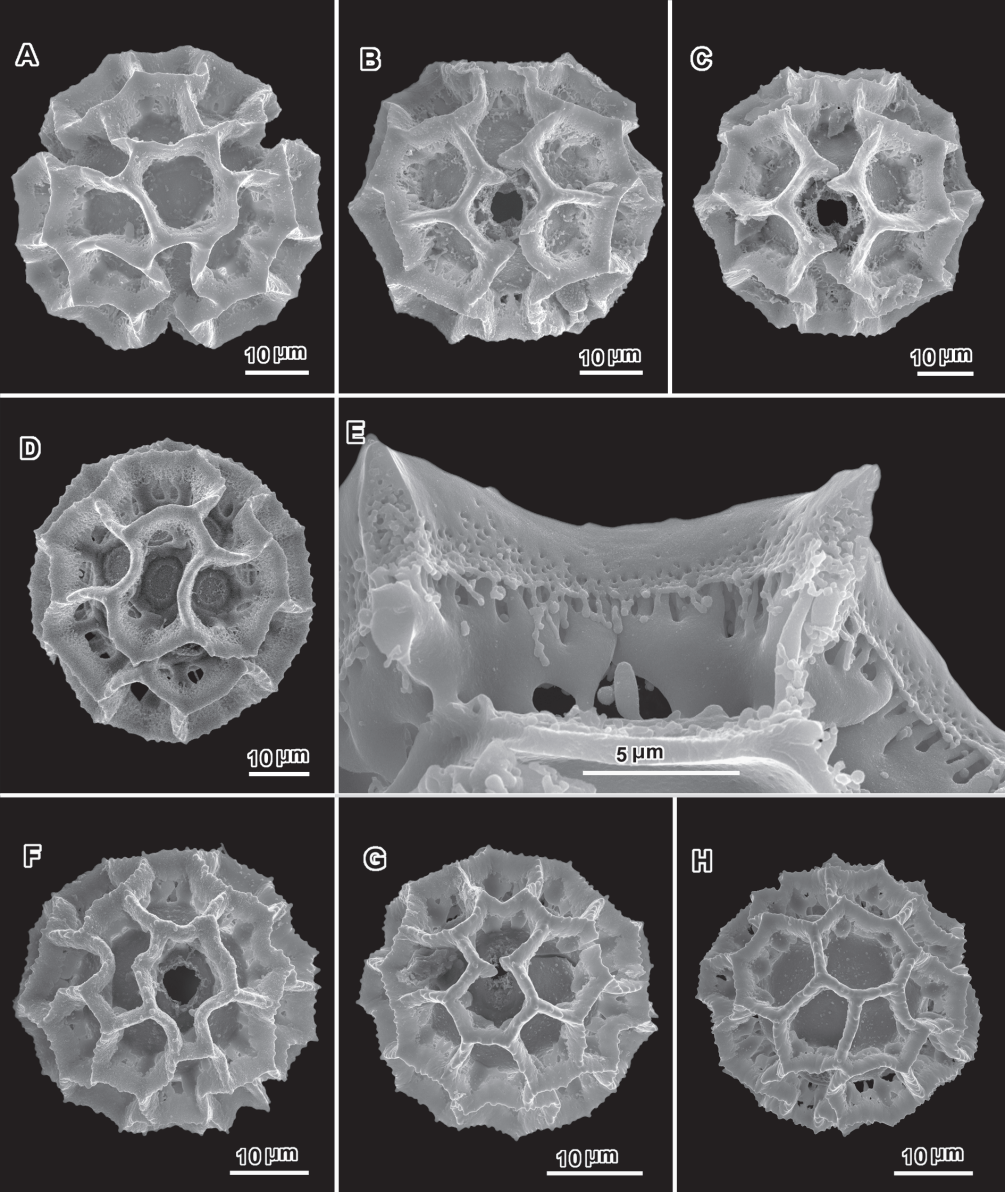
Scanning electron micrographs of *Linzia* and *Namibithamnus* pollen. **A, B, E** *Linzia rosenii*. (R.E. Fries) H. Rob., Skvarla & V.A. Funk. **A** Polar view **B** Equatorial view **C** Equatorial view. Note differences of lophae surrounding pores in **B** and **C, D** *Linzia glabra* Steetz in Peters. Lateral view. **E** *Linzia rosenii*. Fractured pollen wall. **F–H** *Namibithamnus obionifolius* (O. Hoffm.) H. Rob., Skvarla & V.A. Funk. **F** Equatorial view. This is the most common form of aperture **G** Equatorial view. Less common form of aperture with broken lophal arms apparent **H** Near polar view (**A–C, E** *Jacobsen 3075* **D** *West 7292* **F-H** *Tölken & Hardy 770*).

Figures 13 B ; 15 A–B ; 16 A–E

Linzia Sch. Bip. ex Walp., Rep. 2: 948. 1843. – Type: *Linzia vernonioides* Sch. Bip. ex Walp.
Vernonia sect. Azurae S.B. Jones, Rhodora 83: 74. 1981. – Type: *Linzia glabra* Steetz in Peters.

##### Descriptions.

Perennial herbs; stems with simple multiseptate hairs. Leaves alternate, subsessile to short-petiolate. Inflorescence corymbiform cymes or single heads with short to long peduncles. Involucre funnelform to campanulate; bracts 50–150 in 5–6 series, often pectinate-denticulate with spicules along lateral margins, outer tips often elongate, green and recurved; receptacle epaleaceous. Florets ca. 20–50 in a head; cortollas bluish, tube very long, funnelform near throat; throat very short, lobes apically stiffly pilosulous; anther base rounded; apical appendage glabrous, triangular with thickened ornamentation in center; style base with small annuliform node. Achenes strongly 10-costate, usually with rows of idioblasts or specialized cells along sides of costae, surface setuliferous, setulae slender with pairs of cells not or scarcely separated at tip, raphids subquadrate to short-oblong; pappus of many somewhat persistent long bristles, with outer series short. Chromosome number n = 10 (Jones 1979, 1982).

Pollen tricolporate, psilolophate, with spur muri intruding into short colpi above and below pore, single polar lacunae often present, not echinate, with or without micropunctations resticted to muri. Muri showing baculae with broadened base, branching distally into many bacula-like branches (Fig. 16 E), a form that seems almost transitional to a rhizomate condition.

Most notable secondary metabolites, sesquiterpene germacranolides, elemanolides (Bohlman and Jakupoic 1990, as *Vernonia glabra* Vatke & *Vernonia melleri* Oliv. & Hiern).

#### Key to the species of *Linzia*

**Table:** 

1	Capitula usually numerous in terminal corymbiform or thyrsiform cymes, peduncles up to 2–3 times as long as the involucre; plants with rather equally leafy stems	***Linzia glabra***
–	Capitula on long peduncles, 1 or few in open terminal cymes, peduncles mostly 5 or more times as long as the involucre	**2**
2	Leaves in rosettes, arising from a root-crown; capitula to 2.5–3.0 cm high or wide	***Linzia gerberiformis***
–	Slender leaves on short branches, arising from creeping rhizome; capitula mostly 1.2–1.6 cm high or wide	***Linzia rosenii***

#### Linzia gerberiformis

Taxon classificationPlantaeAsteralesAsteraceae

(Oliv. & Hiern in Oliv.) H. Rob., 1999

Vernonia gerberiformis Oliv. & Hiern in Oliv., Fl. Trop. Afr. 3: 285. 1877.
Vernonia collina Schlechter, J. Bot. 1898: 374. 1898?
Vernonia gerberiformis var. hockii (De Wild. & Muschl.) G.V. Pope, Kew Bull. 43(2): 280. 1988. Distribution: Lesotho, South Africa (Transvaal, Orange Free State, Natal, Cape colony).
Vernonia gerberiformis subsp. macrocyanus (O. Hoffm.) C. Jeffrey, Kew Bull. 43: 234. 1988.
Vernonia primulina O. Hoffm. in Warburg, Kunene-Sambesi Exped. 402. 1903.
Vernonia pristis Hutch. & Burtt, Rev. Zool. & Bot. Afr. 23: 38. 1932.
Linzia gerberiformis (Oliv. & Hiern in Oliv.) H. Rob., Proc. Biol. Soc. Wash. 112(1): 237. 1999.
Linzia gerberiformis subsp. macrocyanus (O. Hoffm.) Isawumi, Comp. Newsl. 40: 38. 2008.

##### Distribution.

Angola, Burundi, Cameroon, Congo, Malawi, Nigeria, Sudan, Tanzania, Uganda, Zambia, Zimbabwe.

#### Linzia glabra

Taxon classificationPlantaeAsteralesAsteraceae

Steetz in Peters, 1864

Vernonia glabra (Steetz) Vatke, Oesterr. Bot. Zeitschr. 27: 194. 1877.
Linzia glabra Steetz in Peters, Reise Mossamb. Bot. 353. 1864.
Vernonia obconica Oliv. & Hiern in Oliv., Fl. Trop. Afr. 3: 286. 1877.
Vernonia ondongensis Klatt ex Schinz, Bull. Herb. Boiss. 3: 430. 1895.
Vernonia glabra (Steetz) Vatke var. laxa (Steetz) Brenan, Mem. N. Y. Bot. Gard. 8(5): 460. 1954.

##### Distribution.

Burundi, Congo, Kenya, Tanzania, Madagascar, south to Angola, Mozambique, and Namibia, South Africa (Transvaal, Natal) and Swaziland.

#### Linzia rosenii

Taxon classificationPlantaeAsteralesAsteraceae

(R.E. Fries) H. Rob., Skvarla & V.A. Funk comb. nov.

urn:lsid:ipni.org:names:77152903-1

Vernonia rosenii R. E. Fries, Wiss. Ergebn. Schwed. Rhodesia-Congo Exped. 1911–1912, 1: 323. 1916.

##### Distribution.

Botswana.

#### Namibithamnus

Taxon classificationPlantaeAsteralesAsteraceae

H. Rob., Skvarla & V.A. Funk gen. nov.

urn:lsid:ipni.org:names:77152892-1

Figures 15 C–D ; 16 F–H

##### Type.

*Vernonia obionifolia* O. Hoffm.

##### Descriptions.

Small aromatic shrubs to 1.5 m tall; stems, leaves, involucral bracts densely yellowish gray tomentellous or sericeous with crowded T-shaped hairs, hairs with slender 0–2-septate short stalks and small naviculiform or rather elongate cap-cells. Leaves alternate, short-petiolate, with small axillary fascicles usually present, more crowded proximally, smaller distally; blades 5–12 mm long, oblong to obovate, with undulate entire to coarsely dentate margins, basal pair of secondary veins scarcely evident or evident and strongly ascending, minute glandular dots densely disposed on both surfaces. Inflorescences appearing shortly scapose, with numerous pedunculate heads in a corymbiform or partly subumbellate arrangement. Heads campanulate, 6–7 mm wide and high; involucral bracts ca. 60 in ca. 6 strongly gradate series, persistent, oblong ovate with narrow apiculate tips, yellowish with reddish patch or midvein below tip, margins entire, broadly and distinctly thick and pale; receptacle convex, pitted with broad pale network of ridges. Florets 35–40 in a head. Corollas purple, narrowly funnel-shaped from a slender basal tube; throat twice as long as the erect, linear lobes, outer surface of base and throat mostly glabrous, lobes densely glandular-dotted; anther thecae narrow, slightly longer than throat, bases without tails, apical appendages shortly oblong-triangular, glabrous, with thin cell walls; style base with narrow annuliform node; with acicular sweeping hairs almost completely restricted to style branches, a few at top of shaft. Achenes 5-costate, with setulae not divided at tips, surfaces with numerous ungrouped idioblasts, raphids elongate; carpopodium turbinate, glabrous; pappus of ca. 35 slender persistent bristles, bristles as wide at tips as at base, densely scabrid on margins and outer surface, outer series of distinct, smooth, lanceolate scales. Chromosome number unknown.

Pollen ca. 45 μm in diam. in fluid, lophate, triporate, not echinate, perforated tectum restricted or lacking, crests of muri sparsely papillose (Fig. 16 F–H).

#### Key to the species of *Namibithamnus*

**Table:** 

1	Leaves oblong, unlobed to few-lobed on lateral margins; secondary veins obscure; tomentellous with crowded minute trichomes bearing minute naviculiform cap-cells	***Namibithamnus obionifolius***
–	Leaves obovate, with numerous lobes distally; acending secondary veins evident; trichomes appearing sericeous, with elongate cap-cells	***Namibithamnus dentatus***

#### Namibithamnus obionifolius

Taxon classificationPlantaeAsteralesAsteraceae

(O. Hoffm.) H. Rob., Skvarla & V.A. Funk comb. nov.

urn:lsid:ipni.org:names:77152904-1

Vernonia obionifolia O. Hoffm., Bot. Jahrb. Syst. 10: 272. 1888.

##### Note.

With habit remarkably like *Orbivestus cinerascens*, and often in herbaria identified as this species. Differs clearly by non-seriate cymose inflorescence, thicker pale margins on involucral bracts, thicker tips on pappus bristles and lophate/triporate pollen. The margins of the involucral bracts are similar to those of *Erlangea* and *Bothriocline*.

##### Distribution.

Namibia.

#### Namibithamnus dentatus

Taxon classificationPlantaeAsteralesAsteraceae

(Merxm.) H. Rob., Skvarla & V.A. Funk comb. et stat. nov.

urn:lsid:ipni.org:names:77152905-1

Vernonia obionifolia O. Hoffm. ssp. dentata Merxm., Mitt. Bot. Staatssamml. München 3: 608. 1960.

##### Note.

Thoroughly distinct in appearence, having larger more lobed leaves indicative of more moist habitats.

##### Distribution.

Namibia.

#### Oocephala

Taxon classificationPlantaeAsteralesAsteraceae

(S.B. Jones) H. Rob., 1999

**Figure 17.**
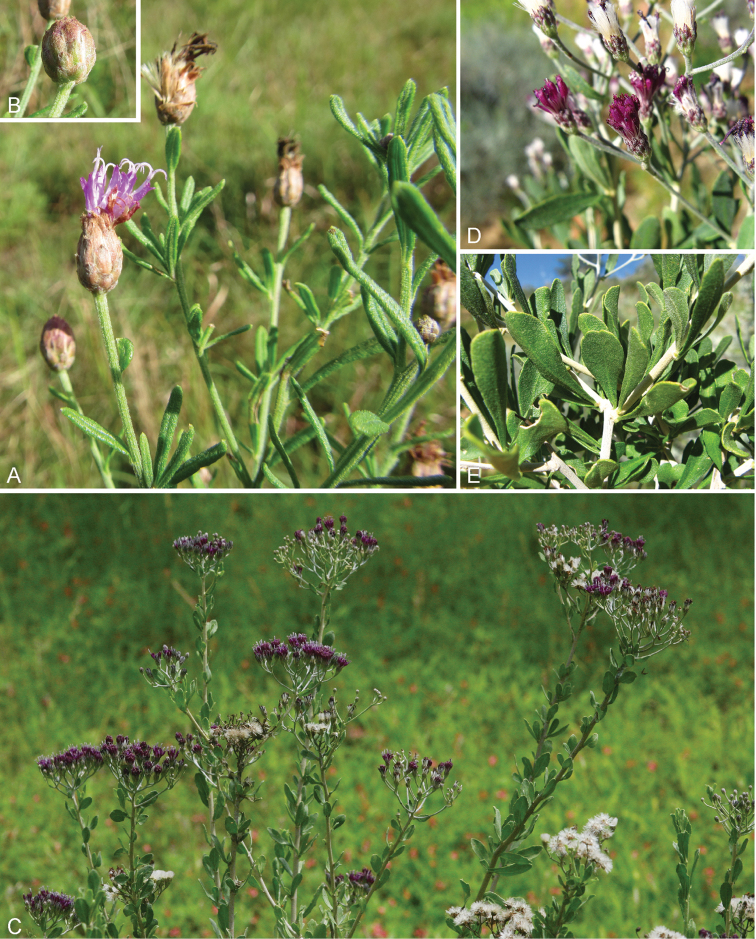
Photographs of *Oocephala* and *Orbivestus*: **A–B** *Oocephala centauroides* (Klatt) H. Rob. & Skvarla, note: egg-shaped head **C–E** *Orbivestus cinerascens* (Sch. Bip. in Schweinf.) H. Rob. See Appendix C for citation details.

**Figure 18.**
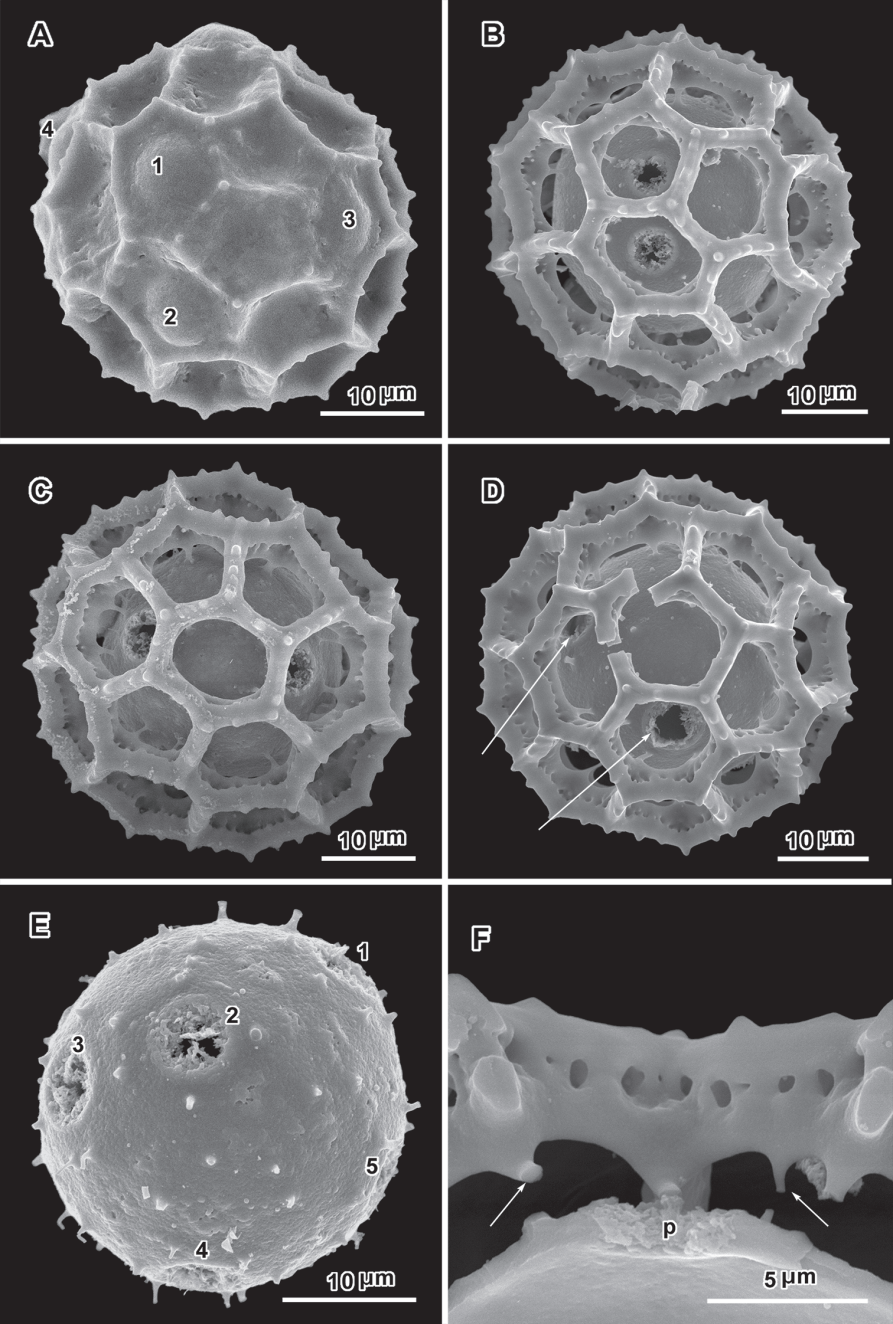
Scanning electron micrographs of *Oocephala staehelinoides* (Harv.) H. Rob. & Skvarla. **A** Unacetylized grain showing three pores with caps intact, two pores in adjacent lacunae **B–D** Intact or nearly intact grains showing pores in both pentagonal and hexagonal lacunae **B** with pores in adjacent lacunae **E** Grain stripped of muri showing five pores and stubs of muri attachments **F** Segment of muri showing rhizomate structure and remnants of weak basal attachments to footlayer, (p) pore. **A–F** are from collection of Liebenberg 8843.

Figures 17 A–B ; 18 A–F

Oocephala (S.B. Jones) H. Rob., Proc. Biol. Soc. Wash. 112(1): 230. 1999. Type species: *Vernonia oocephala* Baker
Vernonia subsect. Oocephalae S.B. Jones, Rhodora 83: 72. 1981.

##### Descriptions.

Low, much-branched shrubs to 1 m high, stems with L-shaped hairs on multiseptate stalks, cap-cells one-armed. Leaves alternate, sub-sessile, linear to elliptical, sometimes serrate. Inflorescences corymbiform cymes, with usually shortly pedunculate heads or with heads sessile in apical clusters of leaves. Involucre ovoid or cylindrical; bracts 20–40 in 4–7 gradate series, ovate to oblong, appressed; receptacle without pales. Florets ca. 10–15 in a head; corollas white or lavender, tubular to narrowly funnelform, throat as long as lobes, tips without hairs or with few short biseriate hairs; anther bases rounded, apical appendages glabrous, with thin-walled cells; style base with narrow ring; style branches with acicular sweeping hairs. Achenes weakly 8-ribbed, sericeous with many setulae, idioblasts numerous, raphids narrowly elongate; pappus biseriate, outer shorter and broader, inner setiform, subplumose, glabrous near base. Chromosome number unknown.

Pollen 7–8-porate, with pores scattered over the whole surface in lacunae that are usually not adjacent, lophate (Fig. 18 A–F), minutely papillose on muri, nonperforated tectum restricted to muri, emicropunctate, baculae regularly spaced in single row under muri or lophae, baculae subtended by “rhizomate” structure that is as broad as the outer layer, and gives the muri or lophae a two-layed structure with small evenly spaced columellae separating the layers. The rhizomiform base of the muri is weakly attached to the footlayer thus causing muri to easily detach from the core of the grain. Lacunae of exine of pentagons mixed with hexagons. The structure of the bucky ball was a remarkable close approximation of the structure of the pollen. It was study of the toy ball that led to the conclusion that the pollen characteristically had seven or eight pores. Other pollen grains show a somewhat different pattern of pores, where pores occur in pairs, one each in a pair of adjacent lacunae (Fig. 18 B). The polyporate, subspherically symmetrical, rhizomate form of pollen in *Oocephala* is shared in a somewhat less symmetrical form by *Polydora*, but as far is currently known, these *Oocephala* and *Polydora* grains, with their non-equatorial pores, are unique in the Asteraceae (Robinson and Skvarla 2014).

Notable secondary metabolites: sesquiterpene glaucolides (Bohlmann and Jakupovic 1990 as *Vernonia staeheleinoides* Harv.).

#### Key to the species of *Oocephala*

**Table:** 

1	Stems and peduncles sparsely hispid with short spreading hairs; involucre 6–7 mm wide; involucral bracts with mucronate tip	***Oocephala centaureoides***
–	Stems and peduncles subcanescent with appressed hairs; involucre 3–4 mm wide; involucral bracts with obtuse or rounded tips	***Oocephala staehelinoides***

#### Oocephala centaureoides

Taxon classificationPlantaeAsteralesAsteraceae

(Klatt) H. Rob. & Skvarla, 2014

Vernonia centaureoides Klatt, Bull. Herb. Boiss. 4: 824. 1896.
Vernonia schlechteri O. Hoffm., Bot. Jahrb. Syst. 24: 466. 1897.
Oocephala centaureoides (Klatt) H. Rob. & Skvarla, Phytokeys 38: 2. 2014.

##### Distribution.

South Africa (Transvaal, Natal), and Swaziland.

#### Oocephala staehelinoides

Taxon classificationPlantaeAsteralesAsteraceae

(Harv.) H. Rob. & Skvarla, 2014

Vernonia staehelinoides Harv., Thes. Cap. 2: 36. 1863.
Oocephala staehelinoides (Harv.) H. Rob. & Skvarla, Phytokeys 38: 2. 2014.

##### Distribution.

South Africa (Transvaal), and Swaziland.

#### Orbivestus

Taxon classificationPlantaeAsteralesAsteraceae

H. Rob., 1999

**Figure 19.**
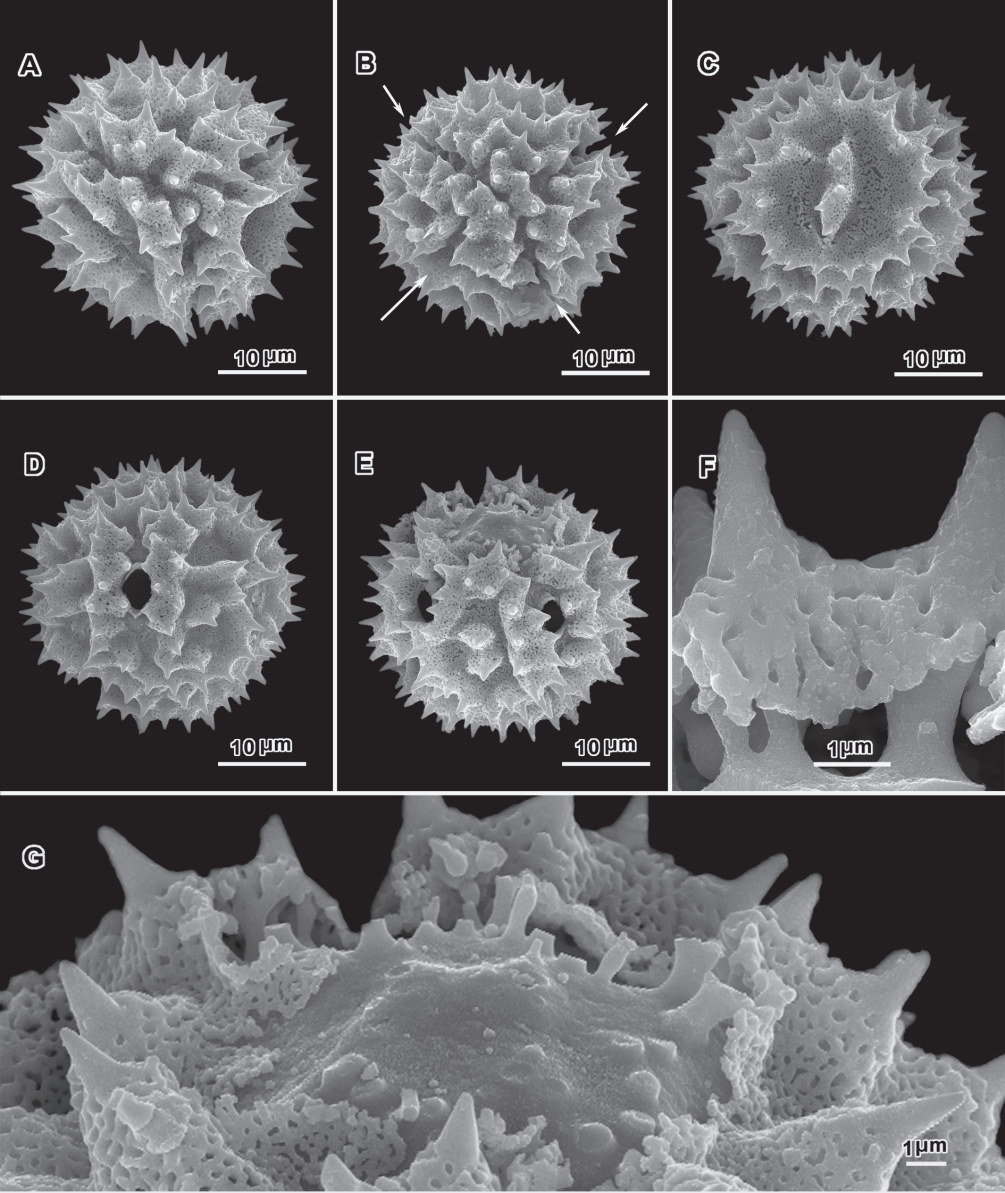
Scanning electron micrographs of *Orvibestus cinerascens* (Sch. Bip. in Schweinf.) H. Rob. **A** Polar view showing 3 colpi **B** Polar view showing 4 colpi **C** Lateral view showing highly perforated sheet-like layers of exine common in many grains of the sample **D** Equatorial view (appearing nearly echinolophate) **E** Lateral/equatorial view showing 2 pores **F** Fractured grain showing thickened columellae supporting a higher perforate surface **G** Fractured polar surface (**A–G** *Koekemoer 232*).

Figures 17 C–E ; 19 A–G

Orbivestus H. Rob., Proc. Biol. Soc. Wash. 112(1): 230. 1999. – Type: *Vernonia karaguensis* Oliv. & Hiern.
Vernonia subg. Orbisvestus S.B. Jones, Rhodora 83: 60. 1981. – Type: *Vernonia karaguensis* Oliv. & Hiern.

##### Descriptions.

Subshrubs to small shrubs with erect stems from a woody base, not or sparsely branched between base and inflorescence; hairs T-shaped. Leaves alternate, usually decrescent upwardly, sessile or short petiolate, blades elliptical or ovate to oblanceolate, mostly 4–9 cm long, 2–5 cm wide, base short-obtuse to acuminate, margins scarcely repand-dentate, apex short-acute, upper surface with small spinules and few small hairs, lower surface paler, grayish with slender hairs and partially sunken glandular dots; venation pinnate, with up to six or eight lateral veins each side, spreading at 45–60º angles. Inflorescences with leaves of main axis only somewhat to greatly reduced, with only minute bracteoles on branches. Inflorescence shape broadly corymbiform or cylindrical with rounded to flattened top, with lower heads appearing sessile as result of proliferation by immediately subtending branches forming seriate or scorpioid cymes, branches of inflorescence tomentose with T-shaped hairs. Heads broadly campanulate, 4–14 mm high and wide; involucral bracts mostly persistent, innermost somewhat deciduous, ca. 50–100 in 5–7 series, strongly gradate, 1–8 mm long, 1.0–1.5 mm wide, ovate to oblong, subacute and mucronate to apiculate at tip, innermost acute, tips appressed, margins membraneous and irregularly denticulate distally, often reddish, with dark median keel extending to apex, scarcely thickened and greenish near keel, with numerous small T-shaped hairs except at margins. Receptacle epaleate and tuberculate. Florets 15–ca. 50 in a head; corollas purplish, narrowly funnelform, 4–8 mm long, with sparsely scattered glandular dots, tube slender, 2–3 mm long, throat 1.5–2.5 mm long, lobes 1.0–2.5 mm long, linear-lanceolate, erect, not recurving, sparsely glanduliferous to distinctly or minutely scabridulous outside, without longitudinal internal ducts filling lobe; anther thecae 1–2 mm long, without glandular dots, calcarate and with long tails at base, endothecial cells short usually with 2–3 nodes on transverse walls; apical appendage 0.5–1.0 mm long, narrowly lanceolate, often sharply acute; style base with distinct expanded node; sweeping hairs on style branches and scarcely extending on to upper style shaft, slender and narrowly acute. Achenes 1.5–2.0 mm long when mature, 5-costate, with few to many setulae when young, often glabrous at maturity, often with numerous glandular dots on sides between costae, surface with numerous idioblasts that are not joined in series, with narrowly rhomboid raphids internally; carpopodium stopper-shaped to slightly turbinate, with many series of small thick-walled cells; inner pappus of 25–30 slender capillary bristles, rather flattened outside and barbellate on sides, tips only slightly narrowed, outer pappus of narrow scales 0.5–1.5 mm long. Chromosome numbers n = 10, 18, 20 (Mangenot and Mangenot 1962; Bhandari and Singhvi 1977; Morton 1993).

Pollen grains ca. 50 μm in diameter in fluid, type A, sublophate, tricolporate, echinate, with perforated tectum continuous between colpi (Fig. 19 D–E). The grains may also be somewhat asymmetrical (Fig. 19 A).

Most notable secondary metabolites are 5-alkylcomumarins (Bohlmann and Jakupovic 1990, as *Vernoniia cinarescens* Sch. Bip.) and sesquiterpene glaucolides (Bohlmann and Jakupovic 1990, as *Vernonia cistifolia* O. Hoffm.).

The genus is almost alone in the eastern hemisphere in its seriate cymes, often referred to as scorpioid cymes. Such inflorescences are common in the western hemisphere Vernonieae, occurring in *Vernonia* itself.

#### Single species in the flora area

##### Orbivestus cinerascens

Taxon classificationPlantaeAsteralesAsteraceae

(Sch. Bip.) H. Rob., 1999

Vernonia cinerascens Sch. Bip. in Schweinf., Beitr. Fl. Aeth. 162. 1897.
Vernonia tephrodioides Chiov., Fl. Somal. 2: 255. 1932.
Vernonia luederitziana O. Hoffm., Bolet. Soc. Soc. Brot. 10: 171. 1892.
Vernonia porta-taurinae Dinter ex Merxm., Mitt. Bot. München 2: 38. 1954, nom. nud. in syn.
Vernonia squarrosa Dinter ex Merxm., Mitt. Bot. München 2: 38. 1954, nom. nud. in syn.
Orbivestus cinerascens (Sch. Bip.) H. Rob., Proc. Biol. Soc. Wash. 112(1): 230. 1999.

###### Distribution.

In Africa in Angola, Botswana, Kenya, Tanzania, Senegal, Uganda, and Zimbabwe; also in western India.

#### Parapolydora

Taxon classificationPlantaeAsteralesAsteraceae

H. Rob., 2005

**Figure 20.**
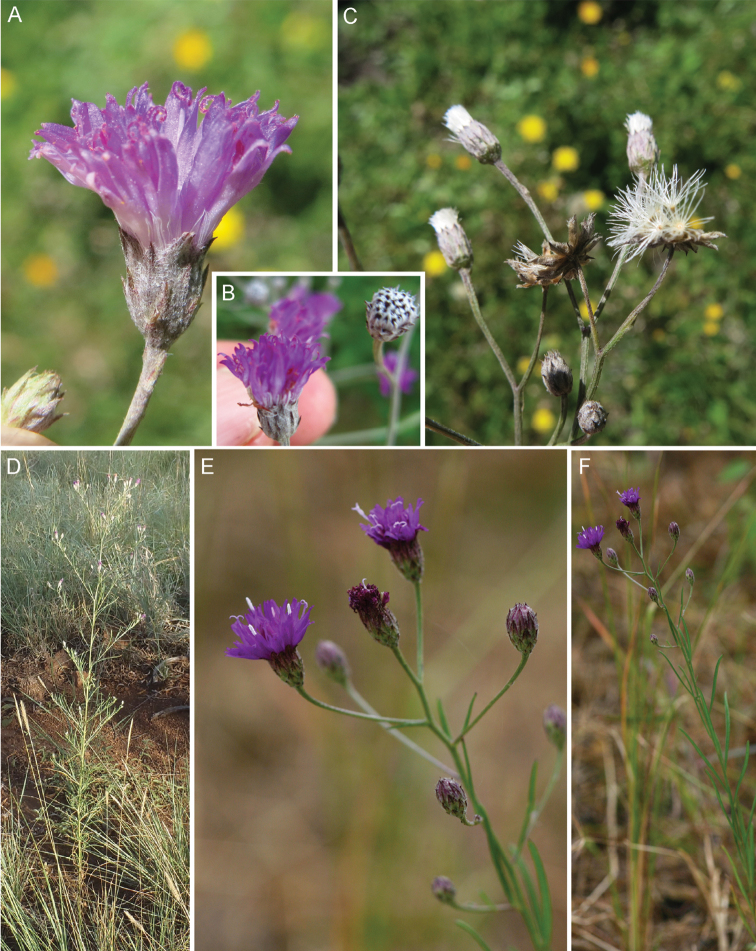
Photographs of *Parapolydora* and *Polydora*: **A–C** *Parapolydora fastigiata* (Oliv. & Hiern in Oliv.) H. Rob. **D–F** *Polydora poskeana* (Vatke & Hildebrandt) H. Rob. See Appendix C for citation details.

**Figure 21.**
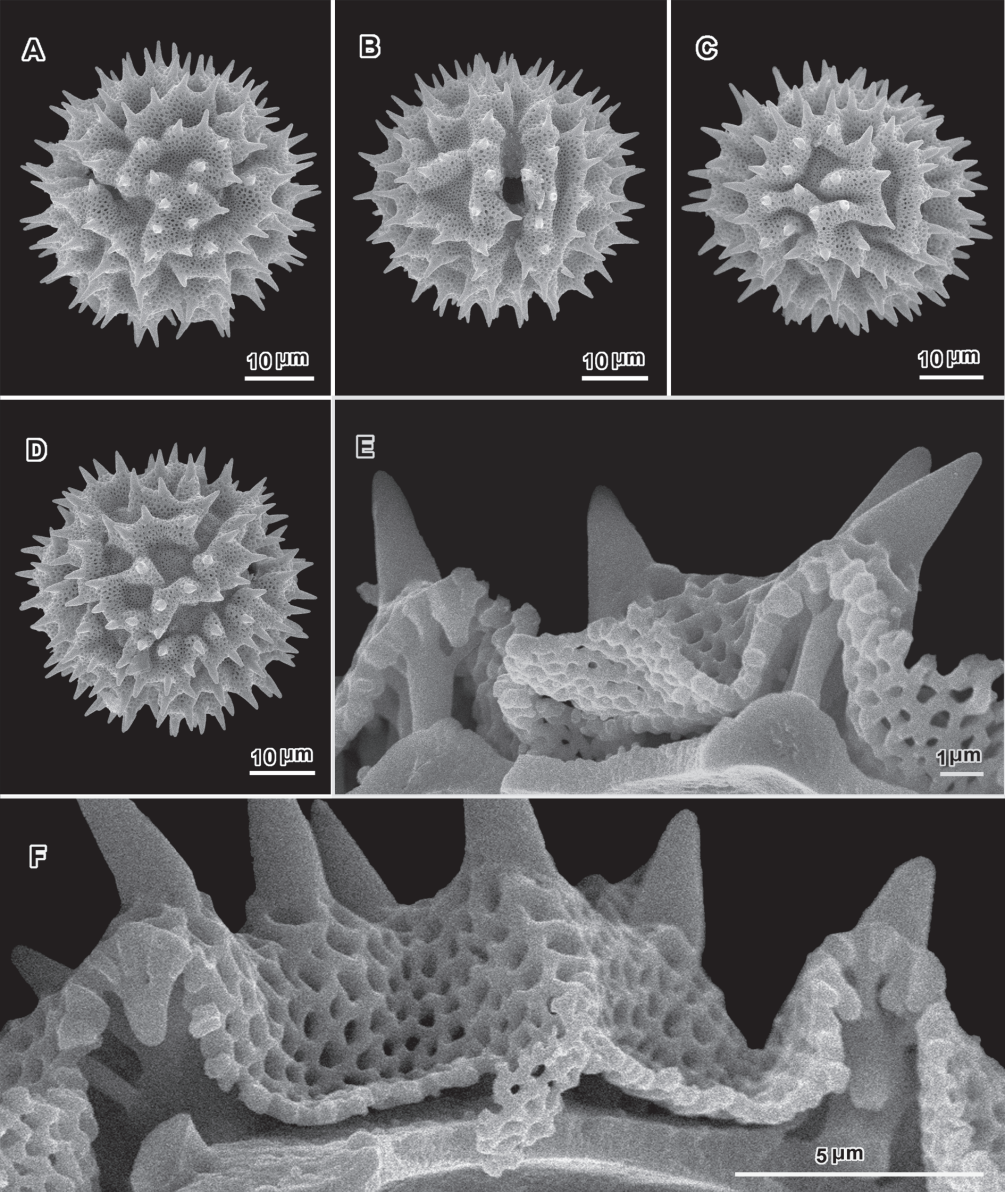
Scanning electron micrographs of acetolyzed sublophate-echinolophate pollen grains of two collections of *Parapolydora fastigata* (Oliv. & Hiern in Oliv.) H. Rob. **A** Polar view **B** Equatorial view showing thickened echinolophate ridges along aperture **C–D** Lateral views showing highly perforate meandering lophal ridges **E** Grain fragment showing thickened columellae underneath two spine regions **F** Grain fragment showing perforate lacunar exine with close parallel proximity to foot layer between thickened columellae supporting spines. (**A** *Pienaar 1073* **B–F** *Seydel 4023*).

Figures 13 C ; 20 A–C ; 21 A–F

Parapolydora H. Rob., Phytologia 87(2): 78. 2005. – Type: *Vernonia fastigiata* Oliv. & Hiern.

##### Descriptions.

Perennial herbs 0.2–1.0 m tall; from slender prostrate or creeping stem or rhizome, erect stems with few to many ascending branches, five-ribbed, sides with numerous glandular dots, glabrous or finely and sparsely puberulous with some simple multiseptate hairs, and some one-armed L-shaped hairs with stalk near one end as in *Polydora*. Leaves alternate, linear to narrowly elliptic-lanceolate, venation pinnate with short, ascending, secondary veins weakly prominulous below, surfaces concolorous, glandular dots more numerous below, sparsely puberulous. Inflorescences of long-pedunculate heads terminal on leafy stems and branches; involucres broadly campanulate to subglobose; involucral bracts 110–130 in ca. six series, persistent, gradate, from 2–12 mm long, bases of bracts oblong, pale, appressed, covered with dense pale tomentum, bracts distally constricted into long glabrous, often reflexed awn, darkened along costa near base of awn; receptacle epaleaceous, alveolate. Florets 45–50 in a head; corollas lavender, without hairs, basal tubes narrowly funnelform, glabrous, throats about as long as linear lobes, few glands on throat and glands clustered at lobe tips; thecae of anthers without tails at base; apical appendages ovate-lanceolate, glabrous, with thin-walled cells; style base with distinct annular node; style branches with long acicular sweeping hairs scarcely extending below base of branches. Achenes weakly 8–10-veined, with setulae becoming long and uniseriate from near middle or near base, rarely with one long cell and one short cell, idioblasts numerous from base to top of achene, raphids elongate; pappus white or grayish, inner series of many barbellate bristles, not broadened at tips, outer series of numerous, short, linear scales. Chromosome number unknown.

Pollen ca. 50 μm in diam., sublophate, echinate (Type A), tricolporate, sub-echinolophate (Fig. 21 A–D).

Most notable secondary metabolites, sesquiterpene nerolidol derivatives (Bohlmann and Jakupovic (1990).

#### Key to the species of *Parapolydora*

**Table:** 

1	Stems, abaxial surfaces of leaves and peduncles with whitish puberulence; pale margins of involucral bracts usually without scarious border, rather evenly tapering into base of awn; achenes hispid with short spreading setulae	***Parapolydora fastigiata***
–	Stems, abaxial surfaces of leaves and peduncles without whitish hairs; pale margins of involucral bracts usually with expanded scarious border, mostly not evenly tapering into base of awn; achenes sericeous with long setulae	***Parapolydora gerrardii***

#### Parapolydora fastigiata

Taxon classificationPlantaeAsteralesAsteraceae

(Oliv. & Hiern) H. Rob., 2005

Vernonia fastigiata Oliv. & Hiern in Oliv., Fl. Trop. Africa 3: 282. 1877.
Vernonia schinzii O. Hoffm. ex Schinz, Bull. Herb. Boiss. 1: 72. 1893. (= *Erlangea misera* (Oliv. & Hiern) S. Moore, according to Wild & Pope 1977).
Parapolydora fastigiata (Oliv. & Hiern) H. Rob., Phytologia 87(2): 79. 2005.

##### Distribution.

Namibia, South Africa (Transvaal), and Zimbabwe.

#### Parapolydora gerrardii

Taxon classificationPlantaeAsteralesAsteraceae

(Harv.) H. Rob., Skvarla & V.A. Funk comb. nov.

urn:lsid:ipni.org:names:77152898-1

Vernonia gerrardii Harv.,Thes. Cap. 2: 36, t. 157. 1863.

##### Distribution.

South Africa (Natal).

#### Polydora

Taxon classificationPlantaeAsteralesAsteraceae

Fenzl, 1844

**Figure 22.**
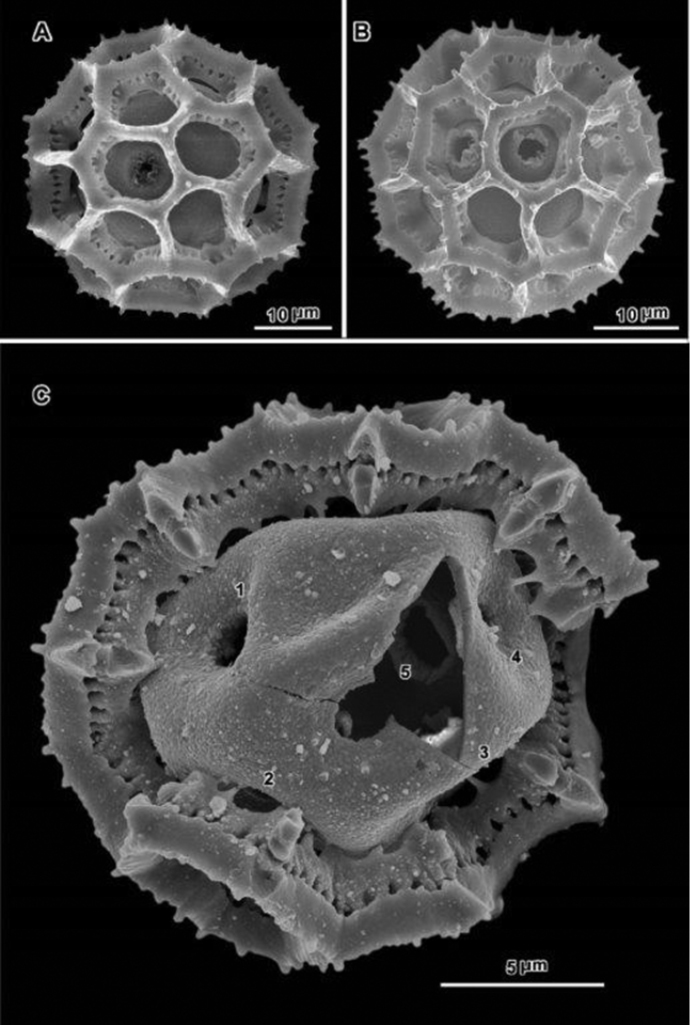
Scanning electron micrographs of *Polydora angustifolia* (Steetz) H, Rob. **A** Intact grain with visible pore **B** Intact grain with 2 visible pores in adjacent lacunae **C** Grain with muri partially removed showing distorted inner surface and five pores, one pore on opposite surface visible through torn area. **A** from *Brass 16090* **B, C** from *Christensen* & *Patel 1457*. Views from Robinson & Skvarla, PhytoKeys 2014.

Figures 20 C–F ; Fig. 22 A–C

Polydora Fenzl, Flora 27: 312. 1844. – Type: *Polydora stoechadifolia* Fenzl = *Webbia serratuloides* DC.
Crystallopollen Steetz in Peters, Reise Mossamb. Bot. 363. 1864. – Type: *Crystallopollen angustifolium* Steetz

##### Resources.

Some species of the genus are treated by Pope (1986).

##### Descriptions.

Mostly annuals; stems with L-shaped hairs bearing elongate one-armed cap-cells. Leaves alternate. Inflorescence a thyrsoid panicle with corymbiform cymose branches bearing pedunculate heads or a single terminal head. Involucral bracts ca. 80 in ca, seven series, often with widely scarious margins and awns often black at tips; receptacles epaleaceous. Florets ca. 30 in a head; corollas whitish to purplish, basal tube long, narrowly funnelform distally, throat as long as the narrow glabrous lobes; anther bases plain, not tailed; apical appendage glabrous, with thin cell walls, sometimes weakly ornamented; style base with distinct annular node; branches with acicular sweeping hairs. Achenes 5 or 8–10-ribbed, setuliferous with setulae scarcely divided at tips, idioblasts present but not grouped, raphids elongate; pappus with copious barbellate setae, greenish, yellowish or tawny, rarely white, outer pappus short, squamiform. Chromosome number n = 9, 10 (Jones 1979, 1982, Ayodele 1999).

Pollen lophate with ca. 32 lacunae, with five or more pores that seem to be rather asymmetrically distributed on the grains; the pores occur in lacunae that, in a few cases, are adjacent; margins of muri minutely echinate to psilate, without micropunctations, baculae closely spaced in single evenly spaced row under each murus, baculae in turn subtended by “rhizomate” structure that is weakly attached to the footlayer, the muri thus easily stripping away from the footlayer (Fig. 22 C). The pollen of *Polydora* proves to have a lophate condition with well-defined lophae or muro bearing 4–5 spinules on each segment. The lophae are subtended by columellae in a single series not leaving an ogee-shaped gap in the middle.

Notable secondary metabolites: sesquiterpene lactone glaucolides/hirsutanolies (Bohlmann and Jakupovic 1990, as *Vernonia poskeana* Vatke & Hildebr.), elemanolides, eudesmanolides, secoglaucolides (Herz 1996, as *Vernonia poskeana* Vatke & Hildebr.

*Polydora angustifolia* is the species with which Steetz first introduced the use of pollen structure in the taxonomy of the Asteraceae (Steetz in Peters 1864). The generic name *Crystalopollen* was based on the lophate pattern of the pollen observed by Steetz.

#### Key to species of *Polydora*

**Table:** 

1	Base of the involucre with slender lanceolate bracts, bracts with straight or flexuous apical awns	***Polydora angustifolia***
–	Base of involucre with broad, ovate or oblong bracts, bracts with or without apiculus or mucro	**2**
2	Tips of involucral bracts erect, mostly without distinct apiculate or mucronate apices	***Polydora poskeana***
–	Tips of involucral bracts often with recurved or squarrose apiculate apices	***Polydora steetziana***

#### Polydora angustifolia

Taxon classificationPlantaeAsteralesAsteraceae

(Steetz in Peters) H. Rob., 1999

Crystallopollen angustifolium Steetz in Peters Trise Mossamb., Bot. 2: 366. 1864. (Type B, destroyed); neotype: Malawi. *Brass 16090* (neotype SRGH; isoneotypes K, MO, US; see Wild, Kirkia 11: 55. 1978b). Originally described as Crystallopollen angutifolium forma vulgaris.
Vernonia poskeana Vatke & Hildebr. var. vulgaris (Steetz) Hiern, Cat. Afr. Pl. Welw. I, 3: 519. 1898.
Vernonia erinacea H. Wild, Kirkia 11: 2. 1978, type same as Vernonia poskeana var. vulgaris.
Polydora angustifolia (Steetz in Peters) H. Rob., Proc. Biol. Soc. Wash. 112(1): 232. 1999.

##### Distribution.

Tanzania, Mozambique and Natal east to Malawi, Zambia and Zimbabwe.

#### Polydora poskeana

Taxon classificationPlantaeAsteralesAsteraceae

(Vatke & Hildebr.) H Rob., 1999

Vernonia poskeana Vatke & Hildebr., Oesterr. Bot. Zeitschr. 25: 324. 1875.
Vernonia elegantissima Hutch. & Dalz., Fl. West Trop. Africa ed. 1, 2: 164 (in key), 167. 1931.
Vernonia poskeana Vatke & Hildebr. subsp. bractifimbriata Mendonça, Contrib. Conhec. Fl. Angola, Compositae. 7. 1943.
Vernonia poskeana Vatke & Hildebr. var. elegantisima (Hutch. & Dalz.) C.D. Adams, J. West African Science Assoc. 3(1): 121. 1957.
Vernonia poskeana var. centauroides (Klatt) H. Wild, Kirkia 11(1): 3. 1978.
Vernonia poskeana var. botswanica G.V. Pope, Kew Bull. 41(1): 39. 1986.
Vernonia samfyana G.V. Pope, Kew Bull. 41(1): 42. 1986.
Vernonia poskeana Vatke & Hildebr. subsp. samfyana (G.V.Pope) G.V. Pope, Fl. Zambes. 6(1):148. 1992.
Polydora poskeana (Vatke & Hildebr.) H Rob., Proc. Biol. Soc. Wash. 112(1): 233. 1999.

##### Distribution.

Angola, Botswana, Namibia, South Africa (Transvaal), and Zimbabwe.

#### Polydora steetziana

Taxon classificationPlantaeAsteralesAsteraceae

(Oliv. & Hiern) H. Rob., 1999.

Crystallopollen angustifolium Steetz var. chlorolepis Steetz in Peters, Reise Mossamb. Bot. 2: 366. 1864, non *Vernonia chlorolepis* S. Moore (Angola).
Vernonia steetziana Oliv. & Hiern in Oliv., Fl. Trop Afr. 3: 278. 1877.
Vernonia poskeana Vatke & Hildebr. var. chlorolepis (Steetz) O. Hoffm., Bol. Soc. Brot.10: 171. 1893.
Polydora steetziana (Oliv. & Hiern) H. Rob., Proc. Biol. Soc. Wash. 112(1): 233. 1999.

##### Distribution.

South Africa (Transvaal), Swaziland.

#### Pseudopegolettia

Taxon classificationPlantaeAsteralesAsteraceae

H. Rob., Skvarla & V.A. Funk gen. nov.

urn:lsid:ipni.org:names:77152893-1

**Figure 23.**
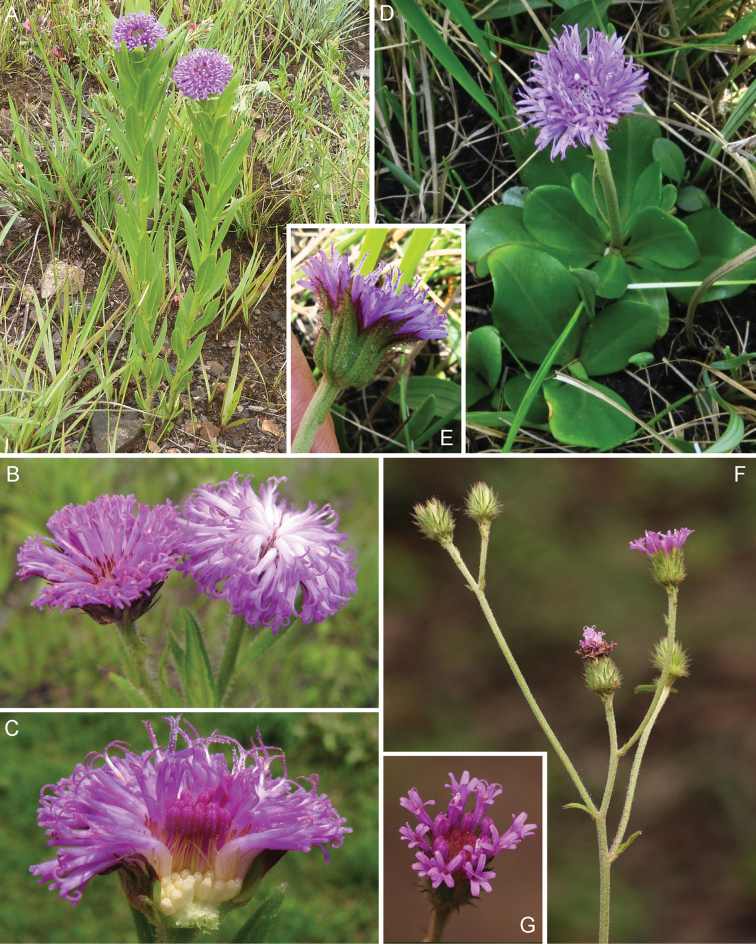
Photographs of *Pseudopegolettia* and *Vernoniastrum*: **A–C** *Pseudopegolettia tenella* (DC.) H. Rob., **D–E** *Pseudopegolettia thodei* (Phillips) H. Rob., Skvarla & V.A. Funk, note: images show the leafy stem of *Pseudopegolettia tenella* and the succulent basal leaves of *Pseudopegolettia thodei*; **F–G** *Vernoniastrum latifolium* (Steetz in Peters) H. Rob.. See Appendix C for citation details.

**Figure 24.**
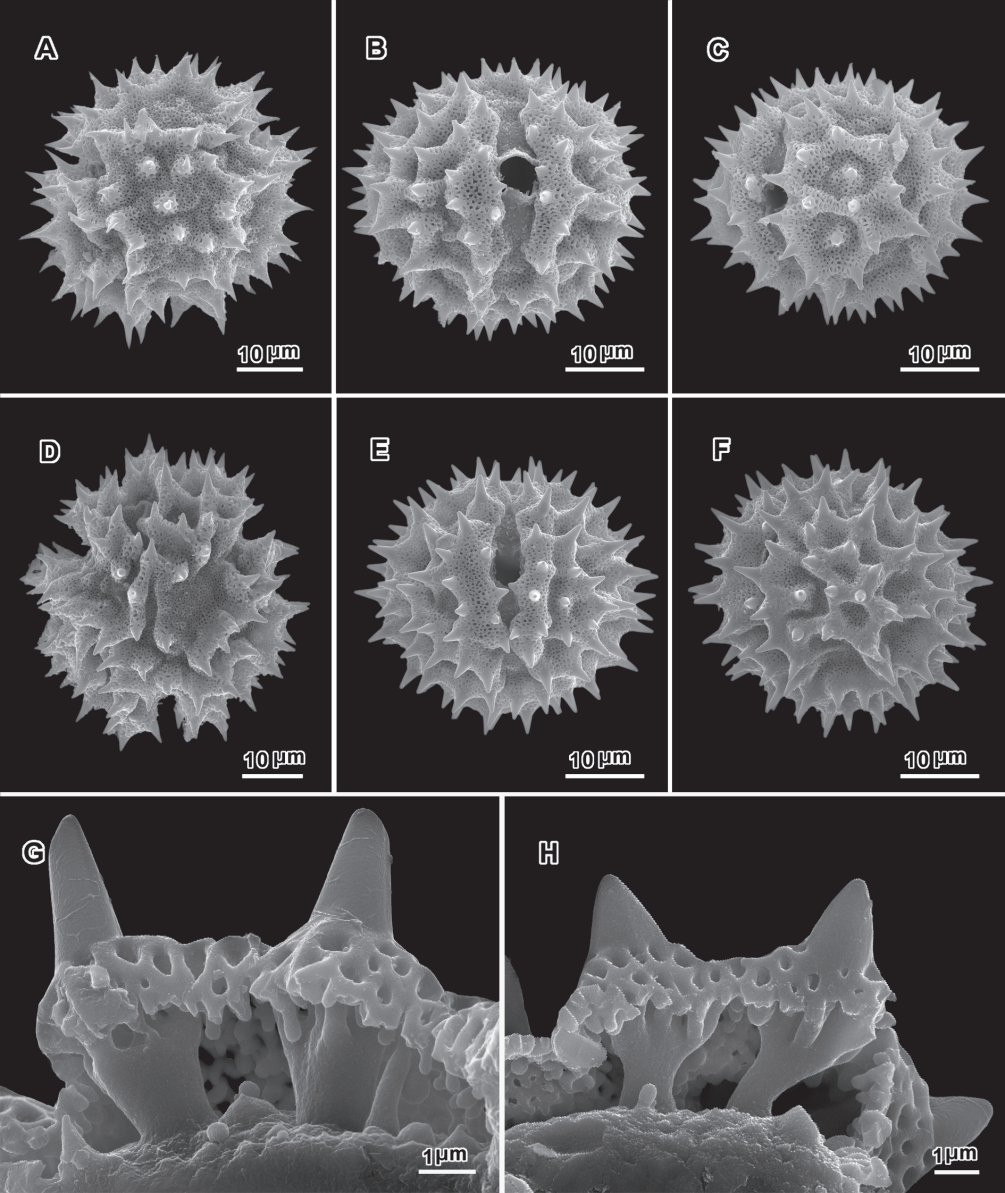
Scanning electron micrographs of *Pseudopegolettia*. **A–H** Scanning electron micrographs of acetolyzed sublophate-echinolophate pollen of *Pseudopegolettia tenella* (DC.) H. Rob. **A** Polar view **B** Equatorial view with prominent lophal ridges surrounding pore **C** Lateral view **D** Polar view, slightly different from A **E** Equatorial view **F** Lateral view slightly different from C **G** and **H** are fractured grains showing the thickened and distally bifurcated support columellae for the overlying exine. From *Sidley 3904*.

Figures 23 A–E ; 24 A–H

##### Type.

*Pegolettia tenella* DC.

##### Descriptions.

Small perennial herbs; stems erect, with short branchlets from lower nodes, puberulous to subsericeous with short-stalked hairs bearing asymmetric cap cells, stalks moderately broad with one or two septae, cap cells short and stout, attached near lower end. Leaves alternate, oblong to linear, essentially sessile, sparsely puberulous, abaxially densely glandular punctate. Inflorescence terminal with 1 or a few heads borne on long peduncles. Heads campanulate, 1.7–2.5 cm. wide; involucral bracts 20–60, in ca. three series, subequal, linear-lanceolate, herbaceous with slender tips, pilosulous outside; receptacle slightly convex, surface with angular thickenings. Florets 15 or more in a head; corollas purple, ca. 1 cm long, narrowly funnel-shaped from a slender base, throat slightly shorter than the moderately distorted, linear-lanceolate lobes, outer surface with short glands on tube and throat, spiculiferous distally on lobes; anther thecae narrowed at base to short lobulate tail; apical appendage glabrous, ovate with rather firm cell walls; style base with narrow annular node; sweeping hairs acicular, restricted mostly to style branches, few on upper shaft. Achenes mostly 6–8-ribbed, to 4.5 mm long, with glandular punctations and scattered idioblasts on sides, rarely without or with many short setulae that are not or scarcely split at apex, inner layer without raphids or with subquadrate raphids, with layer of rather sclerified narrow cells appearing as striations under the glands and idioblasts; carpopodium broadly stopper-shaped, sometimes with few short uniseriate hairs on inner surface; pappus of ca. 40 scabrid bristles, mostly in one series, as long as tube and throat of corolla, rather easily deciduous, scarcely narrowed except at tips, with few indistinct short bristles in outer series. Chromosome number unknown.

Pollen ca. 47 μm in diam., tricolporate, sublophate, echinate, with perforated tectum continuous between colpi (Fig. 24 A–H).

Most notable secondary metabolites include sesquiterpene glaucolides (Bohlmann and Jakupovic 1990 as *Vernonia monocephala* Harv.) and 5-alkylcoumarins (Bohlmann and Jakupovic 1990 as *Vernonia galpinii* Klatt).

The genus consists of mostly monocephalous species, but those species have many individual differences such as the restriction of leaves to a basal rosette, capitula structure, and pubescence of the achenes. They do have essentially identical pollen, but it is a widely distributed pollen type in the Erlangeinae. There are no unique or uncommon characteristics that the two species share.

#### Key to the species of *Pseudopegolettia*

**Table:** 

1	With numerous cauline leaves; heads with many linear outer involucral bracts; achenes with few or no setulae; setulae not divided at tips	***Pseudopegolettia tenella***
–	With leaves mostly basal; heads without linear outer involucral bracts; achenes with many setulae; setulae with shortly but distinctly divided tips	***Pseudopegolettia thodei***

#### Pseudopegolettia tenella

Taxon classificationPlantaeAsteralesAsteraceae

(DC.) H. Rob., Skvarla & V.A. Funk comb. nov.

urn:lsid:ipni.org:names:77152899-1

Pegolettia tenella DC., Prodr. 5: 482. 1836.
Vernonia monocephala Harv. in Harv. & Sond., Fl. Cap. 3: 53. 1865, *nom. illeg*., non *Vernonia monocephala* Gardn. (1847).
Vernonia galpinii Klatt, Bull. Herb. Boiss. 4: 827. 1896.

##### Distribution.

South Africa (Transvaal, Natal).

##### Note.

The older De Candolle name has been placed rather consistently in synonymy, but not adopted. It is only comparatively recently that the combination was occuppied in *Vernonia*, as *Vernonia tenella* D.Nash, Fieldiana, Bot. 36: 74. 1974 = *Lepidaploa tenella* (D.Nash) H. Rob. The epithet *tenella* still has priority in almost all other genera. The DeCandolle specimen is known in this study primarily from synonymy, description, and on the basis of microfiche (IDC DeCandolle Herbarium 197: III: 8).

#### Pseudopegolettia thodei

Taxon classificationPlantaeAsteralesAsteraceae

(Phillips) H. Rob., Skvarla & V.A. Funk comb. nov.

urn:lsid:ipni.org:names:77152900-1

Vernonia collina Schlechter, J. Bot. 1898. 374. 1898, *nom. illeg*., non *Vernonia collina* Gardn. 1846.
Vernonia thodei Phillips, J. Bot., Lond. 74: 205. 1936, based on *Vernonia collina* Schlechter.

##### Distribution.

Zambia, Transvaal (Smith 1971).

##### Note.

The specimen cited by Smith (1971) as *Vernonia nyassae* Oliv. from Transvaal, is *Pseudopegolettia thodei*. The two species have generally similar habits, but they are totally different entities with basically different pollen.

It is evident from the description that *Vernonia collina* of Klatt, based on a Schlechter collection, is not the same as the *Vernonia collina* of Schlechter. See under unplaced species.

#### Vernoniastrum

Taxon classificationPlantaeAsteralesAsteraceae

H. Rob., 1999

**Figure 25.**
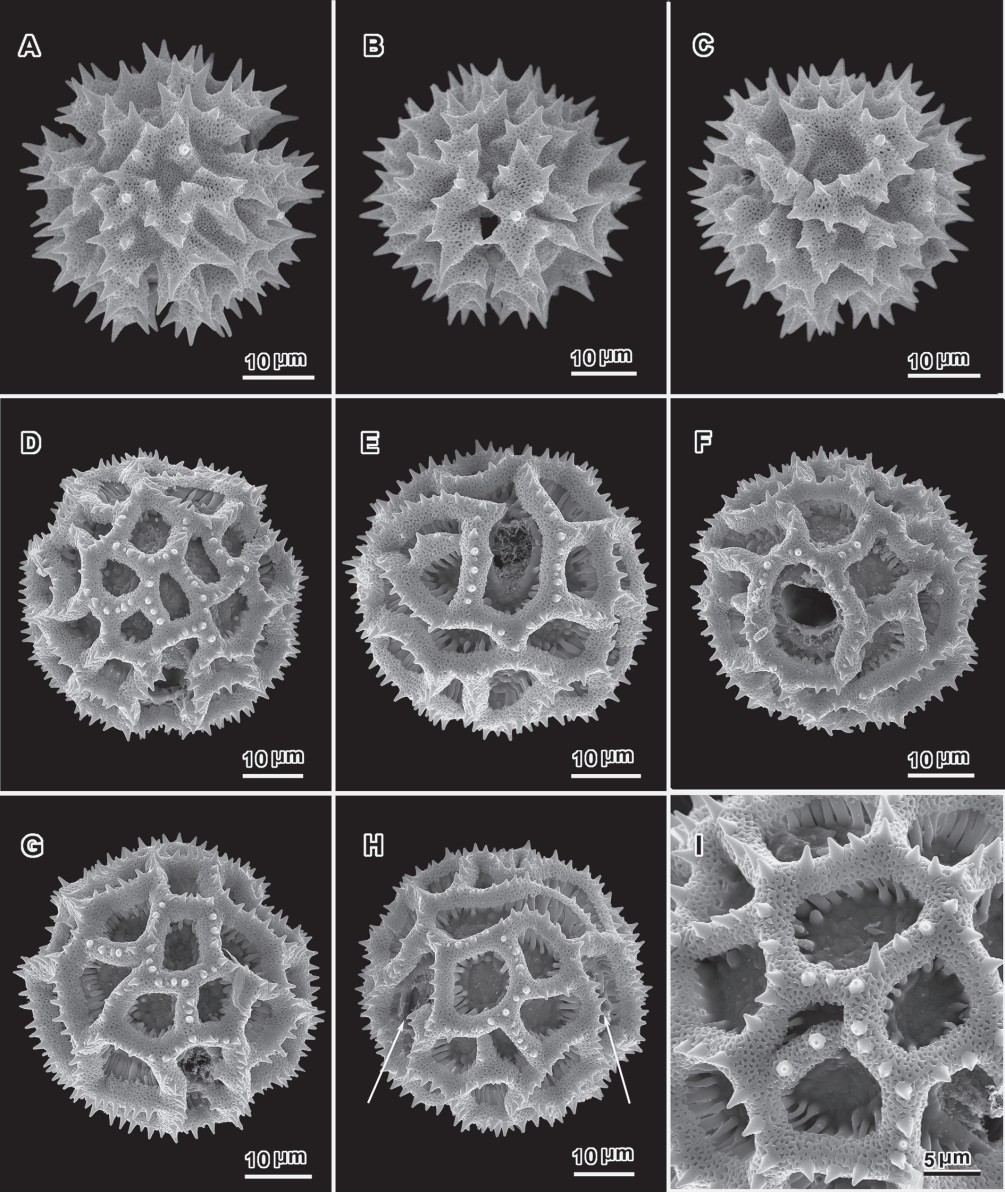
Scanning electron micrographs of *Vernonella* and *Vernoniastrum*. **A–C** Scanning electron micrographs of acetolyzed sublophate-echinolophate pollen of *Vernonella africana* Sond. **A** Polar view **B** Equatorial view **C** Oblique polar view. **A–C** from *Wood 753*. **D–I** Scanning electron micrographs of acetolyzed echinolophate pollen of *Vernoniastrum nestor* (S. Moore) H. Rob., showing diversity of lacunae and lophae. **D–H** Oblique and near polar views showing apertures in markedly long lacunae with irregular lophae **H** Lateral view with apertures (arrows) occupying long lacunar spaces **I** Enlarged section of surface showing columellae in irregular rows under muri). **D–I** from *Reekmans 9185*.

Figures 23 F–G ; 25 D–I

Vernoniastrum H. Rob.. Proc. Biol. Soc. Wash. 112(1): 233. 1999. – Type: *Crystallopollen latifolium* Steetz in Peters.
Vernonia sect. Lepidella Oliv. & Heirn, Fl. Trop. Afr. 3: 267. 1877, non *Lepidella* Tiegh. 1912 or *Lepidella* E.J. Gilbert 1925. – Type: *Vernonia petersii* Oliv. & Hiern.
Vernonia subsect. Lepidella (Oliv. & Hiern) S.B. Jones, Rhodora 83: 72. 1981.

##### Descriptions

. Annual or perennial herbs 0.3–1.0 m tall; stems pilose, hairs simple with elongate apical cells with slightly asymmetric bases. Leaves alternate. Inflorescence with 1–many heads. Involucre campanulate; involucral bracts ca. 50 in ca. three series, gradate, persistent; receptacle epaleaceous. Florets ca. 50 in a head; corollas reddish-purple, basal tube narrowly funnelform, throat shorter than lobes or anther thecae, lobes pilosulous distally; anther bases acuminate to acutely tailed; apical appendage glabrous, with thin cell walls. Style base with node; style branches with acicular sweeping hairs. Achenes 4–6-angled, setulae aparse on sides, idioblasts usually grouped in transverse bands, raphids elongate; pappus bristles subpersistent, marginally densely barbellate; outer squamae persistent. Chromosome number n = 10 (Jones 1979, 1982).

Pollen triporate, lophate, perforated tectum discontinuous in lacunae, muri papillate, with or without micropunctations on muri (Fig. 25 D–I).

With habit similar to *Polydora* but often perennial, lacking L-shaped hairs, having tailed anther bases, and a chromosome number n = 10. Also characteristic of the core element of *Vernoniastrum* are the transverse bands of crowded idioblasts in the achene walls.

#### Key to the species of *Vernoniastrum*

**Table:** 

1	Perennial herbs	***Vernoniastrum nestor***
–	Annual herbs	**2**
2	Apices of involucral bracts straight	***Vernoniastrum acuminatissimum***
–	Apices of involucral bracts recurved	***Vernoniastrum latifolium***

#### Vernoniastrum acuminatissimum

Taxon classificationPlantaeAsteralesAsteraceae

(S. Moore) H. Rob., Skvarla & V.A. Funk comb. nov.

urn:lsid:ipni.org:names:77152901-1

Vernonia acuminatissima S. Moore, J. Linn. Soc., Bot. 40: 104. 1911.
Vernonia rogersii S. Moore, J. Bot. 52: 183. 1913.

##### Distribution.

Tanzania, Mozambique, Zimbabwe.

#### Vernoniastrum latifolium

Taxon classificationPlantaeAsteralesAsteraceae

(Steetz in Peters) H. Rob., 1999

Crystallopollen latifolium Steetz in Peters, Reise Mossamb. Bot. 364, t. 48a. 1864, non *Vernonia latifolia* Lem. 1855.
Vernonia petersii Oliv. & Hiern, Trans. Linn. Soc. London 29: 90. 1873.
Vernonia eriocephala Klatt, Bull. Herb. Boiss. 4: 826. 1896.
Vernoniastrum latifolium (Steetz in Peters) H. Rob., Proc. Biol. Soc. Wash. 112(1): 234. 1999.

##### Distribution.

Angola and Congo east to Mozambique and Tanzania, Namibia.

#### Vernoniastrum nestor

Taxon classificationPlantaeAsteralesAsteraceae

(S. Moore) H. Rob., 1999

Vernonia nestor S. Moore, J. Linn. Soc. Bot. 35: 317. 1902.
Vernoniastrum nestor (S. Moore) H. Rob., Proc. Biol. Soc. Wash. 112(1): 234. 1999.

##### Distribution.

West Africa east to Tanzania, Mozambique, Zimbabwe, Natal.

#### Vernonella

Taxon classificationPlantaeAsteralesAsteraceae

Sond., 1850

Figures 13 D ; 25 A–I

Vernonella Sond., Linnaea 23: 62. 1850. – Type: *Vernonella africana* Sond.

##### Resources.

Species reviewed by Smith (1971) and Robinson and Skvarla (2010a).

##### Descriptions.

Annual or perennial herbs, with leaves rosulate or on leafy stems, basal rosettes often withered at anthesis, bases of plants erect, with or without a dense basal cloak of hairs. Hairs simple or lacking on stems. Inflorescences monocephalic, laxly cymose or densely corymbiform, with short to very elongate peduncles. Heads broadly campanulate; involucres 3–6-seriate, bracts broadly to narrowly oblong, gradate with basal bracts often more lanceolate, tips of inner bracts often obtuse to rounded or apiculate, distally and marginally rather scarious, often purplish. Florets 10–50 or more in a head; corollas purple, with long slender basal tube, throat short, not noticeably broadened at base, lobes linear, usually contorted with age, bearing glands, simple hairs, or L-shaped to T-shaped hairs; anther thecae calcarate and blunt at base, without tails; apical appendage oblong-ovate, with thin cell walls; style base with annulus of thickened, quadrate cells; sweeping hairs slender with sharp, narrow tips. Achenes with ca. 10 ribs, setulose on ribs, setulae with paired cells separated in distal third or less, with numerous idioblasts on surfaces between ribs; raphids in achene wall narrowly elongate. Chromosome number n = 9 (Jones 1982).

Pollen ca. 30–40 μm in diameter when dry, tricolporate with short or truncated colpi, sharply echinate with elongate spines, sublophate with large irregularly shaped lacunae, perforated tectum continuous in lacunae (Fig. 25 A–C).

Notable secondary metabolites include sesquiterpene lactones (elemanolides and eudesmanolides).

The genus *Vernonella* is most notable for its often solitary heads, simple vegetative hairs, the comparatively limited differentiation of the involucral bracts, unexpanded corolla throats, and the comparatively small sublophate rather than lophate pollen with uniquely truncated colpi. On the basis of the examination of the type species, the detailed studies of Smith (1971), and reviews of literature, eleven species are recognized in the genus. The genus is restricted to Africa and is distributed from Cameroon and Sudan in the north southward to Natal in South Africa.

#### One species in the flora area

##### Vernonella africana

Taxon classificationPlantaeAsteralesAsteraceae

Sond., 1850

Vernonella africana Sond., Linnaea 23: 62. 1850.
Vernonia vernonella Harv. & Sond., Fl. Cap. 3: 53. 1865.
Vernonia africana (Sond.) Druce, Bot. Exch. Club Soc. Brit. Isles. 1916: 651. 1917.
Centrapalus africanus (Sond.) H. Rob., Proc. Biol. Soc. Wash. 112: 236. 1999.

###### Distribution.

Natal.

###### Note.

Material of the species was sought by Smith from its type locality, but he reported (1971), “I searched the type locality for living plants, but the area is now devoted to sugarcane fields, and the species may have been completely eliminated.”

#### Species not yet properly placed in a genus

##### Vernonia potamophila

Taxon classificationPlantaeAsteralesAsteraceae

Klatt, 1890

Vernonia potamophila Klatt, Annal. Naturh. Hofmus. Wien 7: 100. 1890.

###### Distribution.

Congo, Angola, Namibia (Caprivi strip), Zambia.

###### Descriptions.

**Figure 26.**
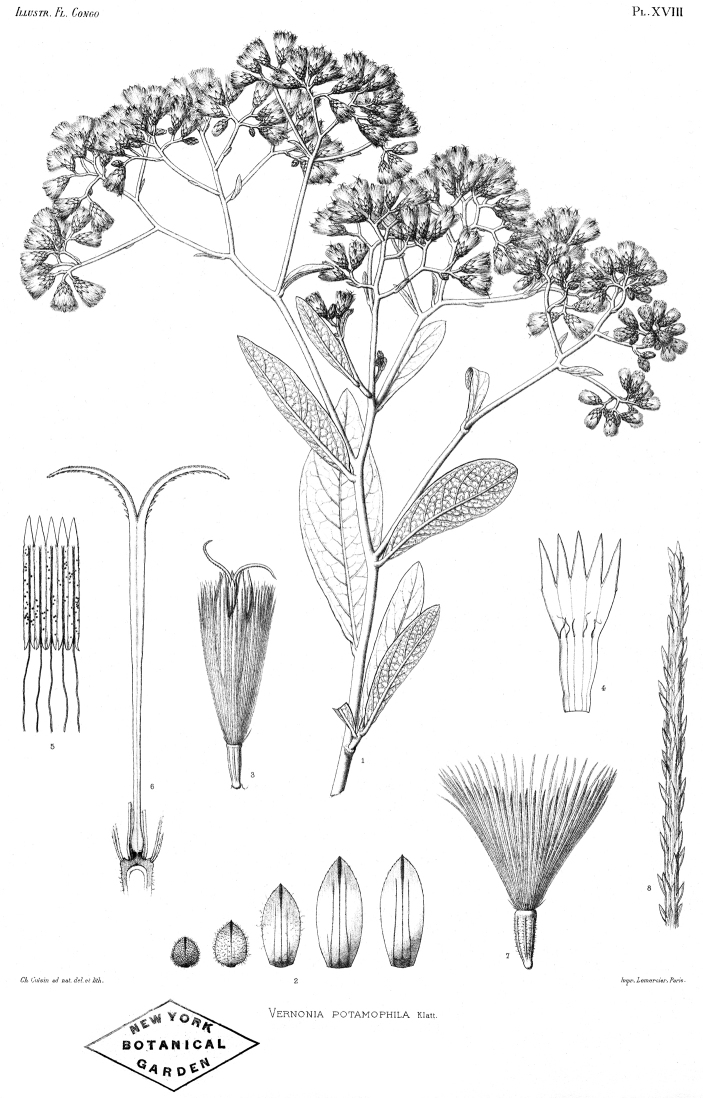
Illustration of *Vernonia potamaphylla* Klatt. See Appendix C for citation details. **1** Habit showing pinnate leaf veination **2** Involucral bracts showing keel and dark line **3** Floret with immature achene **4** Corolla opened longitudinally **5** Anthers **6** Style **7** Achene with pappus **8** Enlarged pappus bristle.

**Figure 27.**
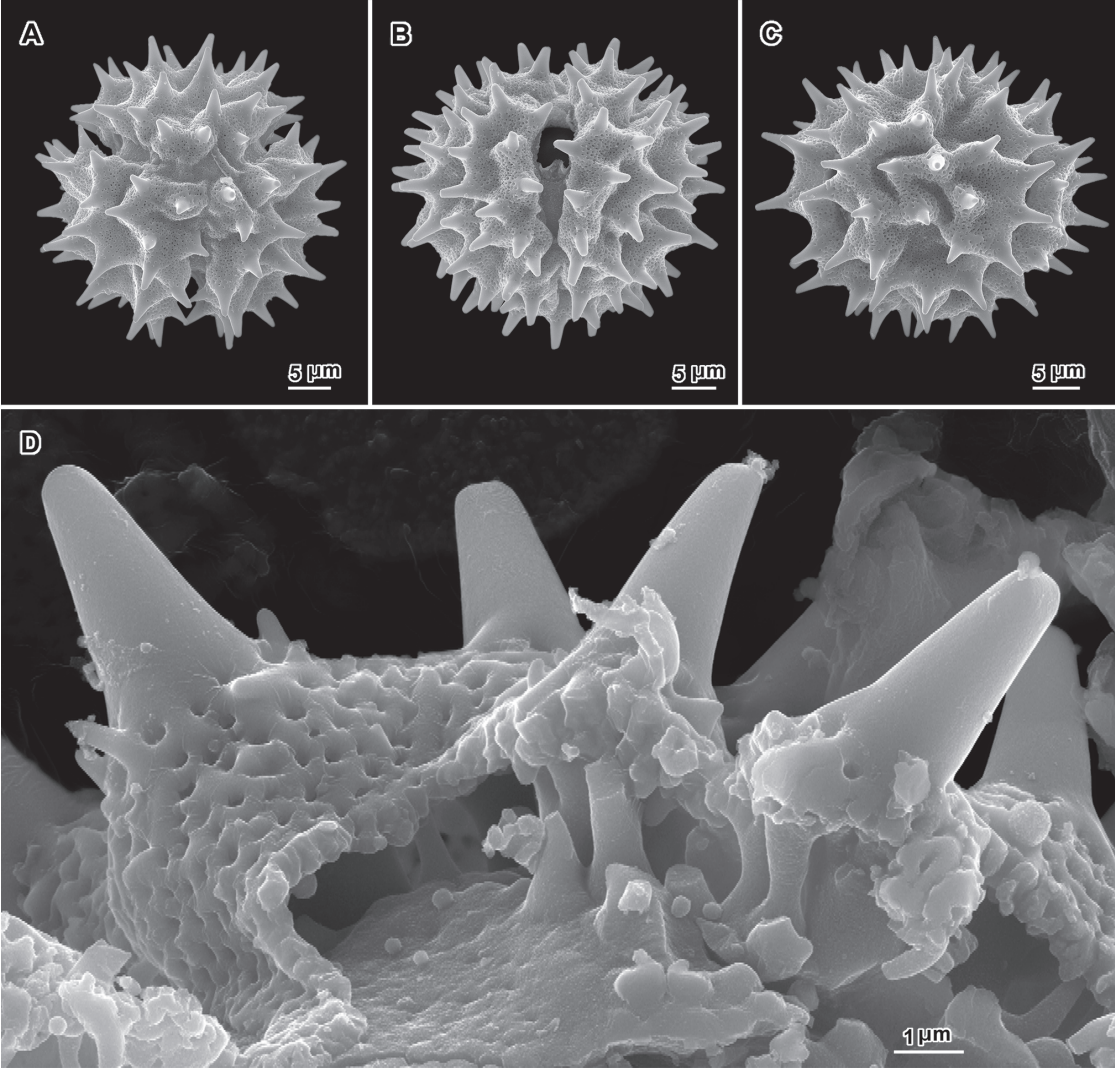
**A–D** Scanning electron micrographs of acetolyzed sublophate pollen of *Vernonia potamaphylla* Klatt. **A** Polar view **B** Equatorial view **C** Lateral view **D** Grain fragment. From *Killick & Leistner 3277*.

The initial assumption, based on the robust habit and the described yellowish brown velutinous pubescence of the stems, was of a relationship to the genus *Gymnanthemum* of the subtribe Gymnantheminae. Other features indicate a different relationship. A high resolution image of an herbarium specimen (PRE; Fig. 2D) as well as an illustration (Fig. 26), show a somewhat keeled involucral bract with a dark median stripe, a character not found in *Gymnanthemum*. In addition, the pollen totally lacks the strongly developed sublophate pattern that is characteristic of *Gymnanthemum* (Figs 10 A–C) and instead is sublophate with small incipient lacunae (Fig. 27). The pollen and involucre characters seem to indicate a position in the subtribe Erlangeinae. This is most likely a new genus but without a more comprehensive study of the more northern members of the African Vernonieae we can only say that *Vernonia potamophila*, while definitely not a true *Vernonia*, is unplaced as to genus.

An examination of limited fragments showed a few additional characters. The abaxial surface of the leaf has a tomentum of long-armed T-shaped hairs and sweeping hairs restricted to the branches of the style and the juncture of the branches at the shaft of the style. The lobes of the corolla had areolae that were reminiscent of the ducts in the corolla lobes of true *Vernonia*, but the areolae do not form continuous elongate ducts. Raphids of the achenes were short-rectangular in elongate cells. Chromosome number unknown.

##### Vernonia collina

Taxon classificationPlantaeAsteralesAsteraceae

Klatt, 1896

Vernonia collina Klatt, Bull. Herb. Boiss. 4: 824. 1896, *nom. illeg.*, non *Vernonia collina* Gardn. 1846.

###### Distribution.

Transvaal (In cliv. Mont. Elandspruitbergen, alt. 7000 ped., leg. R. Schlechter, 2 December 1893, N. 3832).

###### Note.

The name is an illegitimate later homonym and cannot be used, but the species, as described, cannot be placed with other Vernonian species presently known from South Africa. The original description (Klatt 1896) makes no mention of corollas and they may have been absent on the type collection. According to the description, the achenes are ca. 3 mm long and the pappus is 1.5 cm long, suggesting an immature or overly mature condition. From the description it is not certain the species is actually a member of the Vernonieae.

## Supplementary Material

XML Treatment for Baccharoides

XML Treatment for Baccharoides adoensis

XML Treatment for Baccharoides anthelmintica

XML Treatment for Baccharoides benguellensis

XML Treatment for Bothriocline

XML Treatment for Bothriocline laxa

XML Treatment for Cyanthillium

XML Treatment for Cyanthillium cinereum

XML Treatment for Cyanthillium stelluliferum

XML Treatment for Cyanthillium vernonioides

XML Treatment for Cyanthillium wollastonii

XML Treatment for Distephanus

XML Treatment for Distephanus angolensis

XML Treatment for Distephanus angulifolius

XML Treatment for Distephanus anisochaetoides

XML Treatment for Distephanus divaricatus

XML Treatment for Distephanus inhacensis

XML Treatment for Erlangea

XML Treatment for Erlangea misera

XML Treatment for Erlangea remifolia

XML Treatment for Ethulia

XML Treatment for Ethulia conyzoides

XML Treatment for Gymnanthemum

XML Treatment for Gymnanthemum amygdalinum

XML Treatment for Gymnanthemum capense

XML Treatment for Gymnanthemum coloratum

XML Treatment for Gymnanthemum corymbosum

XML Treatment for Gymnanthemum crataegifolium

XML Treatment for Gymnanthemum koekemoerae

XML Treatment for Gymnanthemum myrianthum

XML Treatment for Gymnanthemum theophrastifolium

XML Treatment for Gymnanthemum triflorum

XML Treatment for Hilliardiella

XML Treatment for Hilliardiella aristata

XML Treatment for Hilliardiella capensis

XML Treatment for Hilliardiella flanaganii

XML Treatment for Hilliardiella hirsuta

XML Treatment for Hilliardiella nudicaulis

XML Treatment for Hilliardiella oligocephala

XML Treatment for Hilliardiella pseudonatalensis

XML Treatment for Hilliardiella sutherlandii

XML Treatment for Linzia

XML Treatment for Linzia gerberiformis

XML Treatment for Linzia glabra

XML Treatment for Linzia rosenii

XML Treatment for Namibithamnus

XML Treatment for Namibithamnus obionifolius

XML Treatment for Namibithamnus dentatus

XML Treatment for Oocephala

XML Treatment for Oocephala centaureoides

XML Treatment for Oocephala staehelinoides

XML Treatment for Orbivestus

XML Treatment for Orbivestus cinerascens

XML Treatment for Parapolydora

XML Treatment for Parapolydora fastigiata

XML Treatment for Parapolydora gerrardii

XML Treatment for Polydora

XML Treatment for Polydora angustifolia

XML Treatment for Polydora poskeana

XML Treatment for Polydora steetziana

XML Treatment for Pseudopegolettia

XML Treatment for Pseudopegolettia tenella

XML Treatment for Pseudopegolettia thodei

XML Treatment for Vernoniastrum

XML Treatment for Vernoniastrum acuminatissimum

XML Treatment for Vernoniastrum latifolium

XML Treatment for Vernoniastrum nestor

XML Treatment for Vernonella

XML Treatment for Vernonella africana

XML Treatment for Vernonia potamophila

XML Treatment for Vernonia collina

## Appendix A: Definitions of Pollen Terms (as used in this paper)

As a general rule Vernonieae pollen can be ‘nearly non-lophate’ to ‘sublophate’ to ‘lophate’, however, none of the grains in the Vernonieae are completely non-lophate. Grains can be with or without colpi and they can be tricolporate or triporate to pentaporate.

*Columellae*: A rod-like element supporting the tectum.

*Echinolophate*: Among lophate types, one variant has prominent lophae (muri) with sharply projecting spines. The lophae have supporting thickened columellae or baculae.

*Lacunae*: Areas in lophate pollen surrounded by muri.

*Lophate* (in the Vernonieae): Pollen having the perforated tectum non-continuous. There are varying degrees of loss of perforated tectum. Grains can have spines (various types of echinolophate) or not (psilolophate).

*Muri*: partitions or walls forming a network on surface of lophate pollen (Figs 3 A–I, 4 A–C, and 4 D–F).

*Nearly non-lophate*: Arrangement of spines is somewhat uneven and the perforated tectum is continuous.

*Non-lophate* (truly): Completely evenly spaced spines or columellae; the perforated tectum is continuous. [not found in Vernonieae]

*Pantoporate*: Pores on pollen positioned all over the surface, not strictly equatorial.

*Psilolophate*: A variant of the lophate types where the surface of the lophae is without spines [Linzia]

*Sublophate* (as used in the Vernonieae): The arrangement of the spines is somewhat to distinctly uneven, leaving incipient lacunae; the perforated tectum is always continuous in all non-colpar areas supported by massive columellae or baculae, and spines being almost always present over the baculae.

*Sub-echinolophate*: There are varying forms of lophate grains that are highly perforate and spinose.

*Tectum*: The layer that forms a roof over the columellae. A perforated tectum has perforations smaller than 1μm in diameter

*Tricolporate*: Grains with three colpi and a central pore in each colpus. The pore provides a germination point for the emerging pollen tube.

*Triporate*: Grains without colpi and with three equatorial pores for possible germination.

## Appendix B: List of specimens used for pollen images

*Baccharoides anthelmintica* (L.) Moench. Assam, *Koelz 7469* (US)

*Baccharoides anthelmintica* (L.) Moench., *USDA P.I. 283729*, chromosome voucher, Jones (US)

*Baccharoides anthelmintica* (L.) Moench., Ceylon (Sri Lanka), 17 March 1970, *G. Cooray 70031701R* (US)

*Bothriocline schimperi* Oliv. & Hiern ex Benth., Ethiopia, Wllega Prov. 6 Feb. 1962, *F. Meyer 8159* (US)

*Cyanthillium cinereum* (L.) H. Rob., Pacific, Caroline Islands, Fais Isl. *Evans 344* (US)

*Distephanus angulifolius* (DC.) H. Rob. & B. Kahn, S. Afr., Natal, *Sidley 2211* (US)

*Erlangea misera* (Oliv. & Hiern) S. Moore, Namibia, Popa Falls, 21 Aug 2007, *Funk 12708* (US)

*Ethulia conyzoides* L.f., Uganda, *W.H. Lewis 6025* (US)

*Ethulia conyzoides* L.f., Vietnam, Tonkin, 19 June 1927, *Petelot 4047* (US)

*Gymnanthemum mespilifolium* (Less.) H. Rob. Plantae Africae Australe, *Schlechter 6644* (US)

*Hilliardiella capensis* (Houtt) H. Rob., Skvarla & V. Funk, S. Africa, Cape Province, *Gentry & Barolas 18914* (US)

*Hilliardiella capensis* (Houtt) H. Rob., Skvarla & V.A. Funk, Cape Prov., Humansdrap Div., *Bayliss BS 3686* (US).

*Linzia glabra* Steetz, Zimbabwe, *O. West 7292* (US)

*Linzia rosenii* (R.E. Fries) H. Rob., Skvarla & V.A. Funk, Botswana, Savuti, Chobi Natl. Park, *Jacobsen 3075* (PE).

*Namibithamnus obionifolius* (O. Hoffm.) H. Rob., Skvarla & V. Funk, Namibia, *Tõlken & Hardy 770* (US)

*Oocephala centaurioides* (Klatt) H. Rob. & Skvarla, Regio oriente et Mosambique, Delagoa Bay, *Schlechter 18138* (US)

*Oocephala staehelinoides* (Harv.) H. Rob. & Skvarla, Transvaal, *Liebenberg 8843* (US)

*Orbivestrus cinerascens* (Sch. Bip. in Schweinf.) H. Rob. Transvaal, *Koekemoer 232* (US)

*Parapolydora fastigiata* (Oliv. & Hiern) H. Rob., Namibia, Vindhuk Bergland 13.5.1964, *Seydel 4023* (US)

*Parapolydora fastiagiata* (Oliv. & Hiern) H. Rob., Tranvaal, *B.J. Pienaar 1073* (US)

*Polydora angustifolia* (Steetz) H. Rob. (as erinacea). Malawi: Zomba District, Likangala, Phalombe Road. 3000 ft. 3/ 12/ 1984. *Christenson & Patel GMC 1357* (US)

*Polydora angustifolia* (Steetz) H, Rob., *Brass 16090* (US)

*Pseudopegolettia tenella* (DC.) H. Rob., Skvarla & V. Funk, South Africa: Natal, *Sidley 3904* (US)

*Pseudopegolettia thodei* (Phillips) H. Rob., Skvarla & V.Funk, *Hilliard & Burtt 8374* (PRE)

*Pseudopegolettia thodei* (Phillips) H. Rob., Skvarla & V. Funk, *Koekemoer 2117* (PRE)

*Vernonella africana* Sond. Natal, S. Africa, *Wood 753* (US)

*Vernonia potamophila Klatt*, *SW Africa*, *Caprivi*, *Killick & Leistner 3277* (PRE)

*Vernoniastrum nestor* (S. Moore) H. Rob., Burundi, Prov. Burundi, Gihofi (Mgsso) jachere, 20 May 1980, *Reekmans 9185* (US)

## Appendix C: Sources for photographs, illustrations, and websites [Plant names are as they were found on the image and may not reflect current taxonomy]

**Figure 1**:

A. Blittersdorff R von (2009) “*Vernonia adoensis* Sch. Bip. ex Walp. (photo ID: 23949)”. S. Dressler S, Schmidt M, Zizka G, eds. (2014) African Plants, a photo guide. Forschungsinstitut Senskenberg, Frankfurt/Main, Germany. http://www.africanplants.senckenberg.de [date accessed: 23 December 2014]

B. Hyde M (2014) “*Vernonia adoensis* Sch. Bip. ex Walp. (Record no: 58514)”. Hyde MA, Wursten BT, Ballings P, Coates Palgrave M (2014) Flora of Zimbabwe: species information: individual images. http://www.zimbabweflora.co.zw [date accessed: 23 December 2014]

C. Hyde M (2014) “ *Bothriocline laxa* N.E. Br. (Record no: 158050)”. Hyde MA, Wursten BT, Ballings P, Coates Palgrave M (2014) Flora of Zimbabwe: species information: individual images. http://www.zimbabweflora.co.zw [date accessed: 23 December 2014]

D. *Vernonia potamophila Klatt* [Image of specimen from PRE]

**Figure 2**:

A. Smith CE, Jr (1971) ”Vernonia adoensis Sch. Bip. Ex Walp., illustrated by Hughes RO”) Observations on stengelioid species of Vernonia. USDA (ed.), Agriculture Handbook No. 396, Washington DC, United States Department of Agriculture. 87 Pp.

B. Prain D (1918) “Erlangea aggregata Hutch., illustration no 8755 by Smith M” Curtis’s Botanical Magazine 144 [ser. 4, vol. 14]: t. 8269

C. Anonymous (1832). “*Ethulia conyzoides* L.f., illustrated by M. Hart” Botanical Register, vol. 9 t. 698.

D. Robinson H, Funk VA (2014) Gymnanthemum koekemoerae H.Rob & V.A. Funk, illustrated by A. Tangerini. PhytoKeys: 36: 61.

**Figure 5**:

A–C. LeBourgeois T (2008) “Cyanthillium cinereum (L.) H. Rob.” Pl@ntNet Web: http://www.plantnet-project.org/ [date accessed: 23 Dec 2014]

**Figure 6**:

A–C. Wursten BT (2014) “*Distephanus divaricatus* (Steetz) H. Rob. & B. Kahn. (Record no: 60279, 60281, 60282)”. Hyde MA, Wursten BT, Ballings P, Coates Palgrave M (2014) Flora of Zimbabwe: species information: individual images. http://www.zimbabweflora.co.zw [date accessed: 16 December 2014]

D. Creative Commons - Attribution Non-Commercial Share-Alike. *Distephanus divericatus* (Steetz) H. Rob. & B. Kahn. 2011. License Holder: Olivier Maurin, University of Johannesburg, Johannesburg. BOLD Systems. http://www.boldsystems.org/index.php/Taxbrowser_Taxonpage?taxid=349118 [date accessed: 22 Dec 2014]

E–F. English P (2010) “*Distephanus divericatus* (Steetz) H. Rob. & B. Kahn. (record no: 43443 & 43445).” Web: www.zambiaflora.com [date accessed: 22 Dec 2014]

G. Graham G (2013) “*Distephanus anisochaetoides* (Sond.) H. Rob. & B.Kahn (image no: 531616). iSpot, share nature. http://www.ispotnature.org [date accessed: 19 Dec. 2014]

H. Anonymous (2012) “Distephanus anisochaetoides (Sond.) H. Rob. & B.Kahn (image #521927 by ES). iSpot, share nature. http://www.ispotnature.org [date accessed: 19 December 2014]

**Figure 7**:

A–B. Schneider B (2013) “*Erlangea misera* S. Moore (image no. 41706)”. Web: http://www.ispotnature.org/node/508454 *accessed date*: 2 March 2015

C–D. Wursten BT (2014) “Ethulia conyzoides L.f. subsp. conyzoides. (Record no: 40831 & 40832)”. Hyde MA, Wursten BT, Ballings P, Coates Palgrave M (2014) Flora of Zimbabwe: species information: individual images. http://www.zimbabweflora.co.zw [date accessed: 16 December 2014]

E. Bidault, E. (2015) “*Ethulia conyzoides* L.f.”. http://tropical.theferns.info/image.php?id=Ethulia+conyzoides [date accessed: 23 August 2015]

**Figure 9**:

A. Villiers F de (2014) “*Gymnanthemum corymbosum*”. iSpot, share nature. http://www.ispotnature.org [date accessed: 23 December 2014]

B–D. Berkel, N van (2013) “*Gymnanthemum mespilifolium*”. iSpot, share nature. http://www.ispotnature.org [date accessed: 23 December 2014]

**Figure 11**:

A–B. Blittersdorff R von (2008-9) “*Vernonia natalensis* Sch. Bip. ex Walp. Dressler S, Schmidt M, Zizka G (eds.) (2014) African Plant, a photo guide. Forschungsinstitut Senskenberg, Frankfurt/Main, Germany. http://www.africanplants.senckenberg.de [date accessed: 23 December 2014]

C–D. Warren PR (2013) “*Hilliardiella flanaganii*”. iSpot, share nature. http://www.ispotnature.org [date accessed: 23 December 2014]

**Figure 12**:

A–D Berkel N van (2012) “*Hilliardiella oligocephala*”. iSpot, share nature. http://www.ispotnature.org [date accessed: 23 December 2014]

**Figure 13**:

Hooker WJ (1863) “*Webbia pinifolia* [*Hilliardiella capensis*] illustration 5412 by W.H. Fitch.” Curtis’s Botanical Magazine 89 [ser. 3, vol. 19]: t. 5412.

Humbert H (1960) Composees. “Linzia glabra Steetz in Peters, Illustrated by J.V. & .G.M.” [as *Vernonia obconica* (Oliv. & Hiern in Oliv.)] in Humbert H (ed.), Flore de Madagascar et des Comores, Family 189, Tome 1, Fig. VI, Paris, Typographie Firmin-Didot et Cie. http://www.biodiversitylibrary.org/bibliography/6600#/summary

Wood JM (1906) “*Vernonia gerrardi* Harv., illustrated by M.F.” Natal Plants 4: t. 330.

Wood JM (1906) “*Vernonia vernonella* Harv., illustrated by M.F.” Natal Plants 4: t. 332

**Figure 15**:

A–B. Dressler S, Schmidt M, Zizka G (2014) “*Linzia glabra* Steetz in Peters” Introducing African Plants – A Photo Guide – An interactive photo data-base and rapid identification tool for continental Africa. Taxon 63(5): 1159–1161.

C–D. Strohbach B (2014) “*Vernonia obionifolia* O. Hoffm. (photo id: 16480 & 16478)”. Jürgens N, Strohbach B, Schmiedel U, Rügheimer S, Erb E, Wesuls D, Schrenk J, Dreber N, Schmidt M, Mayer C, Zizka A, Horn P, Mills A, Etzold S, Schulz A, Beaumont J, Oncken I, Revermann R, Niesler I, Kwembeya E, Deckert J, Kuhlmann M, Reddig C, Miehlich G, Christiaan R, Finckh M, Kruger S, Coetzee M, Fortuin A, Ihlenfeldt H, Le Roux A, Erb P, Groengroeft A, Helme N (2014) Photo Guide to Plants of Southern African plants. BioCentre Klein Flottbek, Hamburg, Germany, www.southernafricanplants.net

**Figure 17**:

A–B. Taylor R (2014) “Oocephala (Vernonia) centaureoides (photographs)”. iSpot, share nature. http://www.ispotnature.org [date accessed: 23 December 2014]

C. Wursten BT (2007) “*Vernonia cinerascens* Sch. Bip. (Record no: 22771)”. Hyde MA, Wursten BT, Ballings P, Coates Palgrave M (2014) Flora of Zimbabwe: species information: individual images. http://www.zimbabweflora.co.zw [date accessed: 16 December 2014]

D–E. Dreyer, A. (2015). “*Vernonia cinerascens* Sch. Bip (Record no: 8879 & 8878)“ Kyffhäuser. [online] Kyffhauser.com. Available at: http://www.kyffhauser.com [date accessed: 5 Jan 2015]

**Figure 20**:

A–C. Warren, P.R. (2014) “*Vernonia fastigiata* Oliv. & Hiern”. iSpot, share nature. http://www.ispotnature.org. [date accessed: 23 December 2014]

D–F. Wursten, B.T. (2014) “*Polydora poskeana* (Vatke & Hildebrandt) H. Rob. (Record no: 25475, 25476)”. Hyde MA, Wursten BT, Ballings P, Coates Palgrave M (2014) Flora of Zimbabwe: species information: individual images. http://www.zimbabweflora.co.zw [date accessed: 23 December 2014]

**Figure 23**:

A. Hankey A (2013) “*Vernonia galpinii* Klatt”. iSpot, share nature. http://www.ispotnature.org. [date accessed: 23 December 2014]

B–C. Blittersdorff R von (2008) “*Vernonia galpinii* Klatt (photo ID: 24044, 24049)”. S. Dressler, Schmidt, M., Zizka, G., eds. (2014) African Plant, a photo guide. Forschungsinstitut Senskenberg, Frankfurt/Main, Germany. http://www.africanplants.senckenberg.de. [date accessed: 23 December 2014]

D–E Warren PR (2013) “*Vernonia thodei*”. iSpot, share nature. http://www.ispotnature.org. [date accessed: 23 December 2014]

F–G. Wursten BT (2010) “*Vernonia petersii Oliv. & Hiern ex Oliv.* (Record no: 40742, 40745)”. Hyde MA, Wursten BT, Ballings P, Coates Palgrave M (2014) Flora of Zimbabwe: species information: individual images. http://www.zimbabweflora.co.zw [date accessed: 16 December 2014]

**Figure 26**

A. Wildeman E, Durand T (1898) “*Vernonia potamophila Klatt*” (drawn & lithographed from nature by Ch. Cuisin) Illustrations de la flore du Congo 1: 35. PL XVIII
